# The development of preterm infants from low socio-economic status families: The combined effects of melatonin, autonomic nervous system maturation and psychosocial factors (ProMote): A study protocol

**DOI:** 10.1371/journal.pone.0316520

**Published:** 2025-01-10

**Authors:** Theano Kokkinaki, Nicole Anagnostatou, Maria Markodimitraki, Theano Roumeliotaki, Manolis Tzatzarakis, Elena Vakonaki, Giorgos Giannakakis, Aristidis Tsatsakis, Eleftheria Hatzidaki

**Affiliations:** 1 Child Development and Education Unit, Laboratory of Applied Psychology, Department of Psychology, University of Crete, Rethymnon, Crete, Greece; 2 Department of Neonatology/Neonatal Intensive Care Unit, University Hospital of Heraklion, School of Medicine, University of Crete, Heraklion, Crete, Greece; 3 Department of Preschool Education, University of Crete, Rethymnon, Crete, Greece; 4 Department of Social Medicine, School of Medicine, University of Crete, Heraklion, Crete, Greece; 5 Laboratory of Toxicology, School of Medicine, University of Crete, Heraklion, Crete, Greece; 6 Institute of Computer Science, Foundation for Research and Technology Hellas, Heraklion, Crete, Greece; 7 Department of Electronic Engineering, Hellenic Mediterranean University, Chania, Crete, Greece; PLOS: Public Library of Science, UNITED KINGDOM OF GREAT BRITAIN AND NORTHERN IRELAND

## Abstract

Preterm births constitute a major public health issue and a chronic, cross-generational condition globally. Psychological and biological factors interact in a way that women from low socio-economic status (SES) are disproportionally affected by preterm delivery and at increased risk for the development of perinatal mental health problems. Low SES constitutes one of the most evident contributors to poor neurodevelopment of preterm infants. Maternal perinatal mental health disorders have persistent effects on behavioral and physiological functioning throughout the lifespan and may even be evident across generations. The overall objective of the proposed longitudinal, multi-disciplinary and multi-method study is to compare the association of psychosocial (maternal mental health, intersubjectivity, attachment, family functioning, dyadic coping and perceived social support), and biological factors (melatonin and heart rate variability) with preterm infants’ development at 9 months (corrected age), between low and high SES families. We will collect data from preterm neonates (<37 weeks gestational age) hospitalized in the Department of Neonatology/Neonatal Intensive Care Unit of the University General Hospital of Heraklion, Greece, and their mothers. Data collection of psychosocial and biological factors will be carried out at birth, and at the corrected age of 6 and 9 months, while preterm infants’ cognitive and social development will be assessed at 9 months corrected age. The findings of this study may highlight the need for early interventions for new mothers coming from low SES in order to promote their preterm infants’ optimal early neurodevelopment and for community-evidence-based prevention efforts to restrict the cycle of health inequities and intergenerational mental disorders.

## Introduction

Women from low SES and in low-income countries are disproportionally affected by preterm delivery, perinatal depression/anxiety, and lack access to mental health care. This results in economic and social hardships that affect individuals, families and society as a whole, furthering the cycle of poverty and health inequities [1]. The implications of this study may highlight the need to advance the debate about the responsibility of society to counteract health inequalities [2] and to interrupt the pattern of intergenerational mental illness and suffering [1]. This is important because the perinatal mental health literature has focused on individual women as the main agent for change while social determinants of mental health, such as poverty, are of critical importance for women in the perinatal period [3]. At the economic level, identification of early risk factors for the development of preterm infants may has implications for policymakers since preterm infants incur higher early intervention costs [4].

### The theoretical background of the study

#### The developmental origins of emotional and behavioral development

The foetal programming hypothesis and the Developmental Origins of Health and Disease paradigm (DOHaD) postulates that the environmental influences during critical periods of development, such as the period from conception to early childhood, may alter the trajectory of development, with significant consequences for an individual’s short and long-term health, and may explain neuropsychiatric diseases [5–9]. Critical perinatal factors influencing organogenesis and predisposition to disease include genetic factors, the interaction between genes and environment, duration of gestation and maternal-fetal interactions [9]. In particular, prenatal stress provokes adaptive changes in endocrine and metabolic processes that become permanently programmed and impact later adult health [10]. What is more, the Developmental Origins Theory was proposed to explain the observations linking early life events, such as prematurity, with later adult pathology [11, 12]. A ‘preterm behavioral phenotype’ is characterized by increased risk for symptoms and disorders associated with anxiety, social difficulties, behavioral and developmental delays and an increased prevalence of autism spectrum disorders [13].

#### The developmental context of preterm infants from low SES families

Biological, psychological and sociological risks interact in a way that threatens the family’s ability of low SES to respond to the physical, social, and emotional needs of its members [14]. Socioeconomic disadvantage and risk factors are associated with preterm birth, low gestation age and a birthweight below the 10^th^ percentile [15]. Higher prevalence of biological risks of preterm children in socio-economically disadvantaged environments puts them at ‘double jeopardy’ for poor developmental outcome [16, 17]. Although developmental delays of premature infants and those from low SES are well-recognized, there is limited evidence on the very early postpartum development of infants exposed to both biological and environmental risk factors [18]. It is likely that poverty begins to impact outcome at the earliest stages of development, even prenatally [14, 19–21]. Premature infants of low SES have greater long-term developmental morbidity than do premature infants of higher SES [22]. In the course of the first 24 months, socioeconomic status is responsible for the variance in the cognitive development of preterm infants: the higher the SES, the higher the cognitive development of preterm infants [16]. Preterm infants from low SES are at increased risk of behavioral and emotional problems at age 4 years compared to those with high SES [23].

### The rationale of this study

#### Psychosocial factors related to the development of preterm infants from low SES families

*Μaternal mental health*. Both anxiety and depression during pregnancy has been associated with preterm birth and they interact with aspects of low SES [24]. Stress of parents of preterm infants is high and families of low SES may face additional challenges [25]. Antenatal anxiety is more prevalent in women of low compared to high SES [26]. Low-income women are at increased risk for the development of postpartum depression symptoms in the perinatal period and they are less likely to receive appropriate treatment for depression during the postpartum period [27, 28]. This possibly contributes to a prolonged experience of symptoms [29] with long-term negative physical and psychological health outcomes for both the mother and the infant [30].

*Family functioning*. Low-income status has been associated with less satisfaction related to several areas of family functioning [31] and with stressful family life situations and may exacerbate emotional distress for both partners in the relationship with adverse effects for adult psychological well-being [32, 33]. Low-income families of preterm infants are disproportionately burdened by stress and mothers experience significant anxiety in the perinatal period [25]. Family sense of coherence, that is the emotional bond that family members have towards another, can potentially affect how couples adapt to the transition to parenthood. Couples with a strong family sense of coherence probably share a common goal in bringing up a child and are motivated to mobilize all available resources to deal with the parental demands [34]. Concurrent and longitudinal relationship between cohesiveness and child adjustment show that children in families with high levels of cohesiveness are less likely to have emotional and behavioral adjustment problems [35–37].

*Support resources*. During the transition to parenthood, dyadic coping reduces partner’s distress, it improves his/her psychological well-being and enhances couple functioning [38, 39]. Social support to parents of preterm infants may function as a protective factor for the family unit [40, 41]. Lack of social relations constitutes one of the stressors faced by low SES families compared to high SES families [40, 42]. The risk of postpartum mental disorders is higher among low SES women who had insufficient social support [43]. Social support in the postpartum period has a direct positive correlation with family function and an indirect negative correlation with depression [44]. Social support networks significantly influence a child’s socioecological context and infant development may be associated with maternal social support. However, this relationship has not been well-examined under poverty conditions especially in the first 9 months of life [45].

*Intersubjectivity and parent-to-infant attachment*. Human intersubjectivity is ‘a process that makes it possible for subjects to detect and change each other’s mind and behaviour, by purposeful, narrative expressions of emotion, intention and interest’ [46, p.18]. Maternal perception of the infant’s participation in the mother–infant relationship is a relevant contribute to the development of this important experience. Due to preterm birth, early mother–infant intersubjective interactions may be compromised [47]. The absence of intersubjective communication in spontaneous mother–infant interaction interferes with the development of socio-emotional competences associated to neuro-developmental disorders [48]. Preterm infants are a high-risk group regarding the formation of attachment [40, 49]. Family cohesion is closely related to attachment [50]. Within the first year post discharge from Neonatal Intensive Care Unit (NICU), a higher level of maternal depressive symptoms is associated with preterm birth, poor attachment and poor social support. Surprisingly, in the first year postcharge from NICU, there is evidence of high mother-to-infant attachment for mothers with preterm infants [40]. However, some mothers of preterm infants describe mixed emotional experiences and an inner struggle with their bonding process to their children [51]. The combination of premature birth and low SES may affect the quality of attachment and may hinder parental bonding with poor possible developmental difficulties [52, 53].

#### Biological factors related to the development of preterm infants from low SES families

*Melatonin*. Besides regulating the circadian rhythm, melatonin has a wide range of biological functions, including antioxidation, anti-inflammatory, anti-apoptosis, immunomodulatory, and gut microbiota formation, making it a potent bioactive molecule with long-term cardiovascular health effects, especially in infants [54–56]. Melatonin detected in the fetus originates from the mother (trans-placental tranfer) and the levels rise beginning at 24 weeks gestation, reaching a peak in the third trimester. During the first 3 months approximately the infant will experience a transient deficiency in melatonin, due to suboptimal melatonin production and immature circadian rhythmicity [57] and an impaired antioxidant system [58]. This property has special importance in preterm newborns, since preterm birth leads to sudden interruption of transplacental transfer of melatonin that normally takes place during the last part of the pregnancy, prematurity itself poses a greater risk for oxidative stress and premature neonates exhibit a delay in the rhythmic expression of melatonin with respect to full-term infants. Few studies showed that preterm newborns have an absent, lower or delayed melatonin production, precisely in the period they would have a great need for its antioxidant activity, for the maturation of their central nervous system. Breastmilk is therefore the only source of melatonin for the infant, and especially the preterm neonate, during the first few months of life [56, 58]. Melatonin in human milk is important for normal neurodevelopment, it plays an important role in newborn synchronization with the mother’s rhythm, it entrains rhythms in the cardiovascular system that are essential to neonatal homeostasis and may contribute to better growth and long-term neurodevelopment [56].

Though it is unclear to what extent socioeconomic status influences breast milk composition and circadian rhythm variation [59, 60], lower socioeconomic status may exacerbate the risk of poor nutritional and health status in pregnant women [61] and nutritional deficiencies can impact circadian rhythmicity in human milk composition [59].

Maternal postpartum stress and negative mood have been associated with higher melatonin in milk samples [62] although laughter increased the levels of breast-milk melatonin in healthy mothers [63]. However, preterm breast milk has a higher concentration of melatonin than term breast milk [55], suggesting that breast milk may provide a protective tool to compensate for the lack of melatonin due to premature birth [56]. An indirect effect between melatonin and family environment is implied only by limited evidence showing that interventions to improve sleep of ASD children had also a benefit on family functioning [64]. The role of melatonin for preterm infant development is unclear [65] and under-investigated. To our knowledge, only one study showed that improved autonomic function at 2 weeks of age was associated with higher Mental Developmental Index scores at 9 months when related to the amount of melatonin at 4, 6, and 9 months of age [66]. It is important to study the way maternally-derived melatonin is involved in early development because melatonin disturbances have been reported in child and adult psychiatric disorders, such as major depressive disorder, bipolar disorder, schizophrenia and autism spectrum disorder [67].

*Autonomic nervous system maturation and heart rate variability*. In infants born at term, the normal increase in parasympathetic tone is evidenced in the increased high-frequency heart rate variability (HRV) [68]. Gestational age in preterm infants significantly correlates with heart rate (HR) and HRV parameters, the lower the gestational age, the higher is mean HR for longer time and lower HRV. A decrease in HRV is linked to vulnerability to stress whereas an increase represents physical and mental adaptability [69, 70]. There is an association between maturation of the autonomic nervous system (ANS) of preterm infants with short- and long-term implications for social and emotional development, the manifestation of externalizing, internalizing and cognitive/academic problems and neuropsychiatric disorders in children [68, 71–75]. A decrease in the high-frequency domain of HRV during breastfeeding of preterm infants may imply an adaptive response to stimulation [76] (Brown, 2007). Limited evidence shows that vagally mediated HRV of 6–11 and 17-24-month-old infants (including premature infants from low SES background) was not associated with measures of development [77]. Further, newborns of depressive mothers had lower vagal tone than those of nonsymptomatic mothers [78]. Lower vagal tone was noted in 6-month old infants of depressed versus non-depressed mothers [79]. Postnatal maternal depression is associated with 14-month-old infant ANS functioning, that is a higher mean HR level and a trend toward lower vagal modulation [80]. Only a limited number of studies has provided contradictory findings regarding the association between inter-parental functioning with measures of ANS maturation early in life. Six-month-old infants in families with higher marital conflict showed lower’ baseline vagal tone, suggesting a decreased capacity for effective regulation [81]. In the meanwhile, high parent conflict was associated with a lesser degree of RSA withdrawal [82]. Further, interparental conflict avoidance demonstrated an association with 5-month-old infant’s baseline vagal tone [83].

*The relationship between autonomic nervous system and melatonin*. An internal circadian clock, which allows adaptation of physiological and behavioral functions tο the light-dark cycle, is coordinated by the paired suprachiasmatic nuclei (SCN), the central pacemaker. The pineal gland—which synthesizes and secretes melatonin—and SCN, interact with each other and constitute the key elements of the bio-clock. SCN relays photoperiodic information to the pineal gland via sympathetic nervous system. In connection to this information provided by the SCN, the pineal gland either up- or down-regulates the melatonin production. In addition to the SCN, there are peripheral clocks present in almost all other organs of the body, including the uterus and placenta. From the SCN, temporal information is transferred to peripheral circadian clocks via the autonomic nervous system and endocrine signals. The changing melatonin signal in turn impacts the function of the SCN given that melatonin receptors are expressed and densely populated in the SCN [for extended discussion see 84,85]. Though the timing of the appearance of endogenous SCN rhythmicity in humans is still lacking, there is evidence that by midgestation, the SCN neurogenesis and innervation by the hypothalamic tract is complete. Fetal circadian rhythms, coupled to the maternal rhythm, can be observed in utero from 30 weeks gestation but synchronization to the external environment occurs only after birth [86]. Melatonin in breast milk constitutes one important cue for this synchronization. In the meanwhile, in the event of preterm birth, the central autonomic nervous system is immature [87], the infant loses the temporal signals received by the mother prematurely and the SCN has not completely matured as SCN development continues throughout gestation and in the neonatal period after term birth [85].What is more, exposure to unpredictable patterns, chaotic and conflicting temporal cues in the NICU environment (such as noise, care-giving, medical interventions, orogastric feeding and mechanical ventilation) can impair the development of circadian rhythms with a potential to impact health and well-being throughout adult life [86].

### New knowledge to be acquired by this study

The proposed study is novel in the following ways: (1) The role of melatonin for preterm infant development is unclear [65]. Towards this direction, we aim to investigate the role of melatonin on infant development through a suspected multifactorial early developmental pathway combining psychosocial and biological factors; (2) The proposed study on determinants of premature infant development is longitudinal. Appropriate longitudinal studies for premature infants are scarce while the most prevalent studies on melatonin in human milk are cross-sectional. In order to fully recognize the difficulties of children born preterm, it is important to follow them individually, through several developmental stages [88, 89]. Longitudinal studies should be pursued in forthcoming years to deepen our understanding of the pathways leading to heightened risk for adverse developmental outcomes in preterm infants [90]; (3) This is an inter-disciplinary study. Focusing on psychosocial and physiological factors related to prematurity invites the Psychology—Medicine interdisciplinary collaboration because: a) premature infants possess behavioral characteristics and have neurological immaturity that may contribute in making them difficult interactive partners [91] and increase their health risks [69]; and b) a steady increase in the neonatal survival rates since the advent of modern intensive care for the preterm infant has been related to a growing concern for preterm infants’ developmental outcome and quality of life [92]. What is more, the dynamic nature of melatonin in human milk calls for continued interdisciplinary research [89]; (4) This is a multi-method study including physiological and hormone measures, one observational instrument and self-report validated questionnaires. Using multiple outcome measures in infant research is one way to increase rigor, and at the same time, enable us to more accurately interpret our data [93]; (5) Evidence on premature infant development is limited in Greece [94]. Melatonin in human milk has not been studied in Greece though there is relevant research in various other European countries [89]. To the best of our knowledge, the proposed study will be the first of this kind in Crete, Greece at a time after the financial crisis and in the course of the pandemic. This is important because the financial crisis of the last decade has increased the number of people living in extreme poverty in Greece and led to partial operation of mental health services [95–97]. What is more, the pandemic increased income inequalities [98] and affected adversely the mental health of Greeks [99, 100]. In Crete, poverty and mental health problems, especially for women, are prevalent and interconnected issues. Multiproblem families have been said to be on the rise [95]. Further, preterm births constitute a major public health issue in Greece [101]. Preterm birth rate has increased fourfold in the past two decades. These findings pose dramatic challenges for public health and emphasize the need for preventive interventions to be implemented [101].

### Scientific, social impact and implications on clinical practice

ANS activity, as an index of health risk in Medicine, has a critical role for maintaining cardiovascular and respiratory homeostasis, but is also intrinsically connected to higher brain systems involved in the emotional and psychological aspects of human life within Psychology [68, 75]. Also, for Medicine, melatonin appears a promising molecule to prevent brain insults associated with prematurity [102]. At the same time, for Psychology, melatonin is important for normal neurodevelopment. In the meanwhile, disruption to this cue may occur during admission of preterm neonates to the NICU [55, 86, 103]. Last, prematurity within DOHaD paradigm has been discussed mainly according to cortisol and HPA dysregulation [10] but this may be extended by investigating other hormonal and physiological factors, such as the combination of autonomic nervous system maturation and maternally-derived melatonin.

Perinatal mental health problems are a major public health concern globally [104]. Maternal perinatal mental health disorders have persistent effects on behavioural, physiological and immunological functioning throughout the lifespan and may even be evident across generations [105]. Prevention goals requires effective interventions that reach women at risk for, but prior to, the development of a depressive disorder. Early interventions improve sub-clinical symptomatology for at risk dyads at a crucial time of early postpartum period [106]. In addition, prematurity is considered a chronic and multigenerational condition [107]. In connection to this, potential deficits in infants born preterm may be amenable to early intervention. Early detection of developmental delays is a process of ongoing assessment and consideration of key perinatal events known to affect long-term outcomes [108]. Taken together, the findings of this study may highlight the need for future community-based prevention efforts and may convince policy makers and government to increase evidence-based interventions and family-focused care targeted on the promotion of perinatal mental health-care of mothers and preterm infants’ development from low SES. Designing evidence-based e-Health screening applications with the aim to improve universal access to developmental screening by bringing services into the homes of vulnerable populations [109] may take the form of targeted interventions for significant public health benefit.

Further, this study may extend our understanding about melatonin in human milk and may support the promotion of exclusive breastfeeding of premature neonates in the initial 6 months of infant’s life and maternal-child health by nurse professionals [89]. Fetal circadian rhythm synchronise to the external environment only after birth. One of the important cues for synchronization is exposure to melatonin in breast milk. This study may add knowledge to the important role of nighttime human milk which is featured by elevated melatonin concentrations with the aim to contribute in the strong rationale to evaluate strategies to protect the postnatal entrainment of circadian rhythms of premature neonates in the NICU [86] and to promote synchronization of composition and provision of expressed milk in the NICU setting (using circadian-matched human milk) [85, 89]. Recognizing and adding these considerations into breastfeeding recommendations may promote both infant and maternal well-being with optimal health outcomes [89]. This is important given: a) the impaired maturation of preterm infants’ circadian system and the tremendous mismatch with the uterine chronobiological environment that arises in the event of preterm birth [85]; and b) the disruption of circadian rhythm of melatonin secretion faced by women with threatened preterm labor [110].

## Materials and methods

### Aim, design and setting of the study

The main aim of this study is to investigate the association of certain psychosocial and biological factors across the first year of life of preterm infants’ development, with focus on low socio-economic status families. Thus, we plan to accomplish the following two objectives:

Objective 1: To explore the way psychosocial factors, such as maternal mental health, maternal perception of infant’s intersubjectivity and attachment, across the first year of preterm birth are related with infants’ emotional and cognitive development at 9 months (corrected age). Further, we will explore whether this association varies between infants from low and high SES. In addition, we will investigate whether other characteristics (family functioning, perceived social support and dyadic coping) may be related to these associations and if these characteristics can moderate for the risks posed by low socio-economic status,

Objective 2: To assess the way physiological factors, such as autonomic nervous system maturation, measured according to heart rate variability, is associated with premature infants’ emotional and cognitive development at 9 months (corrected age) and whether this association varies between low and high SES. Further, we will explore whether maternal derived-melatonin through breastfeeding intervenes this association.

Preterm neonates (<37 weeks) hospitalized in the Neonatology Department/NICU of the University Hospital of Heraklion and their mothers will participate in this longitudinal study.

According to the timeline of the study, participant recruitment and data collection, which started on the 10^th^ November 2023, are still in progress. It is difficult to estimate the period of completion of participant recruitment and data collection given that it is much dependent on: a) the number of preterm births, which are unpredictable events; b) the admission rate at the referent Hospital during the study period which cannot be predicted since in certain months 10–12 preterm neonates were born while in others there were no preterm births; c) the acceptance rate of new mothers, which at the moment ranges from 70–80% of approached mothers; and d) the percentage of new mothers who meet the inclusion criteria (see below). At the moment, participant recruitment is estimated to be completed not before the next two months and data collection is estimated to be completed by July or August 2025. The first results of the study are expected around March—April 2025.

### Sample size, inclusion and exclusion criteria

We aim to obtain a sample size of at least N = 100 mother-child pairs, to detect a 10-point difference on continuous outcomes with mean 100 and SD 15 (such as typical IQ index). Power calculations will be examined assuming a level of statistical significance of 5% and an 80% power. As mentioned above, the final number of subjects is much dependent on the admission rate at the referent Hospital during the study period.

#### Inclusion criteria

Inclusion criteria for mothers include the following: (1) parents are not divorced; (2) both parents are older than 20 years of age; (3) mother intends to breastfeed at least the first 28 days of neonate’s life. Inclusion criteria for neonates include prematurity (gestation age < 37weeks).

#### Exclusion criteria

Mothers will be excluded from the study if: (1) they suffer from a psychiatric illness; (2) they have issues with drug or substance abuse; (3) they are not biological parent or they are surrogate mother; and (4) they belong to a same-sex couple; (5) they do not intend to breastfeed; and (6) refusal of the parents to be included in the study. Infants will be excluded from the study if: (1) they have perinatal asphyxia; (2) they have neurological pathologies; (3) they experience malformation syndromes and major congenital malformations; (4) they have sensory deficits; (5) they present metabolic genetic disease; (6) they suffer from CNS infection. Participation in this study presupposes that both infants and mothers meet the inclusion and exclusion criteria.

### Measures

**Fig 1:**
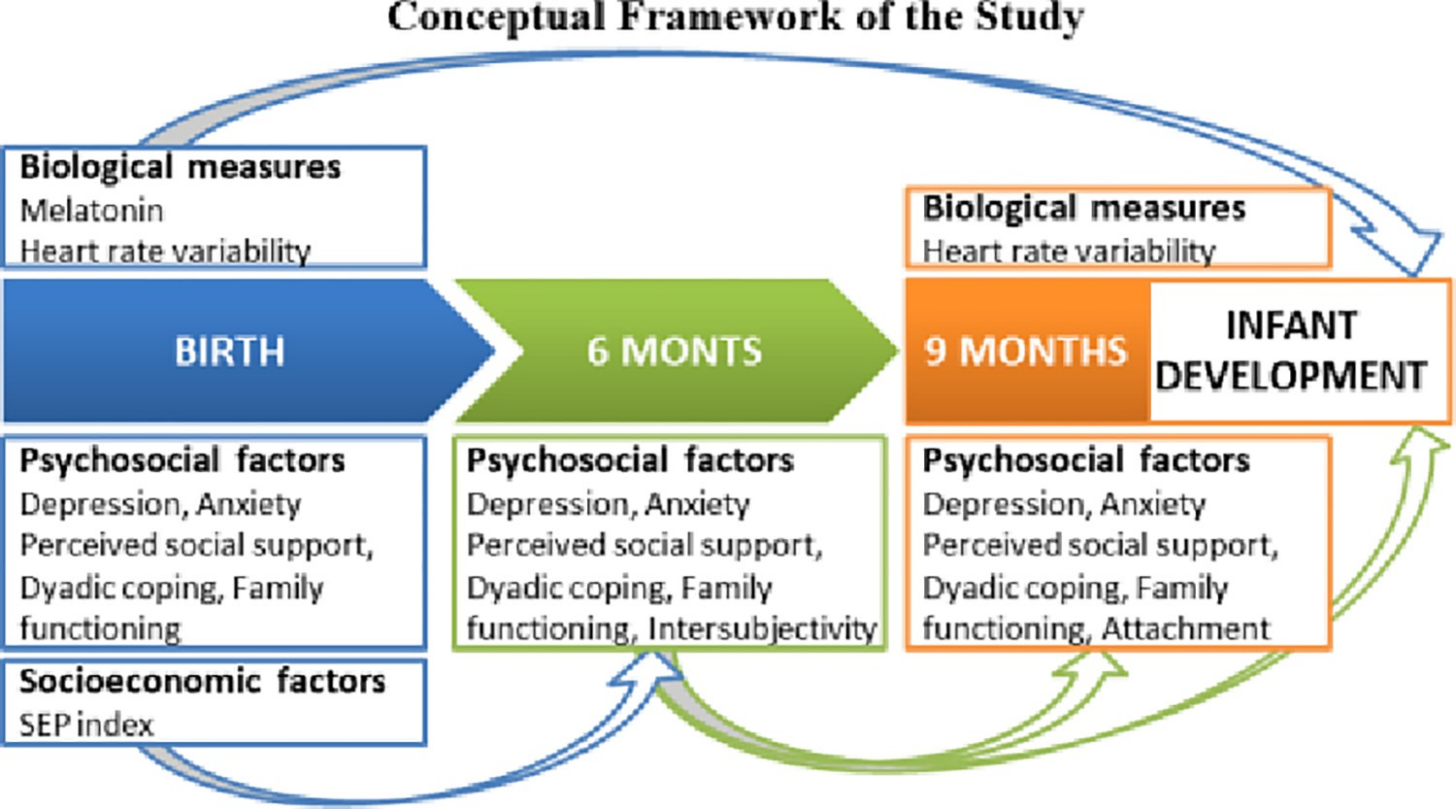
Conceptual framework of the study.

Fig 1 presents the conceptual framework of the study.

#### a. Socio-economic factors

At birth, we aim to assess various early-life stressors during pregnancy and early childhood. We will identify socio-demographic stressors with major consequences for health and development. These data will be used to evaluate how early-life stressors cluster or co-exist and how can be linked to infants’ developmental outcomes. We plan to collect many variables related to socio-economic status, including parental education, occupation, type of employment contract, family income, family size, child care, house property and size, and utilities. These data will be used to conduct a probabilistic linkage with the European Union Statistics on Income and Living Conditions (EUSILC) to obtain information for socio-economic status and social disadvantage in early life. Once internal and external data are linked, information will be used to derive a socio-economic position (SEP) index [111].

Furthermore, while the ethnic composition is changing, especially among younger generations, not much is known about the direct and indirect roles of ethnicity, migration and integration on health and disease from early life onwards. We plan to use data of birth of parents and children (ISO3 country codes), migrant history and status to create measures, such as “migration generation status” and “parents’ educational selectivity” [112].

#### b. Psychosocial factors

*Maternal depression*. At birth, the well-validated *Edinburgh Postnatal Depression Scale* [113] (EPDS) will be used to screen possible depressive symptoms in new mothers. EPDS is a 10-item self-rating scale designed to particularly target populations during both the antenatal and post-natal period. Each item is scored from 0 to 3. The EPDS has been validated in a variety settings and community samples with the majority of studies focusing on the 6–8 week postpartum period [114]. The EPDS has been validated in Greek [115]. At 6 and at 9 months after birth, *the Beck Depression Inventory-II* (BDI-II) [116] will be used to screen maternal symptoms of depression experienced in the past week. The BDI-II is a revised 21-item self-report test with 4 response options per item. The BDI-II has been validated in Greek [117].

*Maternal anxiety*. At birth and at 6 and 9 months, *the Spielberger State-Trait Anxiety Inventory for Adults* [118] (STAI) will be used to measure maternal anxiety. The STAI is a 40-item self-report measure with a 4-point Likert-type scale for each item. It has two scales: State anxiety (how one feels at the moment, 20 items) and Trait anxiety (how one generally feels, 20 items). The STAI has been validated in Greek [119].

*Family functioning*. At birth and at 6 and 9 months, the *Family Adaptability and Cohesion Evaluation Scales IV Package* [120, 121] (FACES IV) will be used to assess perceived family functioning. The FACES IV Package contains the six scales from FACES IV (42 items) [two balanced scales (balanced cohesion, balanced flexibility) and the four unbalanced scales (Disengaged and Enmeshed for cohesion and Rigid and Chaotic for flexibility)], the Family Communication Scale (FCS) and the Family Satisfaction Scale (FSS) (62 items in total). The FACES has been validated in Greek [122].

*Perceived social support*. At birth and at 6 and 9 months, the *Multidimensional Scale of Perceived Social Support* [123] (MSPSS) will be used to assess the perception of social support mothers receive from three sources, each corresponding to a subscale: family, friends, and significant other. The MSPP is a 12-item reliable and valid self-rating scale as each of the three subscales consists of four items, rated on a seven-point Likert type scale. The MSPSS has been validated in Greek [124].

*Dyadic coping*. At birth and at 6 and 9 months, the *Dyadic Coping Inventory* [125] (DCI) will be used to measure dyadic coping behaviors. The DCI is a 37-item reliable and valid instrument with 10 subscales. Items are rated on a 5-point scale. The DCI has been validated in Greek [126].

*Intersubjectivity*. At 6 months after birth, *t*he *Maternal Perception of Infant’s Intersubjectivity Questionnaire* [47] (MPIIQ) will be used to assess maternal perception of the infant’s intersubjectivity. The MPIIQ is a 22-item self-report questionnaire with good psychometric properties. Items are grouped in three factors: Factor 1 is related to maternal perception about the infant’s competence at the interaction with the mother, Factor 2 refers to maternal perception of infants’ behaviors that express emotional states and Factor 3 is related to maternal perceptions about the infants’ competence to express their own initiatives.

*Attachment*. At 9 months after birth, *t*he *Maternal Postnatal Attachment Scale* [127] (MPAS) will be used to assess mother’s subjective feelings of attachment to her infant via maternal self-reported feelings, cognitions and behaviours in relation to their infant. The MPAS is a 19-item self-report questionnaire and each item has a 2-, 4- or 5-point response option. The MPAS includes three subscales: quality of attachment (9 items), absence of hostility toward the infant (5 items) and pleasure in the interaction (5 items).

#### c. Biological measurements

*Melatonin*. Breastmilk melatonin concentrations: Mothers of preterm neonates will be asked to collect 5-10ml of nighttime breastmilk with the use of an electrical pump between 01:00–05:00 a.m. at three specific time points: 3^rd^-5^th^ day (colostrum), 10^th^-14^th^ day (transitional milk) and 20^th^-28^th^ day (mature milk). The milk will be collected in a sterile container, transported to the hospital at 4^ο^C, and frozen immediately at -80°C until analysis. Melatonin concentrations of nighttime milk are elevated across all milk phases and they are approximately ten times compared to that in the daytime. In preterm breastmilk, the melatonin concentration presents a circadian rhythm with the acrophase at around 03:00 a.m. though peak melatonin production differs among individuals [55, 89]. The concentration of melatonin is significantly higher in colostrum [128]. Melatonin has been found in colostrum with the comparable concentration as it in plasma [129]. Most studies did not identify associations between melatonin in human milk and gestational age [54].

Blood sample melatonin concentrations: An umbilical cord blood sample of 1-2ml will be collected at every preterm delivery or caesarean section, in order to measure the melatonin level at the time of birth. Blood samples from the preterm neonate will be collected as follows: a. for premature neonates > 33 weeks, 3 samples will be collected 1) the first neonate blood sample (scheduled blood test examination) when the neonates enters the NICU (taking into account practical difficulties in relation to the presence of a research team member in the delivery room), or the first blood sample will be that of the umbilical cord, irrespectively of gestation age, 2) 4^th^-7^th^ day of life and 3) 10^th^-14^th^ day; b.for premature neonates <33 weeks, 4 samples will be collected (the first three same as for premature >33 weeks and the fourth sample at a date that will coincide with a gestational age of 35–36 weeks).The blood will be collected from the clinic’s medical staff, along other scheduled blood tests, so that no extra interventions are performed on the neonates for the purpose of this study. The time of blood sampling will be between 8:00–10:00 am and the amount of plasma needed is estimated at 500μL. Umbilical cord blood and neonate blood samples will be centrifuged at 3000 g for 5 min, and then the plasma will be separated and frozen at −80°C until analysis.

The melatonin levels in breast milk and in blood samples will be determined by Enzyme-linked Immunoassay (ELISA) according to the manufacturer’s instructions.

Melatonin is present in quite high levels in the umbilical cord blood, which may be at least in part, of maternal origin [130]. Clear differences have been reported between day and night values in melatonin concentration in the umbilical artery and vein as measured through blood sample at birth. Further, some authors have described changes in the production and rhythmic secretion of melatonin during the fetal and neonatal period [131]. Over the course of development, extrapineal melatonin production in the placenta rises until birth. Melatonin levels in the mother increase from 32 weeks of gestation. The circadian rhythm of the fetal heart rate is evident by 30 weeks of gestation, and by 32 weeks, different types of sleep can be distinguished [56]. Taken together, these may imply variations of amounts of melatonin in the umbilical cord blood according to gestational age.

Preterm infants have no or minimal circulating plasma melatonin [132]. In particular, plasma melatonin concentrations, measured at birth and on Day 3 were below detectable levels (7 pg/mL) in 78% and 81%, respectively, of infants born before 34 GW compared to 57% and 34%, respectively, of infants born after 34 GW. The distribution of plasma melatonin concentrations was found to be correlated with gestational age at both time-points [102].

On this ground, careful consideration will be taken for the sensitivity and specificity of assays used for melatonin analysis. In a different case, it may not be possible to measure aMT6s (melatonin primary metabolite 6-sulfatoxymelatonin) in the premature neonates’ blood due to limitations in the amount of blood that can ethically be collected for this vulnerable population group according to international guidelines [132]. Additionally, the production and secretion of colostrum are not abundant making sample collection more difficult [133].

*Heart rate variability*. Neonates’ / infants’ and maternal HRV measurements will be obtained at 2 successive time intervals (neonatal period and at a corrected age of 9 months after the birth). HRV measurements of short-term variability may provide important information about the maturation of the ANS in newborns [134, 135]. HRV parameters in the time-domain (SDNN, HRm, HRstd, RMSSD, NN50, pNN50, HRV triangular index), the frequency-domain (Total power, LF, HF, LF/HF, LF_norm_, HF_norm_) as well as non-linear indices (ApEn, DFA α_1_, α_2_) will be analyzed [136, 137] in order to identify the cardiac activity and SNS/PNS activation patterns. HRV measurements will be carried out through SEER 1000, ECG Recorder, General Electric (Version 1.0, 2067634–077 Revision F).

Timeline and conditions of neonates’ HRV measurements: Postnatal age at the time of HRV testing may contribute to differences in early autonomic tone since there is evidence for a postnatal *transitional period of maturation of the ANS*, *cardiovascular*, *and respiratory systems*. This transitional period extends for a few days beyond delivery resulting in a maturational increase in HRV metrics when ANS tone is evaluated both within a few hours of birth and at three to four days of age. Thus, it is possible that there may be immediate, but limited effects on infant HRV according to both the circumstances of birth and the timing of testing [68].

For preterm neonates with a low level of medical morbidity, the duration of extrauterine development does not significantly impact ANS developmental trajectory from birth to NICU discharge [138]. No increase in the parasympathetic measures were evidenced for premature neonates born from 28 to 32 weeks when measured throughout a follow up period 32–35 weeks postmenstrual age (PMA) [139]. Meanwhile, a significant effect of postnatal age [comparing 3^rd^ to 4^th^ postnatal day vs early after birth (1^st^-2^nd^ hour after birth)] revealed that the mean RR interval was significantly longer (increases in HRV) on the third to fourth postnatal day of term neonates, regardless the mode of delivery. This indicates cardiac autonomic maturation within the third to fourth postnatal day in spontaneously delivered and surgically delivered neonates [140]. In connection to this, within 14 h after birth of term (male) neonates (comparison between 2h and 14h after birth, that is 12h after the first measurement) born of elective cesarean delivery, the mean of the iRRs increased as well as parasympathetic indices. Previous studies using short recording methods had documented that in a few days after birth (from the 2nd to the 4th day after birth), there is an increase in the HRV indices in healthy term infants. During the first three days of life, different authors verified a gradual increase of the parasympathetic portion and a simultaneous reduction of sympathetic activity. Thus, studies suggest that HRV increases gradually during the first three days of life [141].

Based on the above mentioned recent evident, three measurements for neonates’ HRV measurements will be carried out:

- The first measurement will be performed within 24 hours after birth (in the supine position in the incubator),
- The second measurement will be performed on the third to fourth postnatal day after birth,
- Only for premature neonates born before 35 gestation weeks, a third measurement will be carried out at around 35–36 weeks PMA.

Ten-minute time length HRV measurements, which provide a good compromise with minimal error for all features estimations, will be obtained 30 minutes to 1 hour after a morning-time feeding period (between 8.00 and 12.00 a.m.) to minimize its effect on HRV [140], without painful or stressful procedures for at least 6 hours. All of the neonates will be in supine position during the recordings. Recordings will be delayed by 48 hours in cases of unstable/unpredicted acute pathology, or administration of drugs with cardiac effects in the 7 days preceding the recording [142].

Timeline and conditions of maternal HRV measurements: Sympathetic nervous system activity increases while parasympathetic activity normatively decreases across pregnancy [143]. However, the extent and the timing of cardiac recovery have been a subject of debate. At 49 days prior to birth there is a reversal of HRV indices with a steady increase in daily HRV that continued in the postpartum period [144]. Meanwhile, heart rate (HR) was reported to return slowly to baseline levels by 2–6 weeks postpartum in some studies. Pulse rate declined shortly after delivery and reached a relatively low level but it did not return to normal within 6 weeks [145]. Conversely, it increased from the 7th week to the 11th week postpartum. Meanwhile, there is some evidence to suggest that vagally-mediated HRV returns to pre-pregnant levels within three months [146]. This is consistent with evidence showing that HRV parameters return to normal within three months after delivery [147]. In one report, the recovery period was around 20 weeks. In other studies, a continued decrease in cardiac output was observed to last over the next 24 weeks [145]. Further, vagally-mediated HRV increased between 3^rd^ trimester and 4–6 weeks postpartum [143]. Other studies indicated that cardiac activities might not return to original levels even after one year. However, there has also been concern that the changes in cardiac function associated with pregnancy might not ultimately return to pre-pregnancy levels [145].

What is more, labor is associated with significant physiological changes. In the course of labor, there are continuous adjustments of cardiac autonomic reflexes by alternate activations of the sympathetic and parasympathetic nervous systems [148]. A relationship between autonomic nervous system indexed by HRV and the pain response has been confirmed [149]. Further, labor pain intensity is known to predict persistent postpartum pain. After the delivery, uterine contraction pain is common within 48 hours of delivery and postpartum pain may last between four weeks to three months [84]. Taken together, maternal postpartum pain experience may persist for days or weeks after birth and may affect maternal HRV measurements in the course of the first days after delivery.

The above literature review provides evidence that possibly maternal HRV values do not recover before the 2^nd^ week postpartum and maternal postpartum pain experience may persist for days or weeks after birth and may affect maternal HRV measurements in the course of the first days after delivery. On this ground:

a) Assessment of maternal postpartum pain according to a numeric rating scale will precede maternal HRV measurements,

b) Maternal HRV measurements will be carried out between the 3^rd^ and 6^th^ day postpartum. Maternal HRV measurements will be continuously measured for 5 minutes noninvasively at each time period. All participants will be in a sitting position [141]. Maternal HRV will be measured in the morning between 11.00 a.m. and 2.00 p.m. and 1–2 hours after a morning time-feeding hour.

*Nutritional intake*. Foods containing melatonin or promoting the synthesis of it by impacting the availability of tryptophan (an essential dietary amino acid for melatonin synthesis) as well as those containing vitamins and minerals (co-factors and activators in the synthesis of melatonin) may affect the levels of maternal melatonin [150]. On this ground, some nutritional factors that may affect maternal levels of melatonin concentrations will be assessed.

#### d. Assessment of premature infants’ developmen

At 9 months corrected age, the development of infants will be assessed at hospital by the administration of the *Bayley Scales of Infant and Toddler Development*, *3*^*rd*^ *Edition* [151] which is a standardized, diagnostic developmental assessment instrument for infants and young children between one and 42 months. Bayley-III is comprised of 3 scales: cognitive scale, language scale (receptive and expressive communication) and motor scale (fine and gross motor).

### Ethical considerations and declarations

Data will be collected via electronic questionnaires to be directly aggregated to databases and subjected to quality control procedures, including point of entry and logical validation. All data will be de-identified and no individual’s name or other personal information will be associated with any published or unpublished report of this study. Data will be stored on a secure server in UOC facilities. All data confidentiality and security provisions will be strictly maintained to guarantee that all personal data collection and processing will be carried out in accordance with the GDPR (EU 2016/679) and national legislation. Project personnel will be trained in the importance of confidentiality of individual records and required to sign a confidentiality agreement. All participants will also sign a written-consent form. Given that this study includes minors, we will obtain written consent for their participation from their mothers. The study subjects may always change their opinion in the course of the study for any reason at any time.

The protocol of this study has been approved by the Research Ethics Committee of the University of Crete (numbers and dates of decision: 103/22.09.2023, 158/15.12.2023 and 38/15.02.2024) and by the Scientific Council, according to the positive recommendation of the Working Group of the Ethics Committee, and the Board of Directors of the General University Hospital of Heraklion (reference number: 26636/2.10.2023 and 35546/23.10.2023).

### Statistical analysis plan

Initial exploratory analysis will be used to understand variable distributions and to identify transformations that satisfy modeling assumptions (e.g., log-transformation of right skewed variables), to understand correlations, and to identify extreme observations. Throughout the modeling process, we will evaluate and modify models to ensure that inferences are not unduly influenced by erroneous modeling assumptions and/or influential observations. Generalized Additive Models (GAMs) will be employed in order to determine the shape of the associations.

By applying robust linear regression modeling techniques on continuous outcomes and generalized linear modeling techniques for binary outcomes, adjusting for potential confounding factors, we aim to identify the psychosocial and physiological risk factors associated to adverse preterm infants’ development at 9 months corrected age, between low and high socio-economic status (SES) families.

We shall also leverage our data to deploy statistical models testing the factors that mediate (medical history, anthropometry and biomarker data) and moderate (lifestyle and social factors) the associations we may observe. We plan to evaluate the synergistic role of combined exposures to these factors through supervised shrinkage methods. We will also use pathway approaches (Structural Equation Modeling–SEM) to study the pathways connecting exposures under study to psychosocial phenotypes.

In order to improve the statistical power of the study, most outcomes will be analysed continuously rather than categorically.

## Discussion

### Limitations

Longitudinal studies with larger sample size and longer follow up comparing relevant psychosocial and biological factors and the development of both preterm and full-term neonates are necessary. Melatonin in human milk needs to be measured according to breastfeeding duration given the reduction of melatonin concentration at the end of feeding compared to the pre-breastfeeding period [152]. Future research needs to address respective issues in relation to women’s way of birth (vaginal delivery vs cesarean section) given the relevant contradictory evidence [89]. Given the disruption of normal entrainment cues in the NICU (86), the way unpredictable patterns and the chaotic ad conflicting temporal cues in the environment of NICU–especially lighting conditions—may mediate the relationship between melatonin concentration in breast milk and infant development needs further investigation. Longitudinal studies that follow women from the beginning of pregnancy are needed since rhythms in the prenatal stage are influenced by maternal signals and prenatal development likely sets the stage for healthy post-natal development. Further, cortisol measurements need to be added since the fetal circadian rhythm is synchronized to maternal cortisol and melatonin levels, rather than to the external light/dark cycle per se. Pregnant women experience changes in circadian regulated plasma cortisol and melatonin concentration [153].

### Dissemination plans

Dissemination activities will target scientific audiences, health care stakeholders and community audiences. Dissemination plans include: a) Construction and maintenance of project website which will be maintained for at least 5 years after completion of the project; b) Preparation and submission of two manuscripts for international peer-review journals; c) Preparation and submission of two presentations for national and international conferences; and d) Preparation of two presentations for community audiences.

### Risks and contingency plan

In the course of this study, a number of risks may be needed to be dealt with. In particular, in order to:

1. avoid delay of study protocol approvals by Ethics Committees, we applied and received approvals immediately upon notification of acceptance of the project by the Hellenic Foundation of Research and Innovation,
2. avoid difficulties in subject recruitment, research assistants have been trained to approach new mothers and to develop a personal communication with them,
3. ensure representativity of the sample, an initial sampling plan has defined desired sample characteristics (distribution per SES, sex etc). Recruited sample characteristics are monitored and recruitment procedure are informed accordingly,
4. deal with mother’s negligence to complete the entire battery of psychological assessment, research assistants monitor the completeness of answers in the battery of assessments and inform mothers accordingly,
5. avoid drop out of participants, personal invitations are sent to participants before the follow up. At follow up: a) mothers constantly will receive feedback concerning measurements of their infants, or their own biological samples; b) researchers will discuss with new mothers issues related to their infant development and will receive informative brochures. Research assistants, are trained to develop a personal communication with the mothers and to respond promptly to their questions, or to issues of concern for their infant development.

## Supporting information

S1 File Initial detailed study protocol submitted to Ethics Committee of the University of Crete in Greek. (PDF)

S2 File Initial detailed study protocol submitted to Ethics Committee of the University of Crete in English. (PDF)

S3 File Updated measurements of biological samples with certain modifications submitted to Ethics Committee of the University of Crete in Greek. (PDF)

S4 File Updated measurements of biological samples with certain modifications submitted to Ethics Committee of the University of Crete in English. (PDF)
