# Supplementary material for: The development of preterm infants from low socio-economic status families: The combined effects of melatonin, autonomic nervous system maturation and psychosocial factors (ProMote): A study protocol
Source: PLoS One. 2025 Jan 10;20(1):e0316520. doi: 10.1371/journal.pone.0316520 (PMC11723634; doi:10.1371/journal.pone.0316520)
Supplement: S1 File — (PDF) [file pone.0316520.s001.pdf]

ΕΡΕΥΝΗΤΙΚΟ ΠΡΩΤΟΚΟΛΛΟ
(ΚΑ 11455)

ΤΙΤΛΟΣ ΕΡΕΥΝΗΤΙΚΟΥ ΕΡΓΟΥ

Η Ανάπτυξη των Πρόωρων Βρεφών Οικογενειών Χαμηλού Κοινωνικο-Οικονομικού Επιπέδου: Οι Συνδυαστικές Επιδράσεις της Μελατονίνης, της Ωρίμανσης του Αυτόνομου Νευρικού Συστήματος και Ψυχοκοινωνικών Παραγόντων (ProMote)

The Development of Preterm Infants from Low Socio-Economic Status Families: The Combined Effects of Melatonin, Autonomic Nervous System Maturation and Psychosocial Factors (ProMote)

ΧΡΗΜΑΤΟΔΟΤΗΣΗ ΤΟΥ ΕΡΕΥΝΗΤΙΚΟΥ ΕΡΓΟΥ

Το ερευνητικό έργο με τίτλο: “Η Ανάπτυξη των Πρόωρων Βρεφών Οικογενειών Χαμηλού Κοινωνικο-Οικονομικού Επιπέδου: Οι Συνδυαστικές Επιδράσεις της Μελατονίνης, της Ωρίμανσης του Αυτόνομου Νευρικού Συστήματος και Ψυχοκοινωνικών Παραγόντων (ProMote)”, υλοποιείται στο πλαίσιο της δράσης του ΕΛ.ΙΔ.Ε.Κ. «Χρηματοδότηση της Βασικής Έρευνας (Οριζόντια υποστήριξη όλων των Επιστημών)» του Εθνικού Σχεδίου Ανάκαμψης και Ανθεκτικότητας «Ελλάδα 2.0» με τη χρηματοδότηση της Ευρωπαϊκής Ένωσης – Next Generation EU (Αριθμός Έργου ΕΛ.ΙΔ.Ε.Κ.: 15730).

The research project entitled: “The Development of Preterm Infants from Low Socio-Economic Status Families: The Combined Effects of Melatonin, Autonomic Nervous System Maturation and Psychosocial Factors (ProMote)”, is implemented in the framework of H.F.R.I call “Basic research Financing (Horizontal support of all Sciences)” under the National Recovery and Resilience Plan “Greece 2.0” funded by the European Union –NextGeneration EU (H.F.R.I. Project Number: 15730).

ΕΡΕΥΝΗΤΙΚΗ ΟΜΑΔΑ

Θεανώ Κοκκινάκη (Επιστημονικά Υπεύθυνη), Καθηγήτρια Αναπτυξιακής Ψυχολογίας, Τμήμα Ψυχολογίας, Εργαστήριο Εφαρμοσμένης Ψυχολογίας, Μονάδα για την Ανάπτυξη και την Εκπαίδευση του Παιδιού, Πανεπιστήμιο Κρήτης, Πανεπιστημιούπολη Γάλλου, Ρέθυμνο 74 150

Ελευθερία Χατζηδάκη, Επίκουρη Καθηγήτρια Νεογνολογίας Ιατρικής Σχολής Πανεπιστημίου Κρήτης, Διευθύντρια Νεογνολογικής Κλινικής και Μονάδας Εντατικής Νοσηλείας Νεογνών, Πανεπιστημιακού Γενικού Νοσοκομείου Ηρακλείου

Αριστείδης Τσατσάκης, Καθηγητής Τοξικολογίας, Ιατρική Σχολή, Πανεπιστήμιο Κρήτης, Ιατρική Σχολή, Πανεπιστήμιο Κρήτης

Εμμανουήλ Τζατζαράκης, Αναπληρωτής Καθηγητής Τοξικολογίας, Ιατρική Σχολή, Πανεπιστήμιο Κρήτης, Ιατρική Σχολή, Πανεπιστήμιο Κρήτης

Ελένη Βακωνάκη, Βιολόγος, Ερευνήτρια, Εργαστήριο Τοξικολογίας, Ιατρική Σχολή,

Πανεπιστήμιο Κρήτης

Θεανώ Ρουμελιωτάκη, Στατιστικός, Κλινική Προληπτικής Ιατρικής και Διατροφής, Τομέας Κοινωνικής Ιατρικής, Ιατρική Σχολή, Πανεπιστήμιο Κρήτης

Γιώργος Γιαννακάκης, Ηλεκτρολόγος Μηχανικός και Μηχανικός Υπολογιστών, Ινστιτούτο Πληροφορικής, Ίδρυμα Τεχνολογίας και Έρευνας.

Νικολίνα-Χίλντα Αναγνωστάτου, Παιδίατρος-Νεογνολόγος, Υποψήφια Διδάκτορας Ιατρικής Σχολής Πανεπιστημίου Κρήτης, Διευθύντρια ΕΣΥ Νεογνολογικής Κλινικής και Μονάδας Εντατικής Νοσηλείας Νεογνών του Πανεπιστημιακού Γενικού Νοσοκομείου Ηρακλείου

Μετά την αποχώρησή για επαγγελματικούς λόγους **της Ψυχολόγου** Μ.Φ. η ίδια θα αντικατασταθεί από Ψυχολόγο (PhD) που θα ενταχθεί στην ομάδα κατόπιν σχετικής εισήγησης της ΕΥ προς τον Ειδικό Λογαριασμό Κονδυλίων Έρευνας του Πανεπιστημίου Κρήτης.

Τεχνικός βοηθός (μη ονοματισμένος, θα ενταχθεί στην ομάδα κατόπιν πρόσκλησης εκδήλωσης ενδιαφέροντος (μέσω του Ειδικού Λογαριασμού Κονδυλίων Έρευνας του Πανεπιστημίου Κρήτης)

ΟΙ ΦΟΡΕΙΣ ΠΟΥ ΕΜΠΛΕΚΟΝΤΑΙ ΣΤΟ ΠΡΟΤΕΙΝΟΜΕΝΟ ΕΡΓΟ

Το προτεινόμενο έργο θα υλοποιηθεί μέσω της συνεργασίας του **Τμήματος Ψυχολογίας του Πανεπιστημίου Κρήτης, του Πανεπιστημιακού Γενικού Νοσοκομείου Ηρακλείου και του Ιδρύματος Τεχνολογίας και Έρευνας**.

Πιο συγκεκριμένα, η επιστημονικά υπεύθυνη του έργου είναι η κα **Θεανώ Κοκκινάκη**, Καθηγήτρια Αναπτυξιακής Ψυχολογίας του Τμήματος Ψυχολογίας του Πανεπιστημίου Κρήτης. Από το Πανεπιστημιακό Γενικό Νοσοκομείου Ηρακλείου, και ειδικότερα από τη Νεογνολογική Κλινική του ΠαΓΝΗ, προέρχονται τα μέλη της ερευνητικής ομάδας, κα **Ελευθερία Χατζηδάκη,** Επίκουρη Καθηγήτρια Νεογνολογίας Ιατρικής Σχολής Πανεπιστημίου Κρήτης, Διευθύντρια Νεογνολογικής Κλινικής και Μονάδας Εντατικής Νοσηλείας Νεογνών, Πανεπιστημιακού Γενικού Νοσοκομείου Ηρακλείου και η κα **Νικόλ Αναγνωστάτου**, Παιδίατρος-Νεογνολόγος, Υποψήφια Διδάκτορας Ιατρικής Σχολής Πανεπιστημίου Κρήτης, Διευθύντρια ΕΣΥ Νεογνολογικής Κλινικής και Μονάδας Εντατικής Νοσηλείας Νεογνών του Πανεπιστημιακού Γενικού Νοσοκομείου Ηρακλείου. Από το Ίδρυμα Τεχνολογίας και Έρευνας προέρχεται ο κος **Γιώργος Γιαννακάκης**, Ηλεκτρολόγος Μηχανικός και Μηχανικός Υπολογιστών, Ινστιτούτο Πληροφορικής του ΙΤΕ. Επιπλέον, από την Ιατρική Σχολή του Πανεπιστημίου Κρήτης προέρχονται οι κ.κ. **Αριστείδης Τσατσάκης**, Καθηγητής Τοξικολογίας, κος **Εμμανουήλ Τζατζαράκης,** Αναπληρωτής Καθηγητής Τοξικολογίας καθώς και η κα **Ελένη Βακωνάκη,** Βιολόγος, Ερευνήτρια, Εργαστήριο Τοξικολογίας της Ιατρικής Σχολής και η κα **Θεανώ Ρουμελιωτάκη**, Στατιστικός, Κλινική Προληπτικής Ιατρικής και Διατροφής Τομέας Κοινωνικής Ιατρικής, Ιατρική Σχολή, Πανεπιστήμιο Κρήτης.

ΕΙΣΑΓΩΓΗ

Η μελέτη των ψυχοκοινωνικών παραγόντων και των παραγόντων που σχετίζονται με τη φυσιολογία των πρόωρων βρεφών αποτελεί μια πρόκληση για τη διεπιστημονική συνεργασία μεταξύ των επιστημών της Ψυχολογίας και της Ιατρικής επειδή: α) τα πρόωρα βρέφη χαρακτηρίζονται από συμπεριφορικές ιδιαιτερότητες και νευρολογική ανωριμότητα, στοιχεία

τα οποία συνεισφέρουν στις δυσκολίες τους να συμμετάσχουν στην διαπροσωπική επικοινωνία (Singer, 2003) και αυξάνουν τους κινδύνους για την υγεία τους (Javorka, 2017); και β) μια σταθερή αύξηση στα επίπεδα νεογνικής επιβίωσης έχει συσχετιστεί με ένα αυξανόμενο ενδιαφέρον για τα αναπτυξιακά αποτελέσματα των πρόωρων βρεφών και την ποιότητα της ζωής τους (Forcada-Guex, 2006).

Κατά την περιγεννητική περίοδο οι γυναίκες που προέρχονται από χαμηλό κοινωνικο-οικονομικό επίπεδο (στο εξής κοκ) επηρεάζονται δυσανάλογα από τον πρόωρο τοκετό, την κατάθλιψη, το άγχος και από έλλειψη πρόσβασης στη φροντίδα ψυχικής υγείας. Οι συνδυαστικές επιδράσεις των παραπάνω σχετίζονται με την οικονομική και κοινωνική δυσχέρεια η οποία επηρεάζει τα άτομα, τις οικογένειες και την κοινωνία στο σύνολό της, επεκτείνοντας με τον τρόπο αυτό τον κύκλο της φτώχειας και των ανισοτήτων στα θέματα φροντίδας της υγείας (Prom, 2022). Οι βιολογικοί, ψυχολογικοί και κοινωνιολογικοί κίνδυνοι αλληλεπιδρούν με τρόπο που απειλεί την ικανότητα της οικογένειας χαμηλού κοκ επιπέδου να ανταποκριθεί στην ικανοποίηση των φυσικών, κοινωνικών και συναισθηματικών αναγκών των μελών της (Maurer & Smith, 2013). Το χαμηλό κοκ επιπέδου αποτελεί ένα από τους πιο καθοριστικούς παράγοντες που συσχετίζονται με την περιορισμένη νευροανάπτυξη των πρόωρων βρεφών. Τα υψηλότερα ποσοστά βιολογικών κινδύνων των πρόωρων βρεφών χαμηλού κοκ επιπέδου τα θέτουν σε διπλό κίνδυνο για χαμηλά νευροαναπτυξιακά αποτελέσματα (Panceri, 2020; Wong & Edwards, 2013). Παρά το γεγονός ότι οι καθυστερήσεις στην ανάπτυξη των πρόωρων βρεφών και εκείνων χαμηλού κοκ επιπέδου είναι καλά τεκμηριωμένες, υπάρχουν εξαιρετικά περιορισμένες ενδείξεις για την πρώιμη μεταγεννητική ανάπτυξη των πρόωρων βρεφών που εκτίθενται τόσο σε βιολογικούς όσο και σε περιβαλλοντικούς παράγοντες κινδύνου (Gonzalez-Gomez, 2019). Είναι πιθανόν οι αρνητικές επιδράσεις της φτώχειας να ξεκινούν κατά τα πρώιμα στάδια της ανάπτυξης, πιθανόν προγεννητικά (Blount, 2021; Hurt, 2017; Hosokawa & Katsura, 2018; Maurer & Smith, 2013).

Ο **βασικός σκοπός της προτεινόμενης διαχρονικής μελέτης** είναι η διερεύνηση της σχέσης συγκεκριμένων ψυχοκοινωνικών (μεταγεννητική κατάθλιψη, οικογενειακή λειτουργικότητα, κοινωνική υποστήριξη, μητρική αντίληψη για τη διυποκειμενικότητα και δεσμός) και βιολογικών παραγόντων/παραγόντων φυσιολογίας (μελατονίνη/μεταβλητότητα του καρδιακού ρυθμού) κατά τη διάρκεια του πρώτου έτους ζωής των πρόωρων βρεφών, εστιάζοντας στις οικογένειες χαμηλού κοκ επιπέδου, καθώς και οι επιπτώσεις της παραπάνω σχέσης στην ανάπτυξη των πρόωρων βρεφών. Εξαιτίας των περιορισμένων σχετικών ερευνών και των αντιφατικών ευρημάτων, δεν ήταν εφικτή η διατύπωση υποθέσεων.Οι δύο επιμέρους στόχοι της μελέτης είναι οι εξής:

Στόχος 1: Η διερεύνηση του τρόπου με τον οποίο συγκεκριμένοι ψυχοκοινωνικοί παράγοντες, όπως η μητρική ψυχική υγεία, η μητρική αντίληψη της βρεφικής διυποκειμενικότητας και του δεσμού, κατά τη διάρκεια του πρώτου έτους μετά τον πρόωρο τοκετό σχετίζονται με τη συναισθηματική και γνωστική ανάπτυξη των βρεφών στους 9 μήνες (διορθωμένη ηλικία). Επιπλέον, θα διερευνήσουμε εάν η παραπάνω σχέση διαφοροποιείται μεταξύ των πρόωρων βρεφών που προέρχονται από χαμηλό και μέσο/υψηλό κοινωνικοοικονομικό επίπεδο. Επιπρόσθετα, θα διερευνήσουμε εάν ψυχοκοινωνικά χαρακτηριστικά (όπως η οικογενειακή λειτουργικότητα, η αντιλαμβανόμενη κοινωνική υποστήριξη και η δυαδική αντιμετώπιση του στρες) μπορεί να συσχετίζονται με τα παραπάνω και εάν τα χαρακτηριστικά μπορεί να μετριάζουν τους κινδύνους που τίθενται από το χαμηλό κοινωνικο-οικονομικό επίπεδο.

Στόχος 2: Η αξιολόγηση του τρόπου με τον οποίο παράγοντες φυσιολογίας, όπως η ωρίμανση του αυτόνομου νευρικού συστήματος, όπως αξιολογείται από τη μεταβλητότητα του καρδιακού ρυθμού, συσχετίζεται με την γνωστική και συναισθηματική ανάπτυξη των βρεφών στους 9 μήνες (διορθωμένη ηλικία) και εάν η σχέση αυτή διαφοροποιείται μεταξύ των βρεφών από χαμηλό και μέσο/υψηλό κοκ επίπεδο. Επιπλέον, θα διερευνήσουμε εάν η

μητρική μελατονίνη μέσω του μητρικού γάλακτος διαμεσολαβεί στην παραπάνω σχέση.

ΘΕΩΡΗΤΙΚΟ ΥΠΟΒΑΘΡΟ ΤΟΥ ΠΡΟΤΕΙΝΟΜΕΝΟΥ ΕΡΕΥΝΗΤΙΚΟΥ ΕΡΓΟΥ

Σύμφωνα με το θεωρητικό υπόβαθρο της προτεινόμενης μελέτης [Developmental Origins of Health and Disease paradigm (DOHaD)], οι περιβαλλοντικές επιδράσεις κατά τη διάρκεια των ευαίσθητων περιόδων ανάπτυξης μπορεί να προκαλέσουν αλλαγές στην ανάπτυξη με σημαντικές επιπτώσεις στην βραχυπρόθεσμη και μακροπρόθεσμη υγεία του ατόμου ενώ μπορεί να αποτελέσουν επεξηγηματικούς παράγοντες για τις νευροαναπτυξιακές διαταραχές. Η περίοδος ζωής από τη σύλληψη μέχρι την πρώτη παιδική ηλικία είναι καθοριστικής σημασίας για την άμεση και μελλοντική υγεία του βρέφους. Η διάρκεια της κύησης αποτελεί έναν από τους κρίσιμης σημασίας περιγεννητικούς παράγοντες που επηρεάζουν την προδιάθεση στις ασθένειες. Στο πλαίσιο του ίδιου θεωρητικού μοντέλου έχει προταθεί οτι είναι δυνατή η επεξήγηση της σύνδεσης των πρώιμων εμπειριών της ζωής με την μετέπειτα ψυχοπαθολογία. Επιπρόσθετα, είναι πολύ πιθανόν η επίδραση της φτώχειας να ξεκινάει πολύ νωρίς στη ζωή, ακόμα και προγεννητικά. Τα πρόωρα βρέφη που προέρχονται από χαμηλό κοκ επίπεδο έχουν υψηλότερα ποσοστά αναπτυξιακής νοσηρότητας σε σχέση με τα πρόωρα που προέρχονται από υψηλότερο κοινωνικο-οικονομικό επίπεδο ενώ βρίσκονται σε αυξημένο κίνδυνο συμπεριφορικών και συναισθηματικών προβλημάτων (Barker, 1998; Eriksson, 2016; Gluckman and Hanson 2006; Johnson & Marlow, 2011; Mandy & Nyirenda, 2018; Nobile, 2022).

Η ανάπτυξη των πρόωρων βρεφών από χαμηλό κοινωνικο-οικονομικό επίπεδο

Οι βιολογικοί, ψυχολογικοί και κοινωνιολογικοί κίνδυνοι αλληλεπιδρούν με τρόπο που απειλεί την ικανότητα της οικογένειας χαμηλού κοινωνικο-οικονομικού επιπέδου να ανταποκριθεί στην ικανοποίηση των φυσικών, κοινωνικών και συναισθηματικών αναγκών των μελών της (Maurer & Smith, 2013). Κατά την περιγεννητική περίοδο, οι γυναίκες που προέρχονται από χαμηλό κοκ επίπεδο επηρεάζονται δυσανάλογα από τον πρόωρο τοκετό, την κατάθλιψη, το άγχος και από έλλειψη πρόσβασης στη φροντίδα ψυχικής υγείας. Το χαμηλό κοκ αποτελεί ένα από τους πιο καθοριστικούς παράγοντες που συσχετίζονται με την περιορισμένη νευροανάπτυξη των πρόωρων βρεφών. Τα υψηλότερα ποσοστά βιολογικών κινδύνων των πρόωρων βρεφών χαμηλού κοκ επιπέδου τα θέτουν σε διπλό κίνδυνο για χαμηλά νευροαναπτυξιακά αποτελέσματα (Panceri, 2020; Wong & Edwards, 2013). Παρά το γεγονός ότι οι καθυστερήσεις στην ανάπτυξη των πρόωρων βρεφών και εκείνων χαμηλού κοινωνικο-οικονομικού επιπέδου είναι καλά τεκμηριωμένες, υπάρχουν εξαιρετικά περιορισμένες ενδείξεις για την πολύ πρώιμη μεταγεννητική ανάπτυξη των πρόωρων βρεφών που εκτίθενται τόσο σε βιολογικούς όσο και σε περιβαλλοντικούς παράγοντες κινδύνου (Gonzalez-Gomez, 2019). Είναι πιθανόν οι αρνητικές επιδράσεις της φτώχειας να ξεκινούν κατά τα πρώιμα στάδια της ανάπτυξης, πιθανόν προγεννητικά (Blount, 2021; Hurt, 2017; Hosokawa & Katsura, 2018; Maurer & Smith, 2013). Κατά τη διάρκεια των πρώτων 24 μηνών, το κοκ επίπεδο ευθύνεται για την διακύμανση της γνωστικής ανάπτυξης των πρόωρων βρεφών: όσο υψηλότερο είναι το κοκ επίπεδο τόσο υψηλότερη είναι η γνωστική ανάπτυξη ωτν πρόωρων βρεφών (Panceri, 2020). Συγκριτικά με τα πρόωρα βρέφη που προέρχονται από οικογένειες μέσου ή υψηλού κοκ επιπέδου, τα πρόωρα παιδιά από χαμηλό κοκ επίπεδο βρίσκονται σε αυξημένο κίνδυνο συμπεριφορικών και συναισθηματικών προβλημάτων στην ηλικία των 4 ετών (Potijk, 2014).

Η ανάπτυξη των πρόωρων βρεφών από χαμηλό κοινωνικο-οικονομικό επίπεδο και οι σχετικοί ψυχοκοινωνικοί παράγοντες

Μητρική ψυχική υγεία

Οι γυναίκες από χαμηλό κοκ επίπεδο βρίσκονται σε αυξημένο κίνδυνο εκδήλωσης

μεταγεννητικής κατάθλιψης και το περιγεννητικό άγχος είναι συχνότερο σε σχέση με γυναίκες υψηλού κοκ επιπέδου ενώ είναι λιγότερο πιθανόν να λάβουν κατάλληλη φροντίδα για την κατάθλιψη κατά τη μεταγεννητική περίοδο παρατείνοντας τη διάρκεια των συμπτωμάτων (Goyal, 2010; Kozhimannil, 2011). Ωστόσο, η εκδήλωση άγχους και κατάθλιψης κατά τη διάρκεια της εγκυμοσύνης έχει συσχετιστεί με πρόωρο τοκετό και αλληλεπιδρά με χαρακτηριστικά του χαμηλού κοκ επιπέδου ενώ το στρες των γονέων των πρόωρων βρεφών είναι υψηλό και οι γονείς αυτοί μπορεί να αντιμετωπίζουν επιπλέον προκλήσεις (Enlow, 2017). Οι μεταγεννητικές διαταραχές ψυχικής υγείας έχουν συσχετιστεί με αρνητικές επιδράσεις στην υγεία της μητέρας και του βρέφους (van Haeken, 2020).

Οικογενειακή λειτουργικότητα
Το χαμηλό κοκ έχει συσχετιστεί με περισσότερες στρεσογόνες καταστάσεις οικογενειακής ζωής (Reiss, 2019). Το χαμηλό κοκ συσχετίζεται με υψηλότερα επίπεδα συζυγικών προβλημάτων και μπορεί να επιδεινώσει το στρες των δυο συντρόφων με αρνητικές επιπτώσεις για την ψυχολογική τους ευημερία (Conger, 2010). Οι οικογένειες των πρόωρων βρεφών χαμηλού κοκ επηρεάζονται δυσανάλογα από το στρες και οι μητέρες βιώνουν ιδιαίτερο άγχος κατά την περιγεννητική περίοδο (Enlow, 2017). Η οικογενειακή αίσθηση συνοχής, δηλαδή ο συναισθηματικός δεσμός που υπάρχει μεταξύ των μελών της οικογένειας, αποτελεί μια από τις στρατηγικές προσαρμογής στη μετάβαση στη γονεϊκότητα (Ngai & Ngu, 2016; Olson, 2019). Τα ζευγάρια που έχουν ισχυρή αίσθηση συνοχής πιθανόν να μοιράζονται ένα κοινό στόχο στην ανατροφή του παιδιού και ένα κοινό κίνητρο ενεργοποίησης όλων των διαθέσιμων πόρων για την αντιμετώπιση των γονικών απαιτήσεων (Ngai & Ngu, 2016). Τα συγχρονικά και διαχρονικά δεδομένα δείχνουν ότι τα παιδιά που προέρχονται από οικογένειες υψηλής συνοχής αντιμετωπίζουν λιγότερα συναισθηματικά και συμπεριφορικά προβλήματα (Coe, 2018; Shigeto, 2013; Tissot, 2022).

Κοινωνική υποστήριξη
Κατά τη δυαδική αντιμετώπιση του στρες, όπως αυτή που απαιτείται κατά τη γέννηση ενός παιδιού (Bodenmann, 1995, 2005), οι νέοι γονείς αναζητούν πόρους κοινωνικής υποστήριξης. Κατά τη διάρκεια μετάβασης στη γονεϊκότητα, η δυαδική αντιμετώπιση του στρες μειώνει το στρες των συντρόφων, βελτιώνει την ψυχολογική τους ευημερία και αυξάνει τη λειτουργικότητα του ζευγαριού (Bodenmann, 1995; Falconier, 2015). Η κοινωνική υποστήριξη προς τους γονείς των πρόωρων βρεφών μπορεί να αποτελέσει προστατευτικό παράγοντα για την οικογένεια (Leahy-Warren, 2020; Lutkiewicz, 2020). Η απουσία των κοινωνικών σχέσεων απότελεί έναν από τους στρεσογόνους παράγοντες των οικογενειών από χαμηλό κοκ συγκριτικά με τις οικογένειες υψηλού κοκ (Leahy-Warren, 2020; Weyers, 2008). Ο κίνδυνος μεταγεννητικών ψυχολογικών διαταραχών είναι υψηλότερος μεταξύ των γυναικών χαμηλού κοκ που λαμβάνουν ανεπαρκή κοινωνική υποστήριξη (Fisher, 2012). Η κοινωνική υποστήριξη κατά τη μεταγεννητική περίοδο έχει άμεση θετική επίδραση στην οικογενειακή λειτουργικότητα και έμμεση αρνητική επίδραση στην εκδήλωση της κατάθλιψης (Huang, 2021). Τα δίκτυα κοινωνικής υποστήριξης επηρεάζουν το κοινωνικο-οικολογικό περιβάλλον και την ανάπτυξη του βρέφους/ παιδιού και και σχετίζονται με την μητρική κοινωνική υποστήριξη. Ωστόσο, αυτή η σχέση δεν έχει μελετηθεί εκτενώς σε συνθήκες φτώχειας ιδιαίτερα κατά τη διάρκεια των πρώτων 9 μηνών της ζωής (Singletary, 2021).

Διυποκειμενικότητα και δεσμός γονέα-βρέφους
Η διυποκειμενικότητα αποτελεί μια διαδικασία που καθιστά δυνατή τη δυνατότητα για τα υποκείμενα να ανιχνεύσουν και να αλλάξουν τη συμπεριφορά του άλλου μέσω εμπρόθετων συγκινησιακών εκφράσεων, προθέσεων και ενδιαφερόντων (Trevarthen, 2001, p.18). Η μητρική αντίληψη για τη συμμετοχή του βρέφους στην αλληλεπίδραση μητέρας –βρέφους καθορίζει την ανάπτυξη της παραπάνω σημαντικής εμπειρίας των βρεφών. Η πρόωρη γέννηση φαίνεται να επηρεάζει δυσμενώς τις διυποκειμενικές αλληλεπιδράσεις μητέρας-

βρέφους (Carrulo, 2022). Η απουσία της διυποκειμενικής επικοινωνίας στην αλληλεπίδραση μητέρας-βρέφους παρεμβαίνει στην ανάπτυξη κοινωνικο-συναισθηματικών δεξιοτήτων που σχετίζονται με τις νευρο-αναπτυξιακές διαταραχές (Trevarthen & Aitken, 2001). Παράλληλα, τα πρόωρα βρέφη αποτελούν ομάδα κινδύνου όσον αφορά τη διαμόρφωση δεσμού (Korja, 2012; Leahy-Warren, 2020). Τα αναπνευστικά προβλήματα των πρόωρων βρεφών αποτελούν παράγοντα κινδύνου που μπορεί να επηρεάσουν τη διαδικασία ανάπτυξης του δεσμού (Korja, 2012; Leahy-Warren, 2020). Η οικογενειακή συνοχή συσχετίζεται στενά με το δεσμό (Youngblut et al 1993). Κατά τη διάρκεια του πρώτου έτους ζωής, το υψηλό επίπεδο μητρικών καταθλιπτικών συμπτωμάτων έχει συσχετιστεί με την πρόωρη γέννηση, το φτωχό δεσμό και την ελλειμματική κοινωνική υποστήριξη. Παραδόξως, υπάρχουν ενδείξεις υψηλής ποιότητας δεσμού της μητέρας προς το πρόωρο βρέφος της κατά τη διάρκεια του πρώτου χρόνου ζωής (Leahy-Warren, 2020). Ωστόσο, κάποιες μητέρες πρόωρων βρεφών περιγράφουν ανάμεικτες συναισθηματικές εμπειρίες σχετικά με τη διαδικασία δεσμού με το παιδί τους (Widding & Farooqi, 2016). Ο συνδυασμός της πρόωρης γέννησης με το χαμηλό κοκ μπορεί να παρεμποδίσει τη διαμόρφωση του δεσμού και πιθανόν να συσχετιστεί με δυσμενείς αναπτυξιακές δυσκολίες (Hoffenkamp, 2012; Wille, 1991).

Βιολογικοί παράγοντες και η ανάπτυξη των πρόωρων βρεφών από χαμηλό κοκ

Μελατονίνη

Εκτός από τη συσχέτισή της με τον κιρκάδειο ρυθμό, η μελατονίνη έχει ένα ευρύ φάσμα βιολογικών λειτουργιών με επιδράσεις στην καρδιαγγειακή υγεία ιδιαίτερα για τα βρέφη (Katzer,2016; Qin,2019; Gombert & Codoñer-Franch, 2021). Η μελατονίνη στη μητρικό γάλα είναι σημαντική για την φυσιολογική νευροανάπτυξη και παίζει σημαντικό ρόλο στην εναρμόνιση του ρυθμού του καρδιαγγειακού συστήματος του νεογνού με εκείνον της μητέρας, στοιχείο σημαντικό για τη νεογνική ομοιόσταση και λειτουργία το οποίο πιθανόν να συνεισφέρει στην καλύτερη μακροπρόθεσμη ανάπτυξη (Gombert & Codoñer-Franch, 2021).

Η μελατονίνη ανιχνεύεται στις 24 εβδομάδες κύησης, φτάνει στο ανώτατο σημείο κατά το τρίτο τρίμηνο της κύησης. Κατά τη διάρκεια των πρώτων 3 μηνών ζωής, τα βρέφη έχουν μια μεταβατική απώλεια μελατονίνης λόγω της ελλειπούς παραγωγής μελατονίνης και του ανώριμου κιρκάδειου ρυθμού (D'Angelo,2020). Το μητρικό γάλα αποτελεί τη μοναδική πηγή μελατονίνης για το βρέφος και ειδικά για τα πρόωρα βρέφη κατά τη διάρκεια των πρώτων μηνών της ζωής τους (Gombert & Codoñer-Franch, 2021). Τα παραπάνω είναι σημαντικά για τα πρόωρα νεογνά καθώς δεν έχουν ολοκληρώσει την φυσιολογική ηλικία κύησης που θα τους παρείχε τα μέγιστα επίπεδα μελατονίνης ενώ βρίσκονται σε κίνδυνο οξειδωτικού στρες και έχουν καθυστέρηση στη ρυθμική έκφραση της μελατονίνης συγκριτικά με τα τελειόμηνα βρέφη. Ωστόσο το μητρικό γάλα των γυναικών που έχουν γεννήσει πρόωρα έχει υψηλότερη συγκέντρωση μελατονίνης συγκριτικά με το μητρικό γάλα γυναικών που έχουν ολοκληρώσει τη φυσιολογική ηλικία κύησης. Δεν είναι γνωστό σε ποιο βαθμό το κοκ επηρεάζει τη σύσταση του μητρικού γάλακτος (Italianer, 2020; Samuel, 2020). Το χαμηλό κοκ ίσως επιδεινώνει τον κίνδυνο χαμηλού διατροφικού επιπέδου των εγκύων και οι διατροφικές ανεπάρκειες μπορεί να επηρεάζουν τη σύσταση του μητρικού γάλακτος που σχετίζεται με την κιρκάδεια ρυθμικότητα (Freisling, 2006 Italianer, 2020). Επιπλέον, το μητρικό μεταγεννητικό στρες και η αρνητική διάθεση έχει συσχετιστεί με υψηλά επίπεδα μελατονίνης στο δείγμα μητρικού γάλακτος (Groër, 2005). Ο ρόλος της μελατονίνης στην ανάπτυξη των πρόωρων βρεφών είναι ασαφής. Στο βαθμό που γνωρίζουμε μόνο μια μελέτη έδωσε ενδείξεις ότι η βελτιωμένη λειτουργία του αυτόνομου νευρικού συστήματος στις 2 εβδομάδες συσχετίστηκε με καλύτερες επιδόσεις σε κλίμακες νοητική ανάπτυξης στους 9 μήνες όταν υπήρχε συσχέτιση και με τα επίπεδα μελατονίνης στους 4, 6 και 9 μήνες ζωής (Goldstein Ferber, 2011). Είναι σημαντικό να μελετηθεί η μελατονίνη που προέρχεται από το μητρικό γάλα καθώς οι διαταραχές μελατονίνης έχουν συσχετιστεί με μια σειρά ψυχιατρικών και

αναπτυξιακών διαταραχών, όπως η κατάθλιψη, η σχιζοφρένεια, ο αυτισμός κλπ (Tordjman, 2017).

Η ωρίμανση του αυτόνομου νευρικού συστήματος και η μεταβλητότητα του καρδιακού ρυθμού

Σε βρέφη που γεννιούνται με την ολοκλήρωση της ηλικίας κύησης, η φυσιολογική αύξηση του παρασυμπαθητικού τόνου εκδηλώνεται μέσω της υψηλής συχνότητας της μεταβλητότητας του καρδιακού ρυθμού (HRV) (Mulkey & du Plessis, 2019). Η ηλικία κύησης συσχετίζεται με τον καρδιακό ρυθμό καθώς και με παραμέτρους που σχετίζονται με τη μεταβλητότητα του καρδιακού ρυθμού, όσο μικρότερη είναι η ηλικία κύησης τόσο χαμηλότερη είναι η μεταβλητότητα του καρδιακού ρυθμού. Η μειωμένη HRV έχει συσχετιστεί με ευαλωτότητα στο στρες ενώ η αυξημένη HRV αντιπροσωπεύει φυσική και νοητική προσαρμοστικότητα (Javorka, 2017; Suga, 2019). Έρευνες έχει δείξει μια συσχέτιση ανάμεσα στην ωρίμανση του αυτόνομου νευρικού συστήματος των πρόωρων βρεφών με βραχυπρόθεσμα και μακροπρόθεσμα αναπτυξιακά αποτελέσματα που αφορούν την κοινωνική και συναισθηματική ανάπτυξη, την εκδήλωση προβλημάτων εξωτερίκευσης και εσωτερίκευσης καθώς και γνωστικά προβλήματα και νευροψυχιατρικές διαταραχές στα παιδιά (Doussard-Roosevelt, 1997; Doussard-Roosevelt, McClenny & Porges, 2001; Field & Diego, 2008; Graziano & Derefinko, 2013; Mulkey & de Plessis, 2019; Porges & Furman, 2011). Επιπλέον, τα νεογνά καταθλιπτικών μητέρων έχουν χαμηλότερο καρδιακό τόνο συγκριτικά με εκείνα μητέρων που δεν έχουν αντίστοιχα συμπτώματα (Jones, 1998; Field, 1995). Μόνο ένας περιορισμένος αριθμός μελετών έχει δώσει αντιφατικά αποτελέσματα που αφορούν τη σχέση της λειτουργικότητας των γονέων με μετρήσεις ωρίμανσης του αυτόνομου νευρικού συστήματος νωρίς στη ζωή (Porter, 2003; Moore , 2010; Graham, 2010).

Η νέα γνώση που θα προσφέρει το προτεινόμενο ερευντητικό έργο

Το προτεινόμενο ερευνητικό έργο είναι καινοτόμο ως εξής: (1) Ο ρόλος της μελατονίνης στην ανάπτυξη των πρόωρων βρεφών είναι ασαφής (Tauman, 2002) και δεν έχει μελετηθεί επαρκώς. Προς την κατεύθυνση αυτή, σκοπεύουμε να μελετήσουμε το ρόλο της μελατονίνης μέσα από ένα μονοπάτι συνδυαστικών επιδράσεων μιας σειράς ψυχοκοινωνικών και βιολογικών παραγόντων κατά την διάρκεια της πρώιμης ανάπτυξης; (2) Το προτεινόμενο έργο που αφορά τους παράγοντες που σχετίζονται με την ανάπτυξη των πρόωρων βρεφών είναι διαχρονικό. Οι σχετικές διαχρονικές μελέτες που αφορούν την ανάπτυξη των πρόωρων βρεφών είναι σπάνιες. Προκειμένου να κατανοήσουμε τις δυσκολίες στην ανάπτυξη των πρόωρων βρεφών θα πρέπει να τα παρακολουθήσουμε κατά την διάρκεια των αναπτυξιακών τους σταδίων (Jansen, 2021); (3) Το προτεινόμενο ερευνητικό έργο θα υιοθετήσει μεικτή μεθοδολογία η οποία περιλαμβάνει μετρήσεις φυσιολογίας και ορμονικές, τη χρήση ενός εργαλείου παρατήρησης καθώς και ερωτηματολόγια αυτο-αναφοράς. Η χρήση δεδομένων που προέρχονται από μεικτή μεθοδολογία στη βρεφική έρευνα αποτελεί έναν τρόπο αύξησης της εγκυρότητας των αποτελεσμάτων ενώ ταυτόχρονα μας δίνει τη δυνατότητα να ερμηνεύουμε με μεγαλύτερη ακρίβεια τα δεδομένα μας (LoBue, 2020); (4) Τα στοιχεία που αφορούν την ανάπτυξη των πρόωρων βρεφών στην Ελλάδα είναι περιορισμένα (Koutra, 2012). Στο βαθμό που γνωρίζουμε το προτεινόμενο έργο είναι το πρώτο στην Κρήτη, Ελλάδα, μετά την έναρξη της οικονομικής κρίσης και της πανδημίας. Αυτό είναι πολύ σημαντικό καθώς η οικονομική κρίση της τελευταίας δεκαετίας έχει προκαλέσει την αύξηση στο αριθμό των ατόμων που ζούν σε συνθήκες φτώχειας στην Ελλάδα και έχει οδηγήσει στην αναστολή λειτουργίας των υπηρεσιών φροντίδας ψυχικής υγείας (Papadakaki, 2021; Tsobanoglou, 2014;_Giannopoulou & Tsobanoglou, 2020). Επιπλέον, η πανδημία έχει προκαλέσει την αύξηση στις ανισοτήτες εισοδήματος (Vavoura & Vavouras, 2022) και έχει επηρεάσει αρνητικά την ψυχική υγεία των Ελλήνων (Parlapani, 2020; Vatavali, 2020). Στην Κρήτη, η φτώχεια και τα προβλήματα ψυχικής υγείας είναι αυξημένα και συσχετίζονται μεταξύ τους, ιδιαίτερα για τις γυναίκες. Τα ποσοστά των οικογενειών με πολλαπλά προβλήματα είναι ιδιαίτερα αυξημένα (Papadakaki, 2021). Επιπρόσθετα, οι πρόωρες γεννήσεις αποτελούν ένα σημαντικό ζήτημα δημόσιας υγείας στην Ελλάδα (Vlachadis,

2013). Τα ποσοστά των πρόωρων γεννήσεων έχουν 4πλασιαστεί κατά την διάρκεια των δύο τελευταίων δεκαετιών. Αυτά τα ευρήματα θέτουν δραματικές προκλήσεις για τη δημόσια υγεία και αναδεικνύουν την αναγκαιότητα για την υλοποίηση προληπτικών παρεμβάσεων (Vlachadis, 2013).

Οι επιπτώσεις του προτεινόμενου έργου για την επιστήμη και την κοινωνία

Από την πλευρά της Ιατρικής, η δραστηριότητα του αυτόνομου νευρικού συστήματος αποτελεί έναν δείκτη υγείας και έχει σημαντικό ρόλο στην διατήρηση της καρδιαγγεακής και αναπνευστικής ομοιόστασης ενώ ταυτόχρονα συνδέεται και με ανώτερα εγκεφαλικά συστήματα τα οποία εμπλέκονται σε συναισθηματικές και ψυχολογικές όψεις της ανθρώπινης ζωής στο πεδίο της επιστήμης της Ψυχολογίας (Mulkey & du Plessis, 2019; Porges & Furman, 2011). Επιπλέον, στο πλαίσιο της Ψυχολογίας, η μελατονίνη είναι σημαντική για την φυσιολογική νευροανάπτυξη ενώ στο πεδίο της Ιατρικής αντικείμενο έρευνας αποτελεί η πιθανή νευροπροστατευτική δράση της μελατονίνης στα πρόωρα νεογνά όπως έχει φανεί σε προκλινικά μοντέλα (Biran, 2019).
Τα προβλήματα περιγεννητικής ψυχικής υγείας αποτελούν σημαντικά ζητήματα δημόσιας υγείας σε διεθνές επίπεδο (Tripathy, 2020). Οι διαταραχές περιγεννητικής ψυχικής υγείας έχουν μακροπρόθεσμες επιδράσεις στην συμπεριφορική λειτουργικότητα και στην υγεία της φυσιολογίας καθόλη τη διάρκεια της ζωής με πιθανότητα διαγενεαλογικής μεταβίβασης (Coussons-Read, 2013). Επιπρόσθετα, η προωρότητα θεωρείται μια χρόνια και πολυγενεαλογική συνθήκη (Pravia & Benny, 2020). Η πρόληψη προϋποθέτει αποτελεσματικές παρεμβάσεις που στοχεύουν και στις γυναίκες που βρίσκονται σε κίνδυνο αλλά πριν την ανάπτυξη της καταθλιπτικής διαταραχής. Οι πρώιμες παρεμβάσεις μπορεί να βελτιώσουν την υπο-κλινική συμπτωματολογία για τις δυάδες που βρίσκονται σε κίνδυνο αλλά στην κρίσιμη πρώιμη μεταγεννητική περίοδο (Scorza, 2020). Σε σύνδεση με τα παραπάνω, οι καθυστερήσεις στην ανάπτυξη των πρόωρων βρεφών μπορεί να γίνουν αντικείμενο πρόληψης και πρώιμης παρέμβασης. Η πρόωρη ανίχνευση των αναπτυξιακών καθυστερήσεων αποτελεί μια διαδικασία συνεχούς αξιολόγησης και μελέτης βασικών περιγεννητικών παραγόντων που φαίνεται να επηρεάζουν μακροπρόθεσμα τα αναπτυξιακά αποτελέσματα (Kwong et al 2022). Τα αποτελέσματα της προτεινόμενης μελέτης μπορεί να αναδείξουν την αναγκαιότητα για μελλοντικές κοινοτικές παρεμβάσεις και ίσως πείσουν εκείνους που χαράζουν την κοινωνική πολιτική να αυξήσουν τις παρεμβάσεις που βασίζονται σε επιστημονικά δεδομένα και στη φροντίδα που εστιάζει στην οικογένεια για την προαγωγή της περιγεννητικής ψυχικής υγείας των νέων μητέρων και την ανάπτυξη των πρόωρων βρεφών που προέρχονται από οικογένειες χαμηλού κοινωνικο-οικονομικού επιπέδου. Ο σχεδιασμός ψηφιακών παρεμβάσεων για την υγεία με σκοπό τη βελτίωση στην πρόσβαση της ανίχνευσης αναπτυξιακών διαταραχών - αυξάνοντας τις υπηρεσίες στην οικία των ευάλωτων πληθυσμών (Van der Merwe, 2019) - μπορεί να πάρει τη μορφή στοχευμένων παρεμβάσεων με σημαντικά οφέλη στη δημόσια υγεία. Επιπλέον, τα αποτελέσματα του προτεινόμενου ερευνητικού έργου ίσως αναδείξουν την αναγκαιότητα περαιτέρω συζητήσεων για τις προτεραιότητες στην χάραξη κοινωνικής πολιτικής και την ευθύνη της κοινωνίας στην αντιστάθμιση ανισοτήτων στα θέματα φροντίδας της υγείας (M'hamdi, 2017) αλλά και στη διακοπή της διαγενεαλογικής μεταβίβασης της ψυχικής ασθένειας και των αντιξοοτήτων (Prom, 2022). Αυτό είναι σημαντικό καθώς η βιβλιογραφία που αφορά την περιγεννητική ψυχική υγεία εστιάζει ατομικά στις γυναίκες ως το βασικό φορέα αλλαγών ενώ καθοριστικοί κοινωνικοί παράγοντες της ψυχικής υγείας, όπως η φτώχεια, είναι κρίσιμης σημασίας για τις γυναίκες κατά την περιγεννητική περίοδο (Howard & Khalifeh, 2020). Στο οικονομικό επίπεδο, η αναγνώριση των πρώιμων παραγόντων κινδύνου για την ανάπτυξη των πρόωρων βρεφών μπορεί να έχει επιπτώσεις για την χάραξη της σχετικής πολιτικής αφού τα πρόωρα βρέφη, συγκριτικά με τα τελειόμηνα βρέφη, έχουν υψηλότερο κόστος πρώιμης περίθαλψης και παρέμβασης (Clements, 2007).

ΜΕΘΟΔΟΛΟΓΙΑ

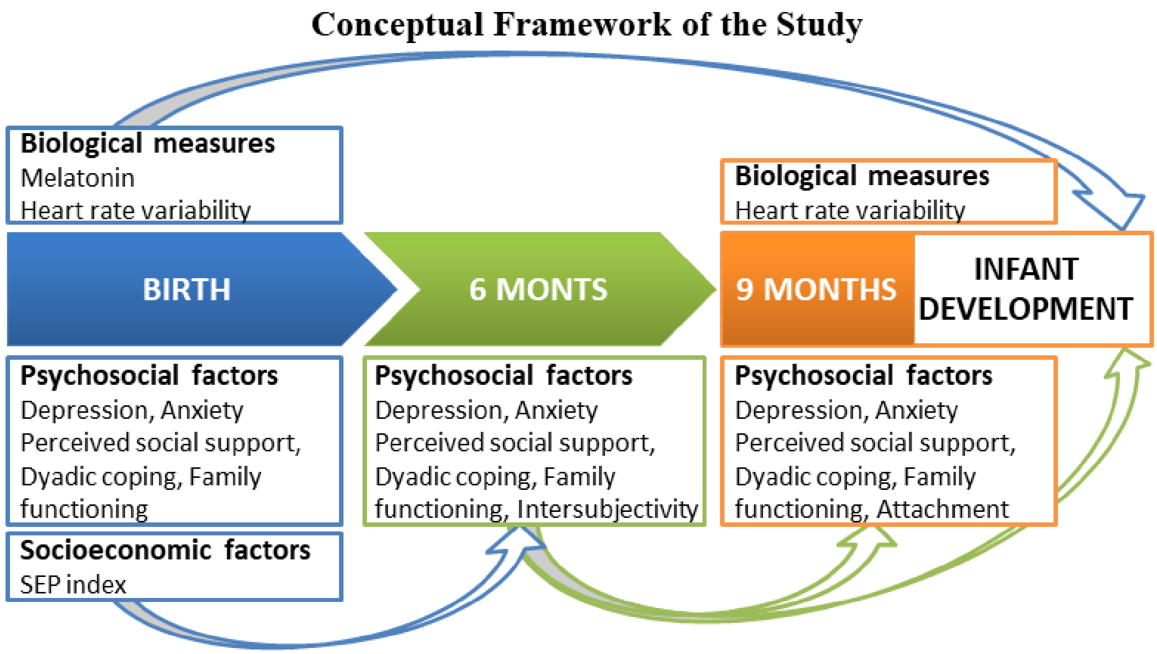

Διάγραμμα: Εννοιολογικό πλαίσιο του προτεινόμενου ερευνητικού έργου και χρονοδιάγραμμα των αξιολογήσεων των ψυχοκοινωνικών και βιολογικών παραγόντων

Συμμετέχοντες
Μητέρες με τα πρόωρα βρέφη τους (<37 εβδομάδων κύησης) (για διάστημα από τη γέννηση μέχρι διορθωμένη ηλικία 9 μηνών) που θα νοσηλευτούν στη Νεογνολογική Κλινική, Μονάδα Εντατικής Νοσηλείας Νεογνών του ΠΑΓΝΗ κατά τη διάρκεια του προγράμματος. Οι συμμετέχοντες της έρευνας θα αντληθούν μέσω της Γυναικολογικής-Μαιευτικής Κλινικής και της Νεογνολογικής Κλινικής, Μονάδας Εντατικής Νοσηλείας Νεογνών (ΜΕΝΝ) του Πανεπιστημιακού Γενικού Νοσοκομείου Ηρακλείου.

Κριτήρια εισδοχής στο έργο
Τα κριτήρια συμπερίληψης των συμμετεχόντων στο πρόγραμμα αποτελούν: α) οι γονείς δεν θα πρέπει να είναι διαζευγμένοι, β) οι δύο γονείς θα πρέπει να είναι άνω των 20 ετών, και γ) η μητέρα θα πρέπει να θηλάζει για το διάστημα τουλάχιστον των πρώτων 28 ημερών ζωής του παιδιού της. Τα κριτήρια συμπερίληψης για τα νεογνά είναι η προωρότητα (ηλικία κύησης < 37 εβδομάδων) και η απουσία σοβαρών νευρολογικών / εγκεφαλικών προβλημάτων.

Κριτήρια αποκλεισμού από το έργο
Τα κριτήρια αποκλεισμού των συμμετεχόντων αφορούν: α) το ιστορικό ψυχιατρικής ασθένειας, β) τα προβλήματα χρήσης ναρκωτικών ή άλλων ουσιών, γ) η μη βιολογική οικογένεια του βρέφους, δ) οι γονείς να είναι ζευγάρι ομοφυλόφιλων. Ο αποκλεισμός των ομοφυλόφιλων ζευγαριών στην προτεινόμενη μελέτη τεκμηριώνεται από την έλλειψη στοιχείων από σχετίζονται με την πρώιμη ανάπτυξη των πρόωρων βρεφών/παιδιών ομόφυλων ζευγαριών στην Ελλάδα. Η έλλειψη αυτή θα είχε ως

αποτέλεσμα την αδυναμία συγκρίσεων και τελικής αξιοποίησης των αποτελεσμάτων, ε) μητέρες που δεν επιθυμούν να θηλάσουν, στ) οι παρένθετες μητέρες, ζ) μητέρες από άλλο γεωγραφικό διαμέρισμα που δεν μπορούν να προσέλθουν στο follow-up της μελέτης (διορθωμένη ηλικία 9 μηνών).

Διαδικασία Συλλογής Δεδομένων
Τα μέλη της ερευνητικής ομάδας που θα είναι υπεύθυνα για την προσέγγιση των συμμετεχόντων και τη συλλογή των δεδομένων (Ν.Α. και μη ονοματισμένο μέλος) θα επικοινωνούν με τις μητέρες και θα τις ενημερώνουν σχετικά με την έρευνα (στόχος της έρευνας, διαδικασία και διάρκεια συμμετοχής, διασφάλιση της ανωνυμίας και της εμπιστευτικότητας, εθελοντικός χαρακτήρας της συμμετοχής καθώς και ευρύτερη ωφελιμότητα της έρευνας). Έτσι θα δίνεται η δυνατότητα σε κάθε μητέρα να αποφασίσει αβίαστα την συμμετοχή της ίδιας και του νεογνού της στην έρευνα έχοντας πλήρη ενημέρωση για όσα συμπεριλαμβάνονται σε αυτή. Πριν την υπογραφή των σχετικών εντύπων συγκατάθεσης, οι ερευνήτριες θα προσφέρουν επιπλέον διευκρινήσεις και θα απαντούν σε τυχόν απορίες, συμπεριλαμβάνοντας σε κάθε περίπτωση την ενημέρωση για τα οφέλη από τη συμμετοχή στην έρευνα. Επιπλέον οι μητέρες θα ενημερωθούν με σχετική επιστολή για τη δυνατότητα υποβολής παραπόνων ή καταγγελιών στην Επιτροπή Ηθικής και Δεοντολογίας της Έρευνας του Πανεπιστημίου Κρήτης, στην Επιτροπή Ηθικής και Δεοντολογίας της Έρευνας του Πανεπιστημιακού Γενικού Νοσοκομείου Ηρακλείου και στην Υπεύθυνη Επεξεργασίας Προσωπικών Δεδομένων του Πανεπιστημίου Κρήτης (βλ. παρακάτω). Μετά την αναλυτική παρουσίαση της ερευνητικής διαδικασίας και την παροχή απαντήσεων σε τυχόν ερωτήματα, θα ληφθεί έγγραφη ενήμερη συγκατάθεση για την εθελοντική συμμετοχή των μητέρων σύμφωνα με τους όρους της Διακήρυξης του Helsinki (World Medical Association Declaration of Helsinki, Ethical Principles for Medical Research Involving Human Subjects).

Μετρήσεις
Α.Κοινωνικό-οικονομικοί παράγοντες
Στο πλαίσιο του προτεινόμενου ερευνητικού έργου, θα αξιολογήσουμε διάφορους στρεσογόνους παράγοντες που εμφανίζονται κατά τη διάρκεια της εγκυμοσύνης και των πρώιμων στάδιων της ζωής με σημαντικές επιδράσεις στην υγεία και στην ανάπτυξη. Η συλλογή των δεδομένων θα αφορά την αξιολόγηση παραγόντων που σχετίζονται με το κοινωνικο-οικονομικό επίπεδο, όπως είναι το επίπεδο εκπαίδευσης των γονέων, το επάγγελμά τους, ο τύπος επαγγελματικού συμβολαίου/σύμβασης, το οικογενειακό εισόδημα, το μέγεθος της οικογένειας, η φροντίδα των παιδιών, η στέγαση και η έκταση αυτής. Τα στοιχεία αυτά θα συνδυαστούν προκειμένου να διαμορφωθεί ένας αλγόριθμος που θα δώσει τη δυνατότητα σύνδεσης με τα *European Union Statistics on Income and Living Conditions (EUSILC)* προκειμένου να αποκτήσουμε πληροφορίες για το κοινωνικο-οικονομικό επίπεδο και τις κοινωνικές μειονεξίες νωρίς στη ζωή. Ακολούθως, ο συνδυασμός των σχετικών πληροφοριών θα μας βοηθήσει στη διαμόρφωση του δείκτη της κοινωνικο-οικονομικής θέσης (Pizzi, 2020).

Β. Ψυχοκοινωνικοί παράγοντες

Μητρική Κατάθλιψη
Η κλίμακα *Edinburgh Postnatal Depression Scale* (EPDS, Cox, 1987) θα χρησιμοποιηθεί για την ανίχνευση πιθανόν καταθλιπτικών συμπτωμάτων στις νέες μητέρες. Η EPDS είναι μια κλίμακα αυτο-αναφοράς που αποτελείται από 10 items και είναι σχεδιασμένη για πληθυσμούς εγκύων και νέων μητέρων κατά την διάρκεια της περιγεννητικής και μετα-γεννητικής περιόδου. Κάθε ερώτηση βαθμολογείται από 0-3. Η EPDS έχει σταθμιστεί για μια

σειρά κοινοτικών δειγμάτων με την πλειοψηφία των σχετικών μελετών να επικεντρώνονται στην περίοδο των 6-8 εβδομάδων μεταγεννητικά (Chaudron, 2010). Η EPDS έχει σταθμιστεί και για τον ελληνικό πληθυσμό (Vivilaki, 2009).

Το ερωτηματολόγιο *The Beck Depression Inventory-II* (BDI-II; Beck, 1996) θα χρησιμοποιηθεί για την ανίχνευση των μητρικών συμπτωμάτων κατάθλιψης κατά τη διάρκεια της προηγούμενης εβδομάδας της χορήγησης του. Το BDI-II είναι ένα ερωτηματολόγιο αυτο-αναφοράς αποτελούμενο από 21 ερωτήσεις με 4 επιλογές απόντησης για κάθε ερώτηση. Το BDI-II έχει σταθμιστεί στο ελληνικό πληθυσμό (Giannakou, 2013).

Μητρικό άγχος

Το *Spielberger State-Trait Anxiety Inventory for Adults* (STAI, Spielberger, 1983) θα χρησιμοποιηθεί για την αξιολόγηση του μητρικού άγχους. Το STAI είναι ένα εργαλείο αυτο-αναφοράς αποτελούμενο από 40 ερωτήσεις με 4βάθμια κλίμακα απαντήσεων για κάθε ερώτηση. Το STAI αποτελείται από 2 κλίμακες: την State anxiety (η οποία αξιολογεί το πως αισθάνεται κάποιος τη στιγμή της αξιολόγησης, 20 ερωτήσεις) και την Trait anxiety (η οποία αξιολογεί το πως αισθάνεται κάποιος γενικά, 20 ερωτήσεις). Η STAI έχει σταθμιστεί στον ελληνικό πληθυσμό (Liakos, 1984).

Οικογενειακή λειτουργικότητα

Το *Family Adaptability and Cohesion Evaluation Scales IV Package* (FACES IV; Olson, 1979; Olson, 2019) θα χρησιμοποιηθεί για την αξιολόγηση της οικογενειακής λειτουργικότητας. Το FACES IV Package αποτελείται από 6 κλίμακες για την FACES IV (42 ερωτήσεις), από την Family Communication Scale (FCS) και από την Family Satisfaction Scale (FSS) (62 ερωτήσεις συνολικά). Η FACES έχει σταθμιστεί στον ελληνικό πληθυσμό (Koutra, 2013)

Αντιλαμβανόμενη κοινωνική υποστήριξη

Η *Multidimensional Scale of Perceived Social Support* (MSPSS, Zimet, 1988) θα χρησιμοποιηθεί για την αξιολόγηση της κοινωνικής υποστήριξης που λαμβάνουν οι μητέρες από τις εξής τρείς πηγές (καθεμία από τις οποίες αντιστοιχεί σε μια υποκλίμακα): οικογένεια, φίλοι, και σημαντικοί άλλοι. Η MSPP αποτελεί μια αξιόπιστη και έγκυρη κλίμακα αυτο-αναφοράς αποτελούμενη από 12 ερωτήσεις (κάθε υποκλίμακα αποτελείται από 4 ερωτήσεις). Η MSPSS έχει σταθμιστεί στον ελληνικό πληθυσμό (Theofilou, 2015).

Δυαδική αντιμετώπιση του στρες

Το *Dyadic Coping Inventory* (DCI, Bodenmann, 2008; Ledermann, 2010) θα χρησιμοποιηθεί για τη μέτρηση των συμπεριφορών δυαδικής αντιμετώπισης του στρες. Το DCI αποτελεί ένα αξιόπιστο και έγκυρο εργαλείο 37 ερωτήσεων με 10 υποκλίμακες. Το DCI έχει σταθμιστεί στον ελληνικό πληθυσμό (Roussi & Karademas, 2016).

Διυποκειμενικότητα

Το *Maternal Perception of Infant's Intersubjectivity Questionnaire* (MPIIQ, Carrulo, 2022) θα χρησιμοποιηθεί για την αξιολόγηση της μητρικής αντίληψης της διυποκειμενικότητας του βρέφους. Το MPIIQ είναι ένα ερωτηματολόγιο αυτο-αναφοράς αποτελούμενο από 22 ερωτήσεις με καλά ψυχομετρικά χαρακτηριστικά. Οι ερωτήσεις ομαδοποιούνται στους εξής τρείς παράγοντες: ο παράγοντας 1 σχετίζεται με την μητρική αντίληψη της ικανότητας του βρέφους για αλληλεπίδραση με τη μητέρα, ο παράγοντας 2 αναφέρεται στη μητρική αντίληψη των βρεφικών συμπεριφορών που εκφράζουν συναισθηματικές καταστάσεις ενώ ο παράγοντας 3 σχετίζεται με τις μητρικές αντιλήψεις για την ικανότητα του βρέφους να εκδηλώνει πρωτοβουλίες.

Δεσμός

Η *Maternal Postnatal Attachment Scale* (MPAS, Condon & Corkingdale, 1998) θα χρησιμοποιηθεί για την αξιολόγηση των μητρικών υποκειμενικών συναισθημάτων δεσμού προς το βρέφος. Η MPAS είναι μια κλίμακα αυτο-αναφοράς αποτελούμενη από 19

ερωτήσεις. Η MPAS αποτελείται από τρείς υποκλίμακες: την ποιότητα δεσμού (9 ερωτήσεις), τηςν απουσία εχθρότητας προς το βρέφος (5 ερωτήσεις) και την ευχαρίστηση κατά την αλληλεπίδραση (5 ερωτήσεις).

Το χρονοδιάγραμμα για την χορήγηση των παραπάνω κλιμάκων απεικονίζεται στο παραπάνω διάγραμμα

Γ.Βιολογικές μετρήσεις

Μελατονίνη

Συγκέντρωση μελατονίνης στο μητρικό γάλα

Θα ζητηθεί από τις μητέρες των πρόωρων βρεφών να συλλέξουν 5-10 ml μητρικού γάλακτος με την χρήση μιας ηλεκτρικής αντλίας μεταξύ 10:00-14:00 σε τρία συγκεκριμένα χρονικά σημεία: 3η-5η ημέρα (πρωτόγαλα), 10η-14η ημέρα (μεταβατικό γάλα) και 20η-28η ημέρα (ώριμο γάλα). Η συλλογή του μητρικού γάλακτος θα γίνει σε αποστειρωμένο δοχείο, θα μεταφερθεί στους 4°C και αμέσως θα καταψυχθεί στους -20^{0}C μέχρι να αναλυθεί. Τα επίπεδα μελατονίνης στο μητρικό γάλα θα προσδιοριστούν με την μέθοδο ELISA σύμφωνα με τις οδηγίες του κατασκευαστή.

Συγκέντρωση μελατονίνης σε δείγμα αίματος των νεογνών και του ομφάλιου λώρου

Η συλλογή δείγματος αίματος από τον ομφάλιο λώρο ποσότητας 1-2 ml από κάθε πρόωρο τοκετό ή καισαρική τομή θα γίνει προκειμένου να μετρήσουμε τα επίπεδα μελατονίνης κατά τη γέννηση. Η συλλογή του δείγματος αίματος από τα πρόωρα νεογνά θα γίνει ως εξής:

α. Για τα πρόωρα βρέφη ηλικίας > 33 εβδομάδων, 2 δείγματα θα συλλεχθούν (4η-7η ημέρα ζωής και 10η-14η ημέρα, η οποία θα συμπίπτει με την ηλικία κύησης 35-36 εβδομάδων); β.για τα πρόωρα νεογνά ηλικίας κύησης <33 εβδομάδων, 3 δείγματα θα συλλεχθούν (4η-7η ημέρα ζωής, 10η-14η ημέρα και σε ημερομηνία που θα συμπίπτει με την ηλικία κύησης των 35-36 εβδομάδων).

Η συλλογή του αίματος θα γίνει από το προσωπικό της κλινικής στο πλαίσιο και άλλων προγραμματισμένων δειγματοληψιών, έτσι ώστε τα νεογνά να μην υποβληθούν σε επιπλέον παρεμβάσεις για τους σκοπούς της συγκεκριμένης μελέτης. Ο χρόνος της δειγματοληψίας αίματος θα είναι μεταξύ 8:00-10:00 π.μ.και η ποσότητα εκτιμάται στα 500μL.

Το δείγμα αίματος από τον ομφάλιο λώρο και από το νεογνό θα φυγοκεντριστεί στα 3000 g για 5 λεπτά, και μετά θα γίνει ο διαχωρισμός του πλάσματος. Ακολούθως, τα δείγματα θα καταψυχθούν στους -20°C μέχρι την ανάλυση. Τα επίπεδα μελατονίνης θα προσδιοριστούν με τη χρήση της μεθόδου ELISA σύμφωνα με τις οδηγίες του κατασκευαστή.

.

Μεταβλητότητα του καρδιακού παλμού

Οι μετρήσεις του νεογνικού/βρεφικού και μητρικού ECG θα γίνουν σε 2 διαδοχικές χρονικές περιόδους [για τα νεογνά/βρέφη στη νεογνική ηλικία και στους 9 μηνές μετά τη γέννηση (διορθωμένη ηλικία) και για τις μητέρες κατά τη γέννηση] και οι μετρήσεις της μεταβλητότητας του καρδιακού ρυθμού θα γίνουν μέσω της βραχυπρόθεσμης μεταβλητότητας (short-term variability) η οποία παρέχει σημαντικές πληροφορίες για την ωρίμανση του αυτόνομου νευρικού συστήματος των νεογνών (Lavanga, 2021; Longin, 2006). Οι παράμετροι της HRV που θα αξιολογηθούν είναι οι εξής: time-domain (SDNN, HRm, HRstd, RMSSD, NN50, pNN50, HRV triangular index), the frequency-domain (Total power, LF, HF, LF/HF, LF_{norm}, HF_{norm}) καθώς και οι non-linear indices (ApEn, DFA $α_1$, $α_2$) (Giannakakis, 2019) προκειμένου να προσδιοριστεί η καρδιακή δραστηριότητα και τα πρότυπα δραστηριότητας του συμπαθητικού νευρικού συστήματος/παρασυμπαθητικό νευρικό σύστημα. Οι μετρήσεις HRV θα πραγματοποιηθούν μέσω της συσκευής SEER 1000, ECG Recorder, General Electric (Version 1.0, 2067634-077 Revision F).

Πληροφορίες για διατροφικές συνήθειες και τρόπο ζωής

Τέλος, πληροφορίες που αφορούν τις διατροφικές συνήθειες, άλλες συνήθειες που

σχετίζονται με την υγεία και τον τρόπο ζώης, και ίσως επηρεάζουν τα επίπεδα μητρικής μελατονίνης, θα αξιολογηθούν (Peuhkuri, 2012).

Αξιολόγηση της ανάπτυξης των πρόωρων βρεφών

Στους 9 μήνες ζωής των βρεφών (διορθωμένη ηλικία), η κοινωνική και γνωστική τους ανάπτυξη θα αξιολογηθεί μέσω της χορήγησης του *Bayley Scales of Infant and Toddler Development, 3rd Edition* (Bayley, 2006), η οποία αποτελεί διαγνωστικό εργαλείο αξιολόγησης της ανάπτυξης για βρέφη και παιδιά ηλικίας από ενός έως 42 μηνών. Η Bayley-III αποτελείται από 5 κλίμακες: την κλίμακα για την αξιολόγηση της γνωστικής ανάπτυξης, την κλίμακα για την αξιολόγηση της γλωσσικής ανάπτυξης, την κλίμακα για την αξιολόγηση της κινητικής ανάπτυξης, την κλίμακα για την αξιολόγηση της κοινωνικο-συναισθηματικής ανάπτυξης καθώς και την κλίμακα για αξιολόγηση της προσαρμοστικής συμπεριφοράς.

Ζητήματα ηθικής και δεοντολογίας της έρευνας

Οι συμμετέχοντες της έρευνας λαμβάνουν μέρος στη μελέτη υπό τις αρχές της ενήμερης συγκατάθεσης και της πλήρους εμπιστευτικότητας. Κατόπιν της σχετικής ενημέρωσης των νέων μητέρων για την έρευνα και την ερευνητική διαδικασία (από τα μέλη της ερευνητικής ομάδας που θα είναι αρμόδια για την συλλογή των δεδομένων), οι μητέρες θα έχουν τη δυνατότητα να αποφασίσουν αβίαστα την συμμετοχή των ιδίων και του νεογνού τους στην έρευνα έχοντας πλήρη ενημέρωση για όσα συμπεριλαμβάνονται σε αυτή. Οι συμμετέχουσες μητέρες και τα νεογνά / βρέφη τους δεν πρόκειται να υποστούν οποιαδήποτε εξαπάτηση, βλάβη ή απώλεια και θα ληφθούν όλες οι προβλεπόμενες προφυλάξεις για τη διατήρηση της ευημερίας και της αξιοπρέπειας τους. Οι ίδιες διατηρούν το δικαίωμα να εγκαταλείψουν την έρευνα όποτε το θελήσουν καθώς και το δικαίωμα πρόσβασης στα ευρήματα της έρευνας (Willig, 2001).

Επιπλέον, το προτεινόμενο ερευνητικό έργο συμμορφώνεται πλήρως στις υποδείξεις Εθνικής Νομοθεσίας και του Εθνικού Δικαίου για τα ζητήματα που αφορούν τη διασφάλιση της προστασίας του απορρήτου των προσωπικών δεδομένων.

Στην προτεινόμενη διαχρονική μελέτη θα συμμετάσχουν μητέρες με τα νεογνά.βρέφη τους. Πιο συγκεκριμένα, τα μέλη της ερευνητικής ομάδας που θα είναι υπεύθυνα για την προσέγγιση των συμμετεχόντων και την συλλογή των δεδομένων θα ενημερώνουν τις νέες μητέρες αναλυτικά σχετικά με την έρευνα μέσω του Εντύπου Ενημέρωσης (επισυνάπτεται) για το στόχο της έρευνας, τη διαδικασία και τη διάρκεια συμμετοχής, τη διασφάλιση της ανωνυμίας και της εμπιστευτικότητας, τον εθελοντικό χαρακτήρα της συμμετοχής καθώς και την ευρύτερη ωφελιμότητα της έρευνας. Έτσι θα δίνεται η δυνατότητα σε κάθε μητέρα να επιλέξει αβίαστα την τυχόν συμμετοχή της στην έρευνα έχοντας πλήρη ενημέρωση για όσα συμπεριλαμβάνονται σε αυτή. Πριν την υπογραφή των σχετικών εντύπων συγκατάθεσης, οι ερευνήτριες θα προσφέρουν επιπλέον διευκρινήσεις και θα απαντούν σε τυχόν απορίες, συμπεριλαμβάνοντας σε κάθε περίπτωση την ενημέρωση για τα οφέλη από τη συμμετοχή στην έρευνα. Επιπλέον οι μητέρες θα ενημερωθούν με σχετική επιστολή για τη δυνατότητα υποβολής παραπόνων ή καταγγελιών στην Επιτροπή Ηθικής και Δεοντολογίας της Έρευνας του Πανεπιστημίου Κρήτης και στην Υπεύθυνη Επεξεργασίας Προσωπικών Δεδομένων του Πανεπιστημίου Κρήτης.

Μετά την αναλυτική παρουσίαση της ερευνητικής διαδικασίας και την παροχή απαντήσεων σε τυχόν ερωτήματα, θα ληφθεί έγγραφη ενήμερη συγκατάθεση για την εθελοντική συμμετοχή των μητέρων σύμφωνα με τους όρους της Διακήρυξης του Helsinki.

Ακολούθως, θα ζητηθεί από τις μητέρες που θα έχουν συμφωνήσει να λάβουν μέρος στην έρευνα:
α) να συναινέσουν στη μέτρηση της μελατονίνης σε αίμα που θα έχει ληφθεί από τον ομφάλιο λώρο (opt-out approach). Στην περίπτωση της μη-συναίνεσης, το αντίστοιχο δείγμα θα καταστρέφεται,
β) να συναινέσουν στη μέτρηση μελατονίνης σε αίμα του νεογνού κατά τη νοσηλεία του (το αίμα θα προέρχεται από αιμοληψίες ρουτίνας),
γ) να προσκομίσουν 3 δείγματα μητρικού γάλατος σε συγκεκριμένες χρονικές περιόδους (βλ. παραπάνω) με σκοπό τη μέτρηση των επιπέδων μελατονίνης,
δ) να συμπληρώσουν μια συστοιχία ερωτηματολογίων για την αξιολόγηση διαφόρων ψυχοκοινωνικών παραγόντων στις τρείς καθορισμένες χρονικές περιόδους (κατά τη γέννηση, 6 και 9 μήνες),
ε) να συναινέσουν στην αξιολόγηση της μεταβλητότητας του καρδιακού ρυθμού των νεογνών στις 2 καθορισμένες χρονικές περιόδους και των μητέρων [κατά την πρώτη εβδομάδα ζωής και στους 9 μήνες (διορθωμένη ηλικία)],
στ) να συναινέσουν στην νευροαναπτυξιακή αξιολόγηση των βρεφών τους μέσω της χορήγησης του *Bayley Scales of Infant and Toddler Development, 3rd Edition* (Bayley, 2006) σε διορθωμένη ηλικία 9 μηνών.

Η συμπλήρωση των ερωτηματολογίων θα γίνει είτε δια ζώσης είτε διαδικτυακά μέσω ειδικής πλατφόρμας. Ο σύνδεσμος που οδηγεί στην ειδική πλατφόρμα θα αποσταλεί στην ηλεκτρονική διεύθυνση των μητέρων η οποία θα έχει δηλωθεί από τις ίδιες κατά την έγγραφη ενήμερη συγκατάθεση.Τα έντυπα ενημέρωσης και συγκατάθεσης και η λίστα συμμετεχόντων/κωδικών θα φυλλάσονται σε κλειδωμένο ερμάριο στο γραφείο της Επιστημονικά Υπεύθυνης στο Τμήμα Ψυχολογίας του Πανεπιστημίου Κρήτης για περίοδο 7 ετών και μετά θα καταστραφούν με έναν καταστροφέα εγγράφων. Τα ψηφιακά δεδομένα αφού καταχωρηθούν στη βάση δεδομένων, διαγράφονται μόνιμα.

Τα δεδομένα της μελέτης θα αποθηκευτούν σε ειδικά υπολογιστικά φύλα ανώνυμα. Πιο συγκεκριμένα, για την μεγιστοποίηση της ανωνυμοποίησης του φυσικού και του ψηφιακού υλικού που θα προέλθει κατά τη φάση της συλλογής δεδομένων αρχικά στον κάθε συμμετέχοντα θα δοθεί κωδικός που θα περιέχει γράμματα ή/και αριθμούς. Ο κωδικός αυτός θα προέλθει από την τυχαιοποιημένη ανάμειξη των γραμμάτων/αριθμών μέσα από διαδικασία ηλεκτρονικής κλήρωσης. Επίσης, κατά την ίδια φάση, ο κάθε κωδικός θα συμπληρωθεί από ένα χαρακτηριστικό το οποίο θα υποδηλώνει την αντιστοίχιση του/της κάθε συμμετέχοντα/συμμετέχουσας με την οικογένεια από την οποία προέρχεται. Ομοίως με τους κωδικούς για κάθε συμμετέχοντα, τα χαρακτηριστικά αντιστοίχησης θα περιέχουν γράμματα ή/και αριθμούς και θα προκύψουν από την τυχαιοποιημένη ανάμειξή τους μέσα από διαδικασία ηλεκτρονικής κλήρωσης. Η αντιστοίχιση συμμετέχοντα με κωδικό θα γίνει μια φορά στη διάρκεια της μελέτης. Κανείς από τους ερευνητές που θα διαχειρίζονται τα δεδομένα δεν θα έχει πρόσβαση στα ονόματα των συμμετεχόντων/συμμετεχουσών και μόνο η Επιστημονικά Υπεύθυνη (ΕΥ) θα μπορεί να επανασυνδέσει τα φυλαγμένα ονόματα με τους κωδικούς τους, αν χρειαστεί. Το κλειδί που θα αντιστοιχεί το κώδικα με το όνομα γονέα θα το γνωρίζει μόνο η Επιστημονικά Υπεύθυνη της μελέτης και θα βρίσκεται φυλαγμένο σε χώρο στο γραφείο της ΕΥ στο Τμήμα Ψυχολογίας του Πανεπιστημίου Κρήτης (Ρέθυμνο) (η Πανεπιστημιούπολη Ρεθύμνου φυλάσσεται από ειδικό προσωπικό).

Επιπλέον, όλες οι πληροφορίες και τα δεδομένα που θα συλλεχθούν κατά τη διάρκεια του προτεινόμενου έργου θα διατηρηθούν με πλήρη εχεμύθεια. Ειδικότερα, όλοι οι τύποι ηλεκτρονικών αρχείων (όπως οι βάσεις δεδομένων, τα υπολογιστικά φύλλα κ.λπ.)

που περιέχουν αναγνωρίσιμες πληροφορίες θα φέρουν ειδική κωδικοποίηση με τέτοιο τρόπο ώστε να μην είναι δυνατόν να αποκαλυφθεί η ταυτότητα των συμμετεχόντων. Οι κωδικοί αυτοί θα φυλάσσονται σε ξεχωριστό ερμάριο από εκείνον που φυλλάσονται τα παραπάνω κλειδιά αντιστοίχισης στο γραφείο της ΕΥ στο Τμήμα Ψυχολογίας του Πανεπιστημίου Κρήτης. Στα δεδομένα φυσικού και ψηφιακού αρχείου θα έχουν πρόσβαση αποκλειστικά και μόνο τα μέλη της ερευνητικής ομάδας του προγράμματος. Οποιοσδήποτε υπολογιστής φιλοξενεί τα παραπάνω αρχεία θα έχει επίσης προστασία με κωδικό πρόσβασης και δεν θα είναι προσβάσιμος μέσω διαδικτύου.

Τα μέλη της ερευνητικής ομάδας που θα συμβάλλουν στην προσέγγιση των συμμετεχόντων και τη συλλογή των δεδομένων θα ενημερώσουν εξ αρχής τους συμμετέχοντες για την ασφάλεια και την εθελοντική συμμετοχή τους στην έρευνα σύμφωνα με τους όρους της Διακήρυξης του Helsinki. Σε περίπτωση που προκύψουν θέματα που αφορούν την ασφάλεια των συμμετεχόντων η Επιστημονικά Υπεύθυνη του προτεινόμενου έργου θα επικοινωνήσει και θα συζητήσει με τους συμμετέχοντες για την επιθυμία τους να συνεχίσουν, ή να διακόψουν τη συμμετοχή τους στη μελέτη. Στην περίπτωση που οι συμμετέχοντες αποφασίσουν να διακόψουν τη συμμετοχή τους και να αποχωρήσουν από το δείγμα, η Επιστημονικά Υπεύθυνη του έργου σε συνεργασία με τα μέλη της ερευνητικής ομάδας θα προβούν στην καταστροφή των προσωπικών δεδομένων των συγκεκριμένων συμμετεχόντων. Σε περίπτωση που θα διαπιστωθεί ότι κατά τη διάρκεια της έρευνας εκδηλώνεται οποιαδήποτε συμπτωματολογία στην ψυχική υγεία της μητέρας που χρήζει υποστήριξης από κάποιο ειδικό ψυχικής υγείας, υπάρχει η πρόβλεψη να διακοπεί η συμμετοχή στην έρευνα και να παραπεμφθεί σε σχετική δομή/δημόσια υπηρεσία του νομού στον οποίο κατοικεί, ή στην πλησιέστερη στην κατοικία της υγειονομική δομή η μητέρα στη σχετική δομή.

Υπό όλες τις παραπάνω προϋποθέσεις θεωρούμε ότι το προτεινόμενο ερευνητικό πρόγραμμα δεν διέπεται από οποιουσδήποτε ηθικούς ή δεοντολογικούς προβληματισμούς.

Διαδικασία υποβολής παραπόνων ή καταγγελιών

Για οποιαδήποτε παράπονα ή καταγγελίες σχετικά με τη διεξαγωγή της έρευνας οι συμμετέχουσες μπορούν να προσφύγουν στην Επιτροπή Ηθικής και Δεοντολογίας του Πανεπιστημίου Κρήτης, ehde@uoc.gr, και στην Επιτροπή Ηθικής και Δεοντολογίας του Πανεπιστημιακού Γενικού Νοσοκομείου Κρήτης, researchprot@pagni.gr

Για οποιαδήποτε καταγγελία σχετικά με τη διαχείριση των προσωπικών δεδομένων των συμμετεχόντων, οι ίδιοι μπορούν να απευθυνθούν στην Υπεύθυνη Επεξεργασίας Προσωπικών Δεδομένων του Πανεπιστημίου Κρήτης, κα **Έλπίδα Βαμβακά,** email:dpo@uoc.gr και σε κάθε περίπτωση στην Αρχή Προστασίας Δεδομένων Προσωπικού Χαρακτήρα complaints@dpa.gr.

Όλα τα μέλη της ερευνητικής ομάδας θα εκπαιδευτούν ως προς τη διαχείριση των δεδομένων, την προστασία των προσωπικών δεδομένων, την ανωνυμία και την εμπιστευτικότητα των προσωπικών φακέλων.

Αιτιολόγηση για το μέγεθος του δείγματος και στατιστική ανάλυση

Οι υπολογισμοί ισχύος θα εξεταστούν βασιζόμενοι σε επίπεδο σημαντικότητας 5% και 80% για την ισχύ. Τα αποτελέσματα της μελέτης θα βασιστούν σε ένα μέγεθος δείγματος τουλάχιστος N = 100 δυάδες μητέρας-βρέφους, προκειμένου να μπορέσουμε να ανιχνεύσουμε μια διαφορά τουλάχιστον 10 μονάδων.

Αρχικά, διερευνητική ανάλυση θα διεξαχθεί προκειμένου να κατανοήσουμε τις κατανομές των μεταβλητών, να γίνουν οι σχετικές προσαρμογές, να κατανοήσουμε τις πιθανές συσχετίσεις, και για να αναγνωρίσουμε τις ακραίες παρατηρήσεις. Για τη διερεύνηση των επιμέρους στόχων της μελέτης, θα χρησιμοποιήσουμε Generalized Additive Models (GAMs) και Structural Equation Modeling (SEM), ενώ η συσχέτιση των ψυχοκοινωνικών και βιολογικών παραγόντων με τα αναπτυξιακά αποτελέσματα των πρόωρων βρεφών στους 9 μήνες (διορθωμένη ηλικία), συγκρίνοντας οικογένειες χαμηλού και μέσου/υψηλού κοινωνικοοικονομικού επιπέδου, θα γίνει εφαρμόζοντας τεχνικές linear regression modeling και generalized linear modeling.

References

Adhikari, K., Patten, S.B., Williamson, T., *et al.* (2020). Neighbourhood socioeconomic status modifies the association between anxiety and depression during pregnancy and preterm birth: a Community-based Canadian cohort study. *BMJ Open*, 10(2)

Ask, T.F., Ranjitkar, S., Ulak, M., *et al.* (2019). The association between heart rate variability and neurocognitive and socio-emotional development in Nepalese infants. *Frontiers in Neuroscience*, 13:411.

Bayley, N. (2006). *Bayley Scales of Infant and Toddler Development,* Third Edition: Pearson.

Barker, D. J. (1998). In utero programming of chronic disease. *Clinical Science*, 95(2), 115-128.

Barker, D. J. (2002). Fetal programming of coronary heart disease. *Trends in Endocrinology Metabolism*, 13, 364-368.

Barker, D. J. (2007). The origins of the developmental theory. *Journal of Internal Medicine*, 261, 412-417.

Blount, A.J., Adams, C.R., Anderson-Berry, A.L., *et al.* (2021). Biopsychosocial Factors during the Perinatal Period: Risks, Preventative Factors, and Implications for Healthcare Professionals. *International Journal of Environmental Research and Public Health*, 18(15):8206.

Bodenmann, G. (1995). A systemic-transactional conceptualization of stress and coping in couples. *Swiss Journal of Psychology / Schweizerische Zeitschrift für Psychologie / Revue Suisse de Psychologie, 54*(1), 34–49.

Bodenmann, G. (2005). Dyadic coping and its significant for marital functioning. In T. Revenson, K. Kayser, & G. Bodenmann (Eds.), *Couples coping with stress: Emerging perspectives on dyadic coping* (pp. 33-50). APA.

Braam, W., Ehrhart, F., Maas, A.P.H.M., *et al.* (2018). Low maternal melatonin level increases autism spectrum disorder risk in children. *Research in Developmental Disabilities*, 82:79-89.

Brown, L. (2007). Heart rate variability in premature infants during feeding. *Biological Research for Nursing*, 8(4).

Biran, V., Decobert, F., Bednarek, N., *et al.*(2019). Melatonin levels in preterm and term infants and their mothers. *International Journal of Molecular Sciences*, 20(9):2077

Carrulo, J., Justo, J.M.R.M., Figueiredo, B. (2022). Maternal perception of infant's intersubjectivity: a questionnaire. *Journal of Reproductive and Infant Psychology*, 1-11.

Cena, L., Mirabella, F., Palumbo, G., *et al.* (2021). Prevalence of maternal antenatal and postnatal depression and their association with sociodemographic and socioeconomic factors: A multicentre study in Italy. *Journal of Affective Disorders*, 279, 217-221.

Chaudron, L.H., Szilagyi, P.G., Tang, W., *et al.* (2010). Accuracy of depression screening tools for identifying postpartum depression among urban mothers. *Pediatrics*, 125(3): e609-17.

Clements, K.M., Barfield, W.D., Ayadi, M.F., *et al.* (2007). Preterm birth-associated cost of early intervention services: an analysis by gestational age. *Pediatrics*, 119(4):e866-74.

Coe, J.L., Davies, P.T., Sturge-Apple, M.L. (2018). Family cohesion and enmeshment moderate associations between maternal relationship instability and children's externalizing problems. *Journal of Family Psychology*, 32(3):289-298.

Cohen Engler A, Hadash A, Shehadeh N, *et al.* (2012). Breastfeeding may improve nocturnal sleep and reduce infantile colic: potential role of breast milk melatonin. *European Journal of Pediatrics*,

171(4), 729-32.
Condon, J. T., & Corkindale, C. J. (1996). The assessment of parent-to-infant attachment: Development of a self-report questionnaire instrument *Journal of Reproductive and Infant Psychology,* 16:1, 57-76.
Conger, R.D., Conger, K.J., Martin, M.J. (2010). Socioeconomic status, family processes, and individual development. *Journal of Marriage and Family*, 72(3):685-704.
Coussons-Read, M.E. (2013). Effects of prenatal stress on pregnancy and human development: mechanisms and pathways. Obstetric Medicine , 6(2):52-57.
Cox, J. L., Holden, J. M., Sagovsky, R. (1987). Detection of postnatal depression: development of the 10-item Edinburgh Postnatal Depression Scale. *The British Journal of Psychiatry*, *150*(6), 782-786.
D'Angelo, G., Chimenz., R, Reiter., R.J., *et al.* (2020). Use of Melatonin in Oxidative Stress Related Neonatal Diseases. *Antioxidants*, 9(6):477.
Dierckx, B., et al. (2009). Maternal psychopathology influences infant heart rate variability: Generation R study. *Psychosomatic Medicine, 71*, 313-321.
Doussard-Roosevelt, J. A., McClenny, B. D., Porges, S. W. (2001). Neonatal cardiac vagal tone and school- age developmental outcome in very low birth weight infants. *Developmental Psychobiology: The Journal of the International Society for Developmental Psychobiology, 38*(1), 56-66.
Doussard-Roosevelt J, Porges S, Scanlon J, *et al.* (1997). Vagal regulation of heart rate in the prediction of developmental outcome for very low birth weight preterm infants. *Child Development, 68*, 173–86.
Enlow, E., Faherty, L.J, Wallace-Keeshen, S., *et al.* (2017). Perspectives of low socioeconomic status mothers of premature infants. *Pediatrics*, 139(3): e20162310.
Eriksson, J. G. (2016). Developmental origins of health and disease – from a small body size at birth to epigenetics. *Annals of Medicine*, 48 (6), 456-467.
Falconier, M. K., Jackson, J. B., Hilpert, P., *et al.* (2015). Dyadic coping and relationship satisfaction: A meta-analysis. *Clinical Psychology Review*, *42*, 28–46.
Field, T., Diego, M. (2008). Vagal activity, early growth and emotional development. *Infant Behavior and Development, 31*(3), 361-373.
Field, T., Pickens, J., Fox, A. N., *et al.* (1995). Vagal tone in infants of depressed mothers. *Developmental Psychopathology, 7*(2), 227-231.
Fisher, J., Cabral de Mello, M., Patel, V., *et al.* (2012). Prevalence and determinants of common perinatal mental disorders in women in low- and lower-middle-income countries: a systematic review. *Bulletin of the WHO*, 90(2)
Florian, S., Ichou, M., Panico, L. (2021). Parental migrant status and health inequalities at birth: The role of immigrant educational selectivity. *Social Science & Medicine*, 278:113915
Forcada-Guex M, Pierrehumbert B, Borghini A, *et al.* (2006). Early dyadic patterns of mother-infant interactions and outcomes of prematurity at 18 months. *Pediatrics*, 118(1):e107-14.
Giannakakis, G., Marias, K., & Tsiknakis, M. (2019). A stress recognition system using HRV parameters and machine learning techniques, 8th International Conference on Affective Computing and Intelligent Interaction Workshops and Demos (ACIIW), pp. 269-272.
Giannakou, M., Roussi, P., Kosmides, M. E., *et al.* (2013). Adaptation of the beck depression inventory-II to greek population. *Hellenic Journal of Psychology, 10*, 120-146.
Giannopoulou, I., Tsobanoglou, G.O. (2020). COVID-19 pandemic: challenges and opportunities for the Greek health care system. *Irish Journal of Psychological Medicine*, 37(3):226-230.
Gluckman, P. D. Hanson, M. A. (2006). The developmental origins of health and disease: The breadth and importance of the concept. In Wintour E. M. & Owes, J. A. (Eds), Early Life Origins of Health and Disease. Springer Media.
Ferber, S.G., Als, H., McAnulty, G., Peretz, H., *et al.* (2011). Melatonin and mental capacities in newborn infants. *Journal of Pediatrics*, 159(1):99-103.e1.
Gombert, M., Codoñer-Franch, P. (2021). Melatonin in early nutrition: Long-Term effects on cardiovascular system. *International Journal of Molecular Sciences*, 22(13):6809.
Gonzalez-Gomez, N., O'Brien, F., Harris, M. (2020). The effects of prematurity and socioeconomic depreivation on early speech perception: A story of two different delays. *Developmental Science*, 24(2): e 13020.
Goyal, D., Gay, C., Lee, K.A. (2010). How much does low socioeconomic status increase the risk of prenatal and postpartum depressive symptoms in first-time mothers? *Womens Health Issues*, 20(2): 96-104.
Graham, A., Ablow, J. C., Measelle, J. R. (2010). Interparental relationship dynamics and cardiac vagal functioning in infancy. *Infant Behavior and Development, 33*, 530-544.
Graziano, P., Derefinko, K. (2013). Cardiac vagal control and children's adaptive functioning: A meta-

analysis. *Biological Psychology, 94*, 22-37.
Groër, M., Davis, M., Casey, K., *et al.* (2005). Neuroendocrine and immune relationships in postpartum fatigue. *MCN: The American Journal of Maternal/ Child Nursing,* 30(2):133-8.
Hoffenkamp, H.N., Tooten, A., Hall, R.A., *et al.* (2012). The impact of premature childbirth on parental bonding. *Evolutionary Psychology,* 17;10(3):542-61
Howard, L.M., Khalifeh, H. (2020). Perinatal mental health: a review of progress and challenges. *World Psychiatry,* 19(3):313-327.
Huang, Y., Liu, Y., Wang, Y. *et al.* (2021) Family function fully mediates the relationship between social support and perinatal depression in rural Southwest China. *BMC Psychiatry* **21**, 151.
Hurt, H., Betancourt, L.M. (2017). Turning 1 year of age in a low socioeconomic environment: A portrait of disadvantage. *Journal of Developmental and Behavioral Pediatrics*, 38(7):493-500.
Hosokawa, R., Katsura, T. (2018). Role of parenting style in children's behavioral problems through the transition from preschool to elementary school according to gender in Japan. *International Journal of Environmental Research and Public Health*, 16(1):21.
Italianer, M.F., Naninck, E.F.G., Roelants, J.A., *et al.* (2020). Circadian variation in human milk composition, a systematic review. *Nutrients*, 12(8):2328.
Jansen, L., Peeters-Scholte, C.M.P.C.D., van den Berg-Huysmans, A. A., *et al.* (2021) Longitudinal Follow-Up of Children Born Preterm: Neurodevelopment From 2 to 10 Years of Age. *Frontiers in Pediatrics*, 9:674221.
Javorka, K., Lehotska, Z., Kozar, M., *et al.* (2017). Heart rate variability in newborns. *Physiological Research, 66* (Suppl. 2), S203-S214.
Johnson S, Marlow N. (2011). Preterm birth and childhood psychiatric disorders. *Pediatric Research*, 69(5 Pt 2):11
Jones, N. A., Field, T., Fox, N. A., *et al.* (1998). Newborns of mothers with depressive symptoms are physiologically less developed. *Infant Behavior and Development, 21* (3), 537-541.
Katzer, D., Pauli, L., Mueller, A. *et al.* (2016). Melatonin Concentrations and Antioxidative Capacity of Human Breast Milk According to Gestational Age and the Time of Day. *Journal of Human Lactation*, 32(4):NP105-NP110.
Korja, R, Latva, R., Lehtonen, L. (2012). The effects of preterm birth on mother-infant interaction and attachment during the infant's first two years. *Acta Obstetricia et Gynecologica Scandinavica*, 91(2):164-73.
Koutra, K., Chatzi, L., Roumeliotaki T., *et al.* (2012). Socio-demographic determinants of infant neurodevelopment at 18 months of age: Mother-Child Cohort (Rhea Study) in Crete, Greece. *Infant Behavior and Development*, 35(1):48.
Kozhimannil, K.B., Trinacty, C.M., Busch, A.B., *et al.* (2011). Racial and ethnic disparities in postpartum depression care among low-income women. *Psychiatric Services,* 62(6):619-25.
Kwong, A.K., Boyd, R.N., Chatfield, M.D., *et al.* (2022). Early Motor Repertoire of Very Preterm Infants and Relationships with 2-Year Neurodevelopment. *Journal of Clinical Medicine*, 25;11(7):1833.
Koutra, K., Triliva, S., Roumeliotaki, *et al.* (2013). Cross-cultural adaptation and validation of the Greek version of the family adaptability and cohesion evaluation scales IV package (FACES IV Package). *Journal of Family Issues, 34*(12), 1647-1672.
Lavanga, M., Heremans, E., Moeyersons, J., *et al.* (2021). Maturation of the Autonomic Nervous System in Premature Infants: Estimating Development Based on Heart-Rate Variability Analysis. *Frontiers in Physiology,* 11:581250.
Leahy-Warren, P., Coleman, C., Bradley, R. *et al.* (2020) The experiences of mothers with preterm infants within the first-year post discharge from NICU: social support, attachment and level of depressive symptoms. *BMC Pregnancy Childbirth,* 20, 260.
Liakos, A., Giannitsi, S. (1984). Reliability and validity of the modified Greek version of the Spielberger State-Trait Anxiety Inventory. *Encephalos*, 21, 71-76.
LoBue, V., Reider, L.B., Kim, E., *et al.* (2020). The importance of using multiple outcome measures in infant research. *Infancy*, 25(4):420-437.
Longin, E., Gerstner, T., Schaible, T., *et al.*.(2006). Maturation of the autonomic nervous system: differences in heart rate variability in premature vs. term infants. *Journal of Perinatal Medicine*, 34(4):303-8.
Lutkiewicz, K. (2020). Social Support, Perceived Stress, Socio-Demographic Factors and Relationship Quality among Polish Mothers of Prematurely Born Children. *Int Journal of Environmental Research and Public Health,* 17(11).
Malow, B.A., Adkins, K.W., Reynolds, A., *et al.* (2014). Parent-based sleep education for children with autism spectrum disorders. *Journal of Autism and Developmental Disorders,* 44(1):216-28.

Mandy, M., Nyirenda, M. (2018). Developmental Origins of Health and Disease: the relevance to developing nations. *International Health*, 10(2):66-70.

Maurer, F. A., Smith, C. M. (2013). Community/public health nursing practice: health for families and populations (5th ed.). Elsevier/ Saunders.

M'hamdi, H. I., Beaufort, I., Jack, B. W., *et al.* (2017). Responsibility in the age of Developmental Origins of Health and Disease (DOHaD) and epigenetics. *Journal of Developmental Origins of Health and Disease*, 9(1): 1-5.

Moore, G. A. (2010). Parent conflict predicts infants' vagal regulation in social interaction. *Developmental Psychopathology, 22*(1), 23-33.

Mulkey, S. B., du Plessis, A. J. (2019). Autonomic system development and its impact on neuropsychiatric outcome. *Pediatric Research, 85*(2), 120-126

Ngai, F.W., Ngu, S.F. (2014). Family sense of coherence and family adaptation among childbearing couples. *Journal of Nursing Scholarship*, 46(2):82-90.

Nobile, S., Di Sipio Morgia, C., Vento, G. (2022). Perinatal origins of adult disease and opportunities for health promotion: A narrative review. *Journal of Personalized Medicine*, 12(2):157.

Olson, D.H., Sprenkle, D.H., Russell, C.S. (1979). Circumplex model of marital and family systems: I. Cohesion and adaptability dimensions, family types, and clinical applications. *Family Process, 18*(1), 3-28.

Olson, D. H., Waldvogel, L., Schlieff, M. (2019). Circumplex Model of marital and family systems: An update. *Journal of Family Therapy and Review, 11*, 199-211.

Olson, D. H. (2000).Circumplex model of marital and family systems. *Journal of Family Therapy*, *22*(2), 144-167.

Panceri, C., Valentini, N.C., Silveira, R.C., *et al.* (2020). Neonatal adverse outcomes, neonatal birth risks, and socioeconomic status: Combined influence on preterm infants' cognitive, language, and motor development in Brazil. *Journal of Child Neurology*, 35(14):989-998.

Papadakaki, M., Stamouli, M-A., Chliaoutakis, J. (2021). Exploring the psychosocial needs of people living in extreme poverty and introducing brief interventions: The case of Crete region in Greece. *Research on Social Work Practice*, 3(4), 410-420.

Parker, S.J., Zahr, L.K., Cole, J.G., *et al.* (1992). Outcome after developmental intervention in the neonatal intensive care unit for mothers of preterm infants with low socioeconomic status. *Journal of Pediatrics*, 120(5):780-5.

Parlapani, E., Holeva, V., Voitsidis, P., *et al.* (2020) Psychological and Behavioral Responses to the COVID-19 Pandemic in Greece. *Frontiers in Psychiatry*, 11:821.

Peuhkuri, K., Sihvola, N., & Korpela, R. (2012). Dietary factors and fluctuating levels of melatonin. *Food and Nutrition Research*, 56: 17252.

Porges, S. W., & Furman, S. A. (2011). The early development of the autonomic nervous system provides a neural platform for social behavior: A polyvagal perspective. *Infant and Child Development*, *20*(1), 106-118.

Pizzi, C., Richiardi, M., Charles., M-A., *et al*. (2020). Measuring child socio-economic position in birth cohort research: The development of a novel standardized household income indicator. *International Journal of Environmental Research and Public Health*, 17(5), 1700.

Porter, C. L., Wouden-Miller, M., Silva, S. S., *et al.* (2003) Marital harmony and conflict: Linked to infants' emotional regulation and cardiac vagal tone. *Infancy*, *4*(2), 297-307.

Potijk, M.R., de Winter, A.F., Bos, A.F., *et al.* (2012). Higher rates of behavioural and emotional problems at preschool age in children born moderately preterm. *Archives of Disease in Childhood*, 97(2):112-7.

Pravia, C.I., Benny, M. (2020). Long-term consequences of prematurity. *Cleveland Clinic Journal of Medicine*, 23;87(12):759-767.

Prom, M.C., Denduluri, A., Philpotts, L.L., *et al.* (2022). A Systematic Review of Interventions That Integrate Perinatal Mental Health Care Into Routine Maternal Care in Low and Middle-Income Countries. *Frontiers in Psychiatry* 13:859341.

Provenzi, L., Borgatti, R., Montirosso, R. (2017). Why are prospective longitudinal studies needed in preterm behavioral epigenetic research? *JAMA Pediatrics*, 171(1), 92.

Qin, Y., Shi, W., Zhuang, J. *et al.* (2019) Variations in melatonin levels in preterm and term human breast milk during the first month after delivery. *Scientific Reports*, 9, 17984.

Reiss, F., Meyrose, A.K., Otto, C., *et al.* (2019). Socioeconomic status, stressful life situations and mental health problems in children and adolescents: Results of the German BELLA cohort-study. *PLoS One*, 14(3):e0213700.

Roussi, P., Karademas, E. C. (2016). Dyadic coping in Greek couples. *Dyadic Coping: International Perspectives*, 153.

Samuel, T.M., Zhou, Q., Giuffrida, F., *et al.* (2020) Nutritional and non-nutritional composition of human milk Is modulated by maternal, infant, and methodological factors. *Frontiers in Nutrition,* 7:576133.

Scorza, P., Monk, C., Lee, S., *et al.* (2020). Preventing maternal mental health disorders in the context of poverty: pilot efficacy of a dyadic intervention. *American Journal of Obstetrics and Gynecology MFM*, 2(4):100230.

Shigeto, A., Mangelsdorf, S. C., Brown, G. J. (2013). Roles of family cohesiveness, marital adjustment and child temperament in predicting child behavior with mothers and fathers. *Journal of Social and Personal Relationships*, 31(2), 200-220.

Singer, L.T., Fulton, S., Davillier, M., *et al.* (2003). Effects of infant risk status and maternal psychological distress on maternal-infant interactions during the first year of life. *Journal of Developmental and Behavioral Pediatrics,* 24(4), 233–241.

Singletary, B., Bates, R., Justice, L. (2021). Evaluating associations between maternal social support and cognitive development for infants in poverty. *Infant Behavior and Development*, 63: 101546

Spielberger, C.D., Gorsuch, R.L., Lushene, R. (1983) State-Trait Anxiety Inventory for Adults: Sampler Set, Manual, Instrument and Scoring Guide. Consulting Psychologists Press, Palo Alto, CA, 1983.

Suga, A., Uraguchi, M., Tange, A., *et al.* (2019). Cardiac interaction between mother and infant: enhancement of heart rate variability. *Scientific Reports, 9*(1), 1-9.

Sullivan, M.C., Winchester, S.B., Bryce, C.I., *et al.* (2017). Prematurity and perinatal adversity effects hypothalamic-pituitary-adrenal axis reactivity to social evaluative threat in adulthood. *Developmental Psychobiology,* 59(8):976.

Tauman, R., Zisapel, N., Laudon, M., *et al.* (2002). Melatonin production in infants. *Pediatric Neurology,* 26(5):379-82.

Theofilou, P. (2015). Translation and cultural adaptation of Multidimensional Scale of Perceived Social Support for Greece. *Health Psychology Research, 3*(1061), 9-11.

Tissot, H., Lapalus, N., Frascarolo, F. *et al.* (2022). Family alliance in infancy and toddlerhood predicts social cognition in adolescence. *Journal of Child and Family Studies*, 31, 1338-1349.

Tordjman, S., Chokron, S., Delorme, R., *et al.* (2017). Melatonin: Pharmacology, functions and therapeutic benefits. *Current Neuropharmacology*, 15(3):434-443.

Trevarthen, C. (2001). Intrinsic motives for companionship in understanding: Their origin, development, and significance for infant mental health. *Infant Mental Health Journal*, *22*(1–2), 95–131.

Tripathy, P. (2020). A public health approach to perinatal mental health: Improving health and wellbeing of mothers and babies. *Journal of Gynecology, Obstetrics and Human Reproduction*, 49(6):101747.

Tsobanoglou, G.O. (2014). The Eurozone crisis: social factors and impacts, emergent sociality and community employment capacity building. In N.P. Petropoulos, & G.O. Tsobanoglou (Eds.), *The Debt Crisis in the Eurozone: Social Impacts* (pp. 396–424). Cambridge Scholars Publishing.

Van Haeken, S., Braeken, M.AK.A., Nuyts, T., *et al.* (2020). Perinatal Resilience for the First 1,000 Days of Life. Concept Analysis and Delphi Survey. *Frontiers in Psychology*, 11:563432.

Van der Hulst, M., Polinder, S., Kok, R., *et al.* (2022). Socio-economic determinants of healthcare costs in early life: a register-based study in the Netherlands. *International Journal of Equity in Health*, 21(1):5.

Van der Merwe, S.E., Biggs, R., Preiser, R., *et al.* (2019). Making Sense of Complexity: Using SenseMaker as a Research Tool. *Systems*, *7*, 25.

Vatavali, F.; Gareiou, Z.; Kehagia, F.; *et al.* (2020). Impact of COVID-19 on Urban Everyday Life in Greece. Perceptions, Experiences and Practices of the Active Population. *Sustainability, 12*, 9410.

Vavoura, C., Vavouras, I. S. (2022). Income inequality and poverty in Greece during the recent economic, fiscal and Covid-19 crises. *Social Cohesion and Development*, 17(1), 5-21.

Vivilaki, V.G., Dafermos, V., Kogevinas, M., *et al.* (2009). The Edinburgh Postnatal Depression Scale: translation and validation for a Greek sample. *BMC Public Health*, 9:329.

Vlachadis, N., Kornarou, E., Ktenas, E. (2013). The preterm births epidemic in Greece. *Acta Obstetricia et Gynecologica Scandinavica*, 92(10):1231.

Weyers, S., Dragano, N., Möbus, S. *et al.* (2008). Low socio-economic position is associated with poor social networks and social support: results from the Heinz Nixdorf Recall Study. *International Journal of Equity in Health,* 7, 13.

Widding, U.; Farooqi, A. (2016). I thought he was ugly: Mothers of extremely premature children narrate their experiences as troubled subjects. *Feminism and Psychology*, 26, 153–169.

Wille, D. E (1991). Relation of preterm birth with quality of infant-mother attachment at one year.

Infant Behavior and Development, 14, 227-240.

Willig, C. (2001). *Introducing Qualitative Research in Psychology. Adventures in theory and method.* United Kingdom: Open University Press.

Winstone LK, Luecken LJ, Crnic KA, *et al.* (2020). Patterns of family negativity in the perinatal period: Implications for mental health among Mexican-origin women. *Journal of Family Psychology*, 34(5):642-651.

Wong, H.S., Edwards, P. (2013). Nature or nurture: a systematic review of the effect of socio-economic status on the developmental and cognitive outcomes of children born preterm. *Maternal and Child Health Journal*, 17(9):1689.

Youngblut, J.M., Loveland-Cherry, C.J., Horan, M. (1994). Maternal employment effects on families and preterm infants at 18 months. *Nursing Research*, 43(6):331-7.

Zimet, G.D., Dahlem, N.W., Zimet, S.G., *et al.* (1988). The Multidimensional Scale of Perceived Social Support. *Journal of Personality Assessment*, *52*(1), 30-41.
