# Supplementary material for: The development of preterm infants from low socio-economic status families: The combined effects of melatonin, autonomic nervous system maturation and psychosocial factors (ProMote): A study protocol
Source: PLoS One. 2025 Jan 10;20(1):e0316520. doi: 10.1371/journal.pone.0316520 (PMC11723634; doi:10.1371/journal.pone.0316520)
Supplement: S2 File — (PDF) [file pone.0316520.s002.pdf]

RESEARCH PROTOCOL

(KA 11455)

RESEARCH PROJECT TITLE

The Development of Preterm Infants from Low Socio-Δconomic Status Families: The Combined Effects of Melatonin, Autonomic Nervous System Maturation and Psychosocial Factors (ProMote)

RESEARCH PROJECT FUNDING

The research project entitled: “The Development of Preterm Infants from Low Socio-Economic Status Families: The Combined Effects of Melatonin, Autonomic Nervous System Maturation and Psychosocial Factors (ProMote)”, is implemented in the framework of H.F.R.I call “Basic research Financing (Horizontal support of all Sciences)” under the National Recovery and Resilience Plan “Greece 2.0” funded by the European Union –NextGeneration EU (H.F.R.I. Project Number: 15730).

RESEARCH TEAM

Theano Kokkinaki (Scientific Responsible), Professor of Developmental Psychology, Department of Psychology, Laboratory of Applied Psychology, Unit for Child Development and Education, University of Crete, Gallou Campus, Rethymnon 74 150

Eleftheria Hatzidaki, Assistant Professor of Neonatology, School of Medicine, University of Crete, Director of the Neonatology Department and Neonatal Intensive Care Unit, University General Hospital of Heraklion, University of Crete

Aristides Tsatsakis, Professor of Toxicology, School of Medicine, University of Crete, School of Medicine, University of Crete

Emmanuel Tzatzarakis, Associate Professor of Toxicology, School of Medicine, University of Crete, School of Medicine, University of Crete

Eleni Vakonaki, Biologist, Researcher, Toxicology Laboratory, School of Medicine, University of Crete

Theano Roumeliotaki, Statistician, Clinic of Preventive Medicine and Nutrition, Department of Social Medicine, School of Medicine, University of Crete

Giorgos Giannakakis, Electrical and Computer Engineer, Institute of Informatics, Foundation for Research and Technology

Nikolina-Hilda Anagnostatou, Pediatrician-Neonatologist, PhD Candidate, University of Crete School of Medicine, University General Hospital of Heraklion

After the departure for professional reasons of **Psychologist** M.F. she will be replaced by a Psychologist (PhD) who will join the team following a relevant recommendation by the Scientific Responsible to the Special Account for Research Funds of the University of Crete.

Technical assistant, unnamed, will join the team following a call for expressions of interest (through the Special Account of Research Funds of the University of Crete).

AGENTS INVOLVED IN THE PROPOSED RESEARCH PROJECT

The proposed project will be implemented through the cooperation of **the Department of Psychology of the University of Crete, the University General Hospital of Heraklion and the Technology and Research Foundation.**

More specifically, Scientific Responsible of the proposed research project is **Theano Kokkinaki**, Professor of Developmental Psychology at the Department of Psychology at the University of Crete. The members of the research team who come from the University General Hospital of Heraklion, University of Crete, and in particular from the Neonatology Department are **Eleftheria Hatzidaki**, Assistant Professor of Neonatology at the School of Medicine (UoC), Director of the Neonatology Department and Neonatal Intensive Care Unit and **Nicole Anagnostatou**, Pediatrician-Neonatologist, Doctoral Candidate, Director (National Health System). **Giorgos Giannakakis**, Electrical and Computer Engineer, comes from the Foundation of Research and Technology, Institute of Informatics. In addition, **Aristides Tsatsakis**, Professor of Toxicology, **Emmanuel Tzatzarakis**, Associate Professor of Toxicology, **Eleni Vakonaki**, Biologist, Researcher, Toxicology Laboratory as well as **Theano Roumeliotaki**, Statistician, Clinic of Preventive Medicine and Nutrition, Department of Social Medicine, all come from the School of Medicine of the University Crete.

INTRODUCTION

Studying psychosocial and physiological factors related to prematurity invites the Psychology - Medicine interdisciplinary collaboration because: a) premature infants possess behavioral characteristics and have neurological immaturity that may contribute in making them difficult interactive partners (Singer, 2003) and increase their health risks (Javorka, 2017); and b) a steady increase in the neonatal survival rates since the advent of modern intensive care for the preterm infant has been related to a growing concern for preterm infants' developmental outcome and quality of life (Forcada-Guex, 2006).

Women from low socioeconomic status (SES) and in low-income countries are disproportionally affected by preterm delivery, perinatal depression / anxiety, and lack access to mental health care. The combined effects of the above are related to economic and social hardship which affects individuals, families and society as a whole, thus expanding the cycle of poverty and health care inequities (Prom, 2022). Biological, psychological, and sociological risks interact in a way that threatens the ability of the low-income family to meet the physical, social, and emotional needs of its members

(Maurer & Smith, 2013). A low socio-economic level is one of the most determinant factors associated with restricted neurodevelopment in premature infants. The higher rates of biological risks of low socio-economic status preterm infants put them at double risk for poor neurodevelopmental outcomes (Panceri, 2020; Wong & Edwards, 2013). Although developmental delays in preterm and low socio-economic level infants are well documented, there is extremely limited evidence on the early postnatal development of preterm infants exposed to both biological and environmental risk factors (Gonzalez-Gomez, 2019). It is possible that the negative effects of poverty begin during early stages of development, possibly prenatally (Blount, 2021; Hurt, 2017; Hosokawa & Katsura, 2018; Maurer & Smith, 2013).

The main aim of the proposed study is to investigate the association of certain psychosocial (postnatal depression, family functioning, social support, maternal perception of infant's intersubjectivity and attachment) and biological factors/factors of physiology (melatonin/heart rate variability) across the first year of life of preterm infants' development, with focus on low socio-economic status families. Due to limited relevant studies and contradictory findings, it was difficult to address hypotheses. Thus, the study aims to the following two objectives:

Objective 1: To explore the way psychosocial factors, such as maternal mental health, maternal perception of infant's intersubjectivity and attachment, across the first year of preterm birth are related with infants' emotional and cognitive development at 9 months (corrected age). Further, we will explore whether this association varies between infants from low and high SES. In addition, we will investigate whether other psychosocial characteristics (family functioning, perceived social support and dyadic coping) may be related to these associations and if these characteristics can moderate for the risks posed by low socio-economic status,

Objective 2: To assess the way physiological factors, such as autonomic nervous system maturation, measured according to heart rate variability, is associated with premature infants' emotional and cognitive development at 9 months (corrected age) and whether this association varies between low and high SES. Further, we will explore whether maternal derived-melatonin through breastfeeding intervenes this association.

THEORETICAL BACKGROUND OF THE PROPOSED RESEARCH PROJECT

The foetal programming hypothesis and the Developmental Origins of Health and Disease paradigm (DOHaD) postulates that the environmental influences during critical periods of development may alter the trajectory of development, with significant consequences for an individual's short and long-term health, and may explain neuropsychiatric diseases. The period from conception to early childhood is critical to the immediate and future health of the infant. The duration of gestation constitutes one of the critical perinatal factors influencing predisposition to disease. In the same theoretical framework it is proposed that it is possible to explain the connection between early life events with later adult pathology. In addition, it is possible that poverty begins to impact outcome at the earliest stages of development. Premature infants of low SES have greater long-term developmental morbidity than do premature infants of higher SES and they are at increased risk of behavioral and emotional problems (Barker, 1998;

Eriksson, 2016; Gluckman and Hanson 2006; Johnson & Marlow, 2011; Mandy & Nyirenda, 2018; Nobile, 2022).

The development of premature infants of low SES

Biological, psychological and sociological risks interact in a way that threatens the family's ability of low SES to respond to the physical, social, and emotional needs of its members (Maurer & Smith, 2013). Low SES women in the perinatal period experience greater risks for premature birth, depression, anxiety and lack of access to mental health care compared to women from high SES. Low SES constitutes one of the most evident contributors to poor neurodevelopment of preterm infants. Higher prevalence of biological risks of preterm children in socio-economically disadvantaged environments puts them at 'double jeopardy' for poor developmental outcome (Panceri, 2020; Wong & Edwards, 2013). Although developmental delays of premature infants and those from low SES are well-recognized, there is limited evidence on the very early postpartum development of infants exposed to both biological and environmental risk factors (Gonzalez-Gomez, 2019). It is likely that poverty begins to impact outcome at the earliest stages of development, even prenatally (Blount, 2021; Hurt, 2017; Hosokawa & Katsura, 2018; Maurer & Smith, 2013). In the course of the first 24 months, socioeconomic status is responsible for the variance in the cognitive development of preterm infants: the higher the SES, the higher the cognitive development of preterm infants (Panceri, 2020). In comparison to preterm infants coming from middle or high socio-economic status, preterm children from low SES are at increased risk of behavioral and emotional problems at age 4 years (Potijk, 2014).

The development of preterm infants from low SES families and relevant psychosocial factors

Maternal mental health

Low-income women are at increased risk for the development of postpartum depression symptoms in the perinatal period and they are less likely to receive appropriate treatment for depression during the postpartum period which possibly contributes to a prolonged experience of symptoms (Goyal, 2010; Kozhimannil, 2011). Meanwhile, the presence of both anxiety and depression during pregnancy has been associated with preterm birth and it interacts with aspects of low SES while stress of parents of preterm infants is high and families of low SES may face additional challenges (Enlow, 2017). Postpartum maternal mental disorders are related to long-term negative physical and psychological health outcomes for both the mother and the infant (van Haeken, 2020).

Family functioning

Low SES is associated with more stressful family life situations (Reiss, 2019). Lower SES is associated with higher rates of marital problems and it may exacerbate emotional distress for both partners in the relationship with adverse effects for adult psychological well-being (Conger, 2010). Low-income families of preterm infants are disproportionately burdened by stress and mothers experience significant anxiety in the perinatal period (Enlow, 2017). Family sense of coherence, that is the emotional bond that family members have towards one another constitutes one of the adaptation strategies in the course of transition to parenthood (Ngai and Ngu, 2016; Olson, 2019). Couples with a strong family sense of coherence probably share a common goal in bringing up a child and are motivated to mobilize all available resources to deal with the parental demands (Ngai and Ngu, 2016). Concurrent and longitudinal data show

that children from families of high levels of cohesiveness are less likely to have emotional and behavioral problems (Coe, 2018; Shigeto, 2013; Tissot, 2022).

Social support

In case of dyadic stress, such as that caused sometimes by the birth of a child (Bodenmann, 1995, 2005), new parents seek out for social support resources. During the transition to parenthood, dyadic coping reduces partner's distress, it improves his/her psychological well-being and enhances couple functioning (Bodenmann, 1995; Falconier, 2015). Social support to parents of preterm infants may function as a protective factor for the family unit (Leahy-Warren, 2020; Lutkiewicz, 2020). Lack of social relations constitutes one of the stressors faced by low SES families compared to high SES families (Leahy-Warren, 2020; Weyers, 2008). The risk of postpartum mental disorders is higher among low SES women who had insufficient social support (Fisher, 2012). Social support in the postpartum period has a direct positive correlation with family function and an indirect negative correlation with depression (Huang, 2021).Social support networks significantly influence a child's socioecological context and infant development may be associated with maternal social support. However, this relationship has not been well-examined under poverty conditions especially in the first 9 months of life (Singletary, 2021).

Intersubjectivity and parent-to-infant attachment

Human intersubjectivity is 'a process that makes it possible for subjects to detect and change each other's mind and behaviour, by purposeful, narrative expressions of emotion, intention and interest' (Trevarthen, 2001, p.18). Maternal perception of the infant's participation in the mother–infant relationship is a relevant contribute to the development of this important experience. Due to preterm birth, early mother–infant intersubjective interactions may be compromised (Carrulo, 2022). The absence of intersubjective communication in spontaneous mother–infant interaction interferes with the development of socio-emotional competences associated to neuro-developmental disorders (Trevarthen & Aitken, 2001). Preterm infants are a high-risk group regarding the formation of attachment (Korja, 2012; Leahy-Warren, 2020). Respiratory problems of preterm infants constitute a risk factor that may affect preterm infants' attachment patterns (Korja, 2012; Leahy-Warren, 2020). Family cohesion is closely related to attachment (Youngblut et al 1993). Within the first year of life, higher level of maternal depressive symptoms is associated with preterm birth, poor attachment and poor social support. Surprisingly, there is evidence of high mother-to-infant attachment for mothers with preterm infants in the course of the first year of life (Leahy-Warren, 2020). However, some mothers of preterm infants describe mixed emotional experiences and an inner struggle with their bonding process to their children (Widding & Farooqi, 2016). The combination of premature birth and low SES may affect the quality of attachment and may hinder parental bonding with poor possible developmental difficulties (Hoffenkamp, 2012; Wille, 1991).

Biological factors and the development of preterm infants from low SES families

Melatonin

Besides regulating the circadian rhythm, melatonin has a wide range of biological functions, including cardiovascular health effects, especially in infants (Katzer,2016; Qin,2019; Gombert & Codoñer-Franch, 2021). Melatonin in human milk is important for normal neurodevelopment, it plays an important role in newborn synchronization with the mother's rhythm, it entrains rhythms in the cardiovascular system that are essential to neonatal homeostasis and function, and may contribute to better growth and development with long-term outcomes (Gombert & Codoñer-Franch, 2021). Melatonin is detected at 24 weeks gestation and

it reaches at a peak in the third trimester. During the first 3 months infants experience a transient deficiency in melatonin, due to suboptimal melatonin production and immature circadian rhythmicity (D'Angelo,2020). Breastmilk is the only source of melatonin for the infant, and especially the preterm neonate, during the first few months of life (Gombert & Codoñer-Franch, 2021). This property has special importance in preterm newborns, since they lack the last part of the pregnancy that will provide the highest melatonin levels, are under great risk for oxidative stress and have a delay in the rhythmic expression of melatonin with respect to full-term infants. However, preterm breast milk has a higher concentration of melatonin than term breast milk. It is unclear to what extent socioeconomic status influences breast milk composition and circadian rhythm variation (Italianer, 2020; Samuel, 2020). Lower socioeconomic status may exacerbate the risk of poor nutritional and health status in pregnant women and nutritional deficiencies can impact circadian rhythmicity in human milk composition (Freisling, 2006; Italianer, 2020). In addition, maternal postpartum stress and negative mood have been associated with higher melatonin in milk samples (Groër, 2005). The role of melatonin for preterm infant development is unclear. To our knowledge, only one study showed that improved autonomic function at 2 weeks of age was associated with higher Mental Developmental Index scores at 9 months when related to the amount of melatonin at 4, 6, and 9 months of age (Goldstein Ferber, 2011). It is important to study the way maternally-derived melatonin is involved in early development because melatonin disturbances have been reported in a number of psychiatric and developmental disorders, such as depression, schizophrenia and autism spectrum disorder and so on (Tordjman, 2017).

Autonomic nervous system maturation and heart rate variability

In infants born at term, the normal increase in parasympathetic tone is evidenced in the increased high-frequency heart rate variability (HRV) (Mulkey and du Plessis, 2019). Gestational age in preterm infants significantly correlates with heart rate (HR) and HRV parameters, the lower is gestational age, the higher is mean HR for longer time and lower HRV. A decrease in HRV in linked to vulnerability to stress whereas an increase represents physical and mental adaptability (Javorka, 2017; Suga, 2019). Research shows an association between maturation of the autonomic nervous system maturation of preterm infants with short- and long-term implications for social and emotional development, the manifestation of externalizing, internalizing and cognitive/academic problems and neuropsychiatric disorders in children (Doussard-Roosevelt, 1997; Doussard-Roosevelt, McClenny & Porges, 2001; Field & Diego, 2008; Graziano & Derefinko, 2013; Mulkey & de Plessis, 2019; Porges & Furman, 2011). In addition, premature newborns of depressive mothers had lower vagal tone than those of nonsymptomatic mothers (Jones, 1998; Field, 1995). Only a limited number of studies has provided contradictory findings regarding the association between family functioning and measurements of maturation of autonomic nervous system early in life (Porter, 2003; Moore, 2010; Graham, 2010).

New knowledge to be acquired by this study

The proposed study is novel in the following ways: **(1)** The role of melatonin for preterm infant development is unclear (Tauman, 2002) and under-investigated. Towards this direction, we aim to investigate the role of melatonin on infant development through a suspected multifactorial early developmental pathway combining psychosocial and biological factors; **(2)** The proposed study on determinants of premature infant development is longitudinally. Appropriate longitudinal studies for premature infants are scarce. In order to fully recognize the difficulties of children born

preterm, it is important to follow them individually, through several developmental stages (Jansen, 2021). Longitudinal studies should be pursued in forthcoming years to deepen our understanding of the pathways leading to heightened risk for adverse developmental outcomes in preterm infants (Provenzi, 2017); **(3)**This is a multi-method study including physiological and hormone measures, one observational instrument and self-report validated questionnaires. Using multiple outcome measures in infant research is one way to increase rigor, and at the same time, enable us to more accurately interpret our data (LoBue, 2020); **(4)** Evidence on premature infant development is limited in Greece (Koutra, 2012). To the best of our knowledge the proposed study will be the first of this kind in Crete, Greece at a time after the financial crisis and in the course of the pandemic. This is important because the financial crisis of the last decade has increased the number of people living in extreme poverty in Greece and led to partial operation of mental health services (Papadakaki, 2021; Tsobanoglou, 2014; Giannopoulou & Tsobanoglou, 2020). What is more, the pandemic increased income inequalities (Vavoura & Vavouras, 2022) and affected adversely the mental health of Greeks (Parlapani, 2020; Vatavali, 2020). In Crete, poverty and mental health problems, especially for women, are prevalent and interconnected issues. Multiproblem families have been said to be on the rise (Papadakaki, 2021). Further, preterm births constitute a major public health issue in Greece (Vlachadis, 2013). Preterm birth rate has increased fourfold in the past two decades. These findings pose dramatic challenges for public health and emphasize the need for preventive interventions to be implemented (Vlachadis, 2013).

Scientific and social impact of the proposed research project

In Medicine, autonomic nervous system ANS activity constitutes an index of health and has a critical role for maintaining cardiovascular and respiratory homeostasis, but, at the same time, it is also intrinsically connected to higher brain systems involved in the emotional and psychological aspects of human life within Psychology (Mulkey & du Plessis, 2019; Porges & Furman, 2011). In addition, from the Psychology perspective, melatonin is important for normal neurodevelopment (Braam, 2018; Qin, 2019) and also, for Medicine, it appears a promising molecule to prevent brain insults associated with prematurity (Biran, 2019).

Perinatal mental health problems are a major public health concern globally (Tripathy, 2020). Maternal perinatal mental health disorders have persistent effects on behavioural, physiological and immunological functioning throughout the lifespan and may even be evident across generations (Coussons-Read, 2013). In addition, prematurity is considered a chronic and multigenerational condition (Pravia & Benny, 2020). Prevention goals requires effective interventions that reach women at risk for, but prior to, the development of a depressive disorder. Early interventions improve sub-clinical symptomatology for at risk dyads at a crucial time of early postpartum period (Scorza, 2020). In connection to this, potential delays in infants born preterm may be amenable to early intervention. Early detection of developmental delays is a process of ongoing assessment and consideration of key perinatal events known to affect long-term outcomes (Kwong et al 2022). The findings of this study may highlight the need for future community-based prevention efforts and may convince policy makers and government to increase evidence-based interventions and family-focused care targeted on the promotion of perinatal mental health-care of mothers and preterm infants' development from low SES. Designing evidence-based e-Health screening applications with the aim to improve universal access to developmental screening by

bringing services into the homes of vulnerable populations (Van der Merwe, 2019) may take the form of targeted interventions for significant public health benefit. In addition, the results of the proposed research project may highlight the necessity for further discussions for the priorities of policymakers and the responsibility of society to counteract health inequalities (M'hamdi, 2017) and to interrupt the pattern of intergenerational mental illness and suffering (Prom, 2022). This is important because the perinatal mental health literature has focused on individual women as the main agent for change while social determinants of mental health, such as poverty, are of critical importance for women in the perinatal period (Howard & Khalifeh, 2020). At the economic level, identification of early risk factors for the development of preterm infants may has implications for policymakers since preterm infants incur higher early intervention costs (Clements, 2007).

METHODOLOGY

Conceptual Framework of the Study

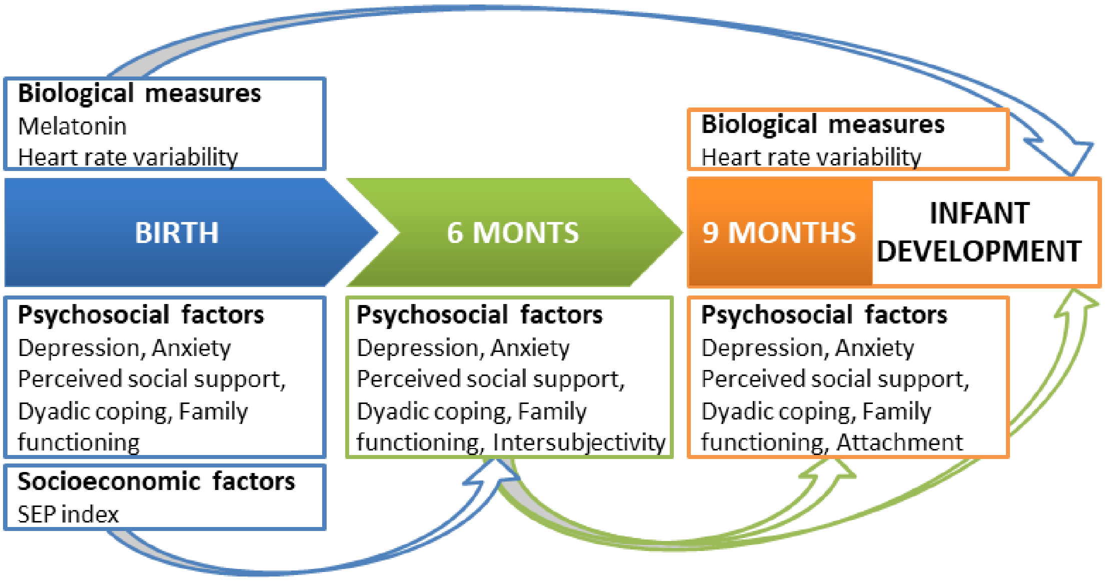

Diagram: Conceptual framework of the proposed research project and timeline of assessments of psychosocial and biological factors

Participants

Mothers and their preterm neonates (<37 weeks) (from birth till 9 months corrected age) who will be hospitalized in the Neonatology Department/Neonatal Intensive Care Unit of the University General Hospital of Heraklion will participate in the study. Participants of this study will be approached through the Gynecology-Obstetrics Clinic and the Department of Neonatology, Neonatal Intensive Care Unit (NICU) of the University General Hospital of Heraklion.

Inclusion criteria

Inclusion criteria for mothers include the following: a) parents are not divorced; b) both parents are older than 20 years of age; and c) mother intends to breastfeed at least the first 28 days of neonate's life. Inclusion criteria for neonates include prematurity (gestation age < 37weeks) and the absence of serious neurological/brain abnormalities.

Exclusion criteria
Exclusion criteria include the following: a) mothers suffer from a psychiatric illness; b) they have issues with drug or substance abuse; c) they are not biological parent; and d) they belong to a same-sex couple. The restriction for the participation of homosexual couples is justified by the lack of relevant evidence on early development of premature infants/children in Greece. This lack would result in a weakness to compare and utilize the results of this study; e) mothers who do not intend to breastfeed; f) surrogate mothers g) mothers from geographic division outside Crete which would make it difficult for them to participate in the follow up (at 9 months corrected age).

Data Collection Procedure
The members of the research team who will be responsible for approaching participants and for data collection (N.A. and no-named member) will communicate with the mothers and will inform them for the study (aim of the study, procedure and duration of participation, assurance of anonymity and confidentiality, voluntary nature of participation as well as wider usefulness of the research). In this way, every mother will be given the opportunity to easily decide on the participation of herself and her newborn in the research, having full information about what is included in it. Before signing the relevant consent forms, the researchers will offer additional clarifications and answer any questions, including in any case information on the benefits of participating in the research. In addition, mothers will be informed by letter about the possibility of submitting complaints to the Ethics Committee of the University of Crete, the Research Ethics and Ethics Committee of the University General Hospital of Heraklion and to the Personal Data Processing Officer of the University of Crete (see below). After the detailed presentation of the research process and the provision of answers to any questions, written informed consent will be obtained for the mothers' voluntary participation in accordance with the terms of Declaration of Helsinki (World Medical Association Declaration of Helsinki, Ethical Principles for Medical Research Involving Human Subjects).

Measures

A.Socio-economic factors

In the frame of the proposed research project, we aim to assess various early-life stressors during pregnancy and early childhood. We will identify socio-demographic stressors with major consequences for health and development. These data will be related to socio-economic status, including parental education, occupation, type of employment contract, family income, family size, child care, house property and size of it. These data will be combined in order to conduct a probabilistic linkage with the *European Union Statistics on Income and Living Conditions (EUSILC)* to obtain information for socio-economic status and social disadvantage in early life. The combination of relevant information will be used to derive a socio-economic position (SEP) index (Pizzi, 2020).

B.Psychosocial factors

Maternal Depression
The *Edinburgh Postnatal Depression Scale* (EPDS, Cox, 1987) will be used to screen possible depressive symptoms in new mothers. The EPDS is a 10-item self-rating scale designed to particularly target populations during both the antenatal and post-natal

period. Each item is scored from 0 to 3. The EPDS has been validated in a variety of settings and community samples with the majority of studies focusing on the 6-8 week postpartum period (Chaudron, 2010). The EPDS has been validated in Greek (Vivilaki, 2009).
The *Beck Depression Inventory-II* (BDI-II; Beck, 1996) will be used to screen maternal symptoms of depression experienced in the past week before administration. The BDI-II is a 21-item self-report test with 4 response options per item. The BDI-II has been validated in Greek (Giannakou, 2013).

Maternal anxiety
The *Spielberger State-Trait Anxiety Inventory for Adults* (STAI, Spielberger, 1983) will be used to measure maternal anxiety. The STAI is a 40-item self-report measure with a 4-point Likert-type scale for each item. The STAI has two scales: State anxiety (which assesses how one feels at the moment of administration, 20 items) and Trait anxiety (which assesses how one generally feels, 20 items). The STAI has been validated in Greek (Liakos, 1984).

Family Functioning
The *Family Adaptability and Cohesion Evaluation Scales IV Package* (FACES IV; Olson, 1979; Olson, 2019) will be used to assess perceived family functioning. The FACES IV Package contains the six scales from FACES IV (42 items), the Family Communication Scale (FCS) and the Family Satisfaction Scale (FSS) (62 items in total). The FACES has been validated in Greek (Koutra, 2013).

Perceived Social Support
The *Multidimensional Scale of Perceived Social Support* (MSPSS, Zimet, 1988) will be used to assess the perception of social support mothers receive from three sources, (each corresponds to a subscale): family, friends, and significant other. The MSPP is a 12-item reliable and valid self-rating scale (each subscale consists of four items). The MSPSS has been validated in Greek (Theofilou, 2015).

Dyadic Coping
The *Dyadic Coping Inventory* (DCI, Bodenmann, 2008; Ledermann, 2010) will be used to measure dyadic coping behaviors. The DCI is a 37-item reliable and valid instrument with 10 subscales. The DCI has been validated in Greek (Roussi & Karademas, 2016).

Intersubjectivity
The *Maternal Perception of Infant's Intersubjectivity Questionnaire* (MPIIQ, Carrulo, 2022) will be used to assess maternal perception of the infant's intersubjectivity. The MPIIQ is a 22-item self-report questionnaire with good psychometric properties. Items are grouped in three factors: Factor 1 is related to maternal perception about the infant's competence at the interaction with the mother, Factor 2 refers to maternal perception of infants' behaviors that express emotional states and Factor 3 is related to maternal perceptions about the infants' competence to express their own initiatives.

Attachment
The *Maternal Postnatal Attachment Scale* (MPAS, Condon & Corkingdale, 1998) will be used to assess mother's subjective feelings of attachment to her infant. The MPAS is a 19-item self-report questionnaire. The MPAS includes three subscales: quality of

attachment (9 items), absence of hostility toward the infant (5 items) and pleasure in the interaction (5 items).

C.Biological measurements

Melatonin

Breastmilk melatonin concentrations

Mothers of preterm neonates will be asked to collect 5-10ml of breastmilk with the use of an electrical pump between 10:00-14:00 at three specific time points: 3rd-5th day (colostrum), 10th-14th day (transitional milk) and 20th-28th day (mature milk). The milk will be collected in a sterile container, transported at 4°C, and frozen immediately at -20^{0}C until analysis. The melatonin levels in breast milk samples will be determined by a commercially available ELISA assay.

Melatonin concentration in neonatal blood sample and in umbilical cord blood sample

An umbilical cord blood sample of 1-2ml will be collected at every preterm delivery or caesarean section, in order to measure the melatonin level at the time of birth. Blood samples from the preterm neonate will be collected as follows:

a. for premature neonates > 33 weeks, 2 samples will be collected (4th-7th day of life and 10th-14th day, which will coincide with a gestational age of 35-36 weeks); b.for premature neonates <33 weeks, 3 samples will be collected (4th-7th day of life, 10th-14th day and at a date that will coincide with a gestational age of 35-36 weeks).

The blood will be collected from the clinic's medical staff, along other scheduled blood tests, so that no extra interventions are performed on the neonates for the purpose of this study. The time of blood sampling will be between 8:00-10:00 am and the amount needed is estimated at 500μL.

Umbilical cord blood and neonate blood samples will be centrifuged at 3000 g for 5 min, and then the plasma will be separated. Samples will be frozen at −20°C until analysis. The levels of melatonin will be determined by an ELISA assay according to the manufacturer's instructions.

Heart rate variability

Neonates' / infants' and maternal ECG measurements will be obtained at 2 successive time intervals [for neonates/infants at the neonatal period and at 9 months after the birth (corrected age) and for the mothers at birth] and HRV measurements will be carried out through short-term variability which provides important information about the maturation of the ANS in newborns (Lavanga, 2021; Longin, 2006). The following HRV parameters will be assessed: the time-domain (SDNN, HRm, HRstd, RMSSD, NN50, pNN50, HRV triangular index), the frequency-domain (Total power, LF, HF, LF/HF, LFnorm, HFnorm) as well as non-linear indices (ApEn, DFA α1, α2) (Giannakakis, 2019) in order to identify the cardiac activity and SNS/PNS activation patterns. HRV measurements will be carried out through SEER 1000, ECG Recorder, General Electric (Version 1.0, 2067634-077 Revision F).

Information for dietary habits and lifestyle

Finally, information for dietary habits and other health-related and lifestyle information that may affect maternal levels of melatonin concentrations will be assessed (Peuhkuri, 2012).

Assessment of premature infants' development

At 9 months (corrected age), the social and cognitive development of infants will be assessed by the administration of the *Bayley Scales of Infant and Toddler Development, 3rd Edition* (Bayley, 2006) which is a standardized, diagnostic developmental assessment instrument for infants and young children between one and 42 months. Bayley-III is comprised of 5 scales: cognitive scale, language scale, motor development scale, social-emotional scale and adaptive behavior scale.

Issues of ethics and research ethics

Research participants take part in the study under the principles of informed consent and complete confidentiality. Following the relevant information of the new mothers about the research and the research process (by the members of the research team who will be responsible for the collection of the data), the mothers will have the possibility to easily decide to participate themselves and their newborn in the research having full information about what is included in it. Participating mothers and their newborns/infants will not suffer any deception, harm or loss and all reasonable precautions will be taken to preserve their well-being and dignity. research findings (Willig, 2001).

In addition, the proposed research project fully complies with the indications of National Legislation and National Law on the issues related to ensuring the protection of the privacy of personal data.

In the proposed longitudinal study, mothers and their newborns will participate. More specifically, the members of the research team who will be responsible for approaching the participants and collecting the data will inform the new mothers in detail about the research through the Information Form (attached) about the aim of the research, the procedure and duration of participation, the assurance of anonymity and confidentiality, the voluntary nature of participation as well as the broader usefulness of the research. In this way, each mother will be given the opportunity to effortlessly choose her possible participation in the research, having full information about what is included in it.Before signing the relevant consent forms, the researchers will offer additional clarifications and answer any questions, including in any case information on the benefits of participating in the research. In addition, the mothers will be informed in a relevant letter about the possibility of submitting complaints to the Ethics Committee of the University of Crete and to the Personal Data Processing Officer of the University of Crete.

After the detailed presentation of the research process and the provision of answers to any questions, written informed consent will be obtained for the voluntary participation of the mothers in accordance with the terms of the Declaration of Helsinki.

Next, the mothers who have agreed to take part in the research will be asked:

a) to consent to the measurement of melatonin in blood taken from the umbilical cord (opt-out approach). In case of non-consent, the corresponding sample will be destroyed,

b) to consent to the measurement of melatonin in the blood of the newborn during his hospitalization (the blood will come from routine blood draws),

c) to provide 3 samples of breast milk at specific time periods (see above) for the purpose of measuring melatonin levels,

d) to complete a battery of questionnaires to assess various psychosocial factors at the three defined time periods (at birth, 6 and 9 months);

e) to consent to the evaluation of the heart rate variability of the newborns at the 2 defined time periods and of the mothers [during the first week of life and at 9 months (corrected age)];

f) to consent to neurodevelopmental assessment of their infants through administration of the Bayley Scales of Infant and Toddler Development, 3rd Edition (Bayley, 2006) at 9 months (corrected age).

The questionnaires will be completed either in person or online through a special platform. The link leading to the special platform will be sent to the email address of the mothers which will have been declared by them during the written informed consent. The information and consent forms and the participant/code list will be kept in a locked cabinet in the office of the Scientific Responsible at the Department of Psychology of the University of Crete for a period of 7 years and will then be destroyed with a shredder. Once digital data is entered into the database, it will be permanently deleted.

Study data will be stored in special computer logs anonymously. More specifically, to maximize the anonymization of the physical and digital material that will come during the data collection phase, each participant will initially be given a code that will contain letters and/or numbers. This code will come from the random mixing of letters/numbers through an electronic lottery process. Also, during the same phase, each code will be completed by an attribute that will indicate the matching of each participant with the family from which he/she comes. As with the codes for each participant, the matching features will contain letters and/or numbers and will be generated by randomly mixing them through an electronic lottery process. Participant code assignment will occur once during the study. None of the researchers managing the data will have access to the names of the participants and only the Scientific Responsible (PI) will be able to re-associate the saved names with their passwords if necessary. The key that will match the code with the parent's name will only be known to the Scientific Responsible of the study and will be kept in a place in the PI’s office at the Department of Psychology of the University of Crete (Rethymno) (the Rethymno Campus is guarded by special staff).

Furthermore, all information and data collected during the proposed project will be kept strictly confidential. In particular, all types of electronic files (such as databases, spreadsheets, etc.) containing identifiable information will be specially coded in such a way that it is not possible to reveal the identity of the participants.These codes will be kept in a separate cabinet from the one where the above matching keys are kept in the EY office at the Department of Psychology of the University of Crete. Only the members of the research team of the program will have access to the physical and digital

archive data. Any computer hosting the above files will also be password protected and not accessible via the internet.

The members of the research team who will contribute to the approach of the participants and the collection of the data will inform the participants from the beginning about the safety and their voluntary participation in the research in accordance with the terms of the Declaration of Helsinki. In the event that issues regarding the safety of the participants arise, the Scientific Responsible of the proposed project will contact and discuss with the participants their desire to continue, or discontinue their participation in the study. In the event that the participants decide to discontinue their participation and withdraw from the sample, the Scientific Responsible of the project in collaboration with the members of the research team will destroy the personal data of the specific participants. In the event of manifestation of symptomatology in the mental health of a participant mother that needs support from a mental health specialist, there is a provision to stop participation in the research project and the mother to be referred to a relevant structure/public service of the prefecture in which resides, or in the health structure closest to her residence.

Under all the above conditions, we consider that the proposed research program is not governed by any moral or ethical concerns.

Procedure for submitting complaints

For any complaints regarding the conduct of the research, the participants can appeal to the Ethics Committee of the University of Crete, ehde@uoc.gr, and to the Ethics Committee of the University General Hospital of Crete, researchprot@pagni.gr

For any complaint regarding the management of the personal data of the participants, participants can contact the Personal Data Processing Officer of the University of Crete, Ms Elpida Vamvakas, e-mail: dpo@uoc.gr and in any case the Personal Data Protection Authority, e-mail: complaints@ dpa.gr

All members of the research team will be trained in data management, data protection, anonymity and confidentiality of personal files.

Justification for the Sample Size Calculation and Data Analyses Plan

Power calculations will be examined assuming a level of statistical significance of 5% and an 80% power. The results of the study will be based on a sample size of at least N = 100 mother-infant pairs in order to detect a difference of at least 10 points.

Initial exploratory analysis will be used to understand variable distributions and to identify transformations that satisfy modeling assumptions, to understand correlations, and to identify extreme observations. For the investigation of the objectives of the study, Generalized Additive Models (GAMs) and Structural Equation Modeling – SEM will be employed while the association between psychosocial and biological factors with developmental outcomes of preterm infants at 9 months (corrected age), comparing families of low, middle/high socio-economic status, will be carried out through the application of linear regression modeling and generalized linear modeling.

References

Adhikari, K., Patten, S.B., Williamson, T., *et al.* (2020). Neighbourhood socioeconomic status modifies the association between anxiety and depression during pregnancy and preterm birth: a Community-based Canadian cohort study. *BMJ Open*, 10(2).

Ask, T.F., Ranjitkar, S., Ulak, M., *et al.* (2019). The association between heart rate variability and neurocognitive and socio-emotional development in Nepalese infants. *Frontiers in Neuroscience*, 13:411.

Bayley, N. (2006). *Bayley Scales of Infant and Toddler Development,* Third Edition: Pearson.

Barker, D. J. (1998). In utero programming of chronic disease. *Clinical Science*, 95(2), 115-128.

Barker, D. J. (2002). Fetal programming of coronary heart disease. *Trends in Endocrinology Metabolism*, 13, 364-368.

Barker, D. J. (2007). The origins of the developmental theory. *Journal of Internal Medicine*, 261, 412-417.

Blount, A.J., Adams, C.R., Anderson-Berry, A.L., *et al.* (2021). Biopsychosocial Factors during the Perinatal Period: Risks, Preventative Factors, and Implications for Healthcare Professionals. *International Journal of Environmental Research and Public Health*, 18(15):8206.

Bodenmann, G. (1995). A systemic-transactional conceptualization of stress and coping in couples. *Swiss Journal of Psychology / Schweizerische Zeitschrift für Psychologie / Revue Suisse de Psychologie, 54*(1), 34–49.

Bodenmann, G. (2005). Dyadic coping and its significant for marital functioning. In T. Revenson, K. Kayser, & G. Bodenmann (Eds.), *Couples coping with stress: Emerging perspectives on dyadic coping* (pp. 33-50). APA.

Braam, W., Ehrhart, F., Maas, A.P.H.M., *et al.* (2018). Low maternal melatonin level increases autism spectrum disorder risk in children. *Research in Developmental Disabilities,* 82:79-89.

Brown, L. (2007). Heart rate variability in premature infants during feeding. *Biological Research for Nursing*, 8(4).

Biran, V., Decobert, F., Bednarek, N., *et al.*(2019). Melatonin levels in preterm and term infants and their mothers. *International Journal of Molecular Sciences*, 20(9):2077 Carrulo, J., Justo, J.M.R.M., Figueiredo, B. (2022). Maternal perception of infant's intersubjectivity: a questionnaire. *Journal of Reproductive and Infant Psychology*, 1-11.

Cena, L., Mirabella, F., Palumbo, G., *et al.* (2021). Prevalence of maternal antenatal and postnatal depression and their association with sociodemographic and socioeconomic factors: A multicentre study in Italy. *Journal of Affective Disorders*, 279, 217-221.

Chaudron, L.H., Szilagyi, P.G., Tang, W., *et al.* (2010). Accuracy of depression screening tools for identifying postpartum depression among urban mothers. *Pediatrics*, 125(3): e609-17.

Clements, K.M., Barfield, W.D., Ayadi, M.F., *et al.* (2007). Preterm birth-associated cost of early intervention services: an analysis by gestational age. *Pediatrics*, 119(4):e866-74.

Coe, J.L., Davies, P.T., Sturge-Apple, M.L. (2018). Family cohesion and enmeshment moderate associations between maternal relationship instability and children's externalizing problems. *Journal of Family Psychology*, 32(3):289-298.

Cohen Engler A, Hadash A, Shehadeh N, *et al.* (2012). Breastfeeding may improve nocturnal sleep and reduce infantile colic: potential role of breast milk melatonin. *European Journal of Pediatrics*, 171(4), 729-32.

Condon, J. T., & Corkindale, C. J. (1996). The assessment of parent-to-infant attachment: Development of a self-report questionnaire instrument *Journal of Reproductive and Infant Psychology,* 16:1, 57-76.

Conger, R.D., Conger, K.J., Martin, M.J. (2010). Socioeconomic status, family processes, and individual development. *Journal of Marriage and Family*, 72(3):685-704.

Coussons-Read, M.E. (2013). Effects of prenatal stress on pregnancy and human development: mechanisms and pathways. Obstetric Medicine , 6(2):52-57. Cox, J. L., Holden, J. M., Sagovsky, R. (1987). Detection of postnatal depression: development of the 10-item Edinburgh Postnatal Depression Scale. *The British Journal of Psychiatry*, *150*(6), 782-786.

D'Angelo, G., Chimenz., R, Reiter., R.J., *et al.* (2020). Use of Melatonin in Oxidative Stress Related Neonatal Diseases. *Antioxidants*, 9(6):477.

Dierckx, B., et al. (2009). Maternal psychopathology influences infant heart rate variability: Generation R study. *Psychosomatic Medicine, 71*, 313-321.

Doussard-Roosevelt, J. A., McClenny, B. D., Porges, S. W. (2001). Neonatal cardiac vagal tone and school-age developmental outcome in very low birth weight infants. *Developmental Psychobiology: The Journal of the International Society for Developmental Psychobiology, 38*(1), 56-66.

Doussard-Roosevelt J, Porges S, Scanlon J, *et al.* (1997). Vagal regulation of heart rate in the prediction of developmental outcome for very low birth weight preterm infants. *Child Development, 68*, 173–86.

Enlow, E., Faherty, L.J, Wallace-Keeshen, S., *et al.* (2017). Perspectives of low socioeconomic status mothers of premature infants. *Pediatrics*, 139(3): e20162310. Eriksson, J. G. (2016). Developmental origins of health and disease – from a small body size at birth to epigenetics. *Annals of Medicine*, 48 (6), 456-467.

Falconier, M. K., Jackson, J. B., Hilpert, P., *et al.* (2015). Dyadic coping and relationship satisfaction: A meta-analysis. *Clinical Psychology Review*, *42*, 28–46. Field, T., Diego, M. (2008). Vagal activity, early growth and emotional development. *Infant Behavior and Development, 31*(3), 361-373.

Field, T., Pickens, J., Fox, A. N., *et al.* (1995). Vagal tone in infants of depressed mothers. *Developmental Psychopathology, 7*(2), 227-231.

Fisher, J., Cabral de Mello, M., Patel, V., *et al.* (2012). Prevalence and determinants of common perinatal mental disorders in women in low- and lower-middle-income countries: a systematic review. *Bulletin of the WHO*, 90(2).

Florian, S., Ichou, M., Panico, L. (2021). Parental migrant status and health inequalities at birth: The role of immigrant educational selectivity. *Social Science & Medicine*, 278:113915

Forcada-Guex M, Pierrehumbert B, Borghini A, *et al.* (2006). Early dyadic patterns of mother-infant interactions and outcomes of prematurity at 18 months. *Pediatrics*, 118(1):e107-14.

Giannakakis, G., Marias, K., & Tsiknakis, M. (2019). A stress recognition system using HRV parameters and machine learning techniques, 8th International Conference on Affective Computing and Intelligent Interaction Workshops and Demos (ACIIW), pp. 269-272.

Giannakou, M., Roussi, P., Kosmides, M. E., *et al.* (2013). Adaptation of the beck depression inventory-II to greek population. *Hellenic Journal of Psychology, 10*, 120-146.

Giannopoulou, I., Tsobanoglou, G.O. (2020). COVID-19 pandemic: challenges and opportunities for the Greek health care system. *Irish Journal of Psychological Medicine*, 37(3):226-230.

Gluckman, P. D. Hanson, M. A. (2006). The developmental origins of health and disease: The breadth and importance of the concept. In Wintour E. M. & Owes, J. A. (Eds), Early Life Origins of Health and Disease. Springer Media.

Ferber, S.G., Als, H., McAnulty, G., Peretz, H., *et al.* (2011). Melatonin and mental capacities in newborn infants. *Journal of Pediatrics*, 159(1):99-103.e1.

Gombert, M., Codoñer-Franch, P. (2021). Melatonin in early nutrition: Long-Term effects on cardiovascular system. *International Journal of Molecular Sciences*, 22(13):6809.

Gonzalez-Gomez, N., O'Brien, F., Harris, M. (2020). The effects of prematurity and socioeconomic depreivation on early speech perception: A story of two different delays. *Developmental Science*, 24(2): e 13020.

Goyal, D., Gay, C., Lee, K.A. (2010). How much does low socioeconomic status increase the risk of prenatal and postpartum depressive symptoms in first-time mothers? *Womens Health Issues*, 20(2): 96-104.

Graham, A., Ablow, J. C., Measelle, J. R. (2010). Interparental relationship dynamics and cardiac vagal functioning in infancy. *Infant Behavior and Development, 33*, 530-544.

Graziano, P., Derefinko, K. (2013). Cardiac vagal control and children's adaptive functioning: A meta- analysis. *Biological Psychology, 94*, 22-37.

Groër, M., Davis, M., Casey, K., *et al.* (2005). Neuroendocrine and immune relationships in postpartum fatigue. *MCN: The American Journal of Maternal/ Child Nursing,* 30(2):133-8.

Hoffenkamp, H.N., Tooten, A., Hall, R.A., *et al.* (2012). The impact of premature childbirth on parental bonding. *Evolutionary Psychology*, 17;10(3):542-61.

Howard, L.M., Khalifeh, H. (2020). Perinatal mental health: a review of progress and challenges. *World Psychiatry*, 19(3):313-327.

Huang, Y., Liu, Y., Wang, Y. *et al.* (2021) Family function fully mediates the relationship between social support and perinatal depression in rural Southwest China. *BMC Psychiatry* **21**, 151.

Hurt, H., Betancourt, L.M. (2017). Turning 1 year of age in a low socioeconomic environment: A portrait of disadvantage. *Journal of Developmental and Behavioral Pediatrics*, 38(7):493-500.

Hosokawa, R., Katsura, T. (2018). Role of parenting style in children's behavioral problems through the transition from preschool to elementary school according to gender in Japan. *International Journal of Environmental Research and Public Health,* 16(1):21.

Italianer, M.F., Naninck, E.F.G., Roelants, J.A., *et al.* (2020). Circadian variation in human milk composition, a systematic review. *Nutrients*, 12(8):2328.

Jansen, L., Peeters-Scholte, C.M.P.C.D., van den Berg-Huysmans, A. A., *et al.* (2021) Longitudinal Follow-Up of Children Born Preterm: Neurodevelopment From 2 to 10 Years of Age. *Frontiers in Pediatrics*, 9:674221.

Javorka, K., Lehotska, Z., Kozar, M., *et al.* (2017). Heart rate variability in newborns. *Physiological Research, 66* (Suppl. 2), S203-S214.

Johnson S, Marlow N. (2011). Preterm birth and childhood psychiatric disorders. *Pediatric Research*, 69(5 Pt 2):11.

Jones, N. A., Field, T., Fox, N. A., *et al.* (1998). Newborns of mothers with depressive symptoms are physiologically less developed. *Infant Behavior and Development, 21* (3), 537-541.

Katzer, D., Pauli, L., Mueller, A. *et al.* (2016). Melatonin Concentrations and Antioxidative Capacity of Human Breast Milk According to Gestational Age and the Time of Day. *Journal of Human Lactation*, 32(4):NP105-NP110.

Korja, R, Latva, R., Lehtonen, L. (2012). The effects of preterm birth on mother-infant interaction and attachment during the infant's first two years. *Acta Obstetricia et Gynecologica Scandinavica*, 91(2):164-73.

Koutra, K., Chatzi, L., Roumeliotaki T., *et al.* (2012). Socio-demographic determinants of infant neurodevelopment at 18 months of age: Mother-Child Cohort (Rhea Study) in Crete, Greece. *Infant Behavior and Development*, 35(1):48.

Kozhimannil, K.B., Trinacty, C.M., Busch, A.B., *et al.* (2011). Racial and ethnic disparities in postpartum depression care among low-income women. *Psychiatric Services,* 62(6):619-25.

Kwong, A.K., Boyd, R.N., Chatfield, M.D., *et al.* (2022). Early Motor Repertoire of Very Preterm Infants and Relationships with 2-Year Neurodevelopment. *Journal of Clinical Medicine*, 25;11(7):1833.

Koutra, K., Triliva, S., Roumeliotaki, *et al.* (2013). Cross-cultural adaptation and validation of the Greek version of the family adaptability and cohesion evaluation scales IV package (FACES IV Package). *Journal of Family Issues, 34*(12), 1647-1672. Lavanga, M., Heremans, E., Moeyersons, J., *et al.* (2021). Maturation of the Autonomic Nervous System in Premature Infants: Estimating Development Based on Heart-Rate Variability Analysis. *Frontiers in Physiology*, 11:581250.

Leahy-Warren, P., Coleman, C., Bradley, R. *et al.* (2020) The experiences of mothers with preterm infants within the first-year post discharge from NICU: social support, attachment and level of depressive symptoms. *BMC Pregnancy Childbirth,* 20, 260.

Liakos, A., Giannitsi, S. (1984). Reliability and validity of the modified Greek version of the Spielberger State-Trait Anxiety Inventory. *Encephalos*, 21, 71-76.

LoBue, V., Reider, L.B., Kim, E., *et al.* (2020). The importance of using multiple outcome measures in infant research. *Infancy*, 25(4):420-437.

Longin, E., Gerstner, T., Schaible, T., *et al.*.(2006). Maturation of the autonomic nervous system: differences in heart rate variability in premature vs. term infants. *Journal of Perinatal Medicine*, 34(4):303-8.

Lutkiewicz, K. (2020). Social Support, Perceived Stress, Socio-Demographic Factors and Relationship Quality among Polish Mothers of Prematurely Born Children. *Int Journal of Environmental Research and Public Health*, 17(11).

Malow, B.A., Adkins, K.W., Reynolds, A., *et al.* (2014). Parent-based sleep education for children with autism spectrum disorders. *Journal of Autism and Developmental Disorders,* 44(1):216-28.

Mandy, M., Nyirenda, M. (2018). Developmental Origins of Health and Disease: the relevance to developing nations. *International Health*, 10(2):66-70.

Maurer, F. A., Smith, C. M. (2013). Community/public health nursing practice: health for families and populations (5th ed.). Elsevier/ Saunders.

M'hamdi, H. I., Beaufort, I., Jack, B. W., *et al.* (2017). Responsibility in the age of Developmental Origins of Health and Disease (DOHaD) and epigenetics. *Journal of Developmental Origins of Health and Disease*, 9(1): 1-5.

Moore, G. A. (2010). Parent conflict predicts infants' vagal regulation in social interaction. *Developmental Psychopathology, 22*(1), 23-33.

Mulkey, S. B., du Plessis, A. J. (2019). Autonomic system development and its impact on neuropsychiatric outcome. *Pediatric Research, 85*(2), 120-126.

Ngai, F.W., Ngu, S.F. (2014). Family sense of coherence and family adaptation among childbearing couples. *Journal of Nursing Scholarship,* 46(2):82-90.

Nobile, S., Di Sipio Morgia, C., Vento, G. (2022). Perinatal origins of adult disease and opportunities for health promotion: A narrative review. *Journal of Personalized Medicine*, 12(2):157.

Olson, D.H., Sprenkle, D.H., Russell, C.S. (1979). Circumplex model of marital and family systems: I. Cohesion and adaptability dimensions, family types, and clinical applications. *Family Process, 18*(1), 3-28.

Olson, D. H., Waldvogel, L., Schlieff, M. (2019). Circumplex Model of marital and family systems: An update. *Journal of Family Therapy and Review*, *11*, 199-211.

Olson, D. H. (2000).Circumplex model of marital and family systems. *Journal of Family Therapy*, *22*(2), 144-167.

Panceri, C., Valentini, N.C., Silveira, R.C., *et al.* (2020). Neonatal adverse outcomes, neonatal birth risks, and socioeconomic status: Combined influence on preterm infants' cognitive, language, and motor development in Brazil. *Journal of Child Neurology,* 35(14):989-998.

Papadakaki, M., Stamouli, M-A., Chliaoutakis, J. (2021). Exploring the psychosocial needs of people living in extreme poverty and introducing brief interventions: The case of Crete region in Greece. *Research on Social Work Practice,* 3(4), 410-420.

Parker, S.J., Zahr, L.K., Cole, J.G., *et al.* (1992). Outcome after developmental intervention in the neonatal intensive care unit for mothers of preterm infants with low socioeconomic status. *Journal of Pediatrics,* 120(5):780-5.

Parlapani, E., Holeva, V., Voitsidis, P., *et al.* (2020) Psychological and Behavioral Responses to the COVID-19 Pandemic in Greece. *Frontiers in Psychiatry*, 11:821. Peuhkuri, K., Sihvola, N., & Korpela, R. (2012). Dietary factors and fluctuating levels of melatonin. *Food and Nutrition Research*, 56: 17252.

Porges, S. W., & Furman, S. A. (2011). The early development of the autonomic nervous system provides a neural platform for social behavior: A polyvagal perspective. *Infant and Child Development, 20*(1), 106-118.

Pizzi, C., Richiardi, M., Charles., M-A., *et al.* (2020). Measuring child socio-economic position in birth cohort research: The development of a novel standardized household income indicator. *International Journal of Environmental Research and Public Health*, 17(5), 1700.

Porter, C. L., Wouden-Miller, M., Silva, S. S., *et al.* (2003) Marital harmony and conflict: Linked to infants' emotional regulation and cardiac vagal tone. *Infancy, 4*(2), 297-307.

Potijk, M.R., de Winter, A.F., Bos, A.F., *et al.* (2012). Higher rates of behavioural and emotional problems at preschool age in children born moderately preterm. *Archives of Disease in Childhood*, 97(2):112-7.

Pravia, C.I., Benny, M. (2020). Long-term consequences of prematurity. *Cleveland Clinic Journal of Medicine*, 23;87(12):759-767.

Prom, M.C., Denduluri, A., Philpotts, L.L., *et al.* (2022). A Systematic Review of Interventions That Integrate Perinatal Mental Health Care Into Routine Maternal Care in Low and Middle-Income Countries. *Frontiers in Psychiatry* 13:859341.

Provenzi, L., Borgatti, R., Montirosso, R. (2017). Why are prospective longitudinal studies needed in preterm behavioral epigenetic research? *JAMA Pediatrics,* 171(1), 92. Qin,

Y., Shi, W., Zhuang, J. *et al.* (2019) Variations in melatonin levels in preterm and term human breast milk during the first month after delivery. *Scientific Reports,* 9, 17984.

Reiss, F., Meyrose, A.K., Otto, C., *et al.* (2019). Socioeconomic status, stressful life situations and mental health problems in children and adolescents: Results of the German BELLA cohort-study. *PLoS One,* 14(3):e0213700.

Roussi, P., Karademas, E. C. (2016). Dyadic coping in Greek couples. *Dyadic Coping: International Perspectives,* 153.

Samuel, T.M., Zhou, Q., Giuffrida, F., *et al.* (2020) Nutritional and non-nutritional composition of human milk Is modulated by maternal, infant, and methodological factors. *Frontiers in Nutrition,* 7:576133.

Scorza, P., Monk, C., Lee, S., *et al.* (2020). Preventing maternal mental health disorders in the context of poverty: pilot efficacy of a dyadic intervention. *American Journal of Obstetrics and Gynecology MFM*, 2(4):100230.

Shigeto, A., Mangelsdorf, S. C., Brown, G. J. (2013). Roles of family cohesiveness, marital adjustment and child temperament in predicting child behavior with mothers and fathers. *Journal of Social and Personal Relationships*, 31(2), 200-220.

Singer, L.T., Fulton, S., Davillier, M., *et al.* (2003). Effects of infant risk status and maternal psychological distress on maternal-infant interactions during the first year of life. *Journal of Developmental and Behavioral Pediatrics,* 24(4), 233–241.

Singletary, B., Bates, R., Justice, L. (2021). Evaluating associations between maternal social support and cognitive development for infants in poverty. *Infant Behavior and Development,* 63: 101546

Spielberger, C.D., Gorsuch, R.L., Lushene, R. (1983) State-Trait Anxiety Inventory for Adults: Sampler Set, Manual, Instrument and Scoring Guide. Consulting Psychologists Press, Palo Alto, CA, 1983.

Suga, A., Uraguchi, M., Tange, A., *et al.* (2019). Cardiac interaction between mother and infant: enhancement of heart rate variability. *Scientific Reports, 9*(1), 1-9.

Sullivan, M.C., Winchester, S.B., Bryce, C.I., *et al.* (2017). Prematurity and perinatal adversity effects hypothalamic-pituitary-adrenal axis reactivity to social evaluative threat in adulthood. *Developmental Psychobiology*, 59(8):976.

Tauman, R., Zisapel, N., Laudon, M., *et al.* (2002). Melatonin production in infants. *Pediatric Neurology,* 26(5):379-82.

Theofilou, P. (2015). Translation and cultural adaptation of Multidimensional Scale of Perceived Social Support for Greece. *Health Psychology Research, 3*(1061), 9-11. Tissot, H., Lapalus, N., Frascarolo, F. *et al.* (2022). Family alliance in infancy and toddlerhood predicts social cognition in adolescence. *Journal of Child and Family Studies*, 31, 1338-1349.

Tordjman, S., Chokron, S., Delorme, R., *et al.* (2017). Melatonin: Pharmacology, functions and therapeutic benefits. *Current Neuropharmacology*, 15(3):434-443. Trevarthen, C. (2001). Intrinsic motives for companionship in understanding: Their origin, development, and significance for infant mental health. *Infant Mental Health Journal*, *22*(1–2), 95–131.

Tripathy, P. (2020). A public health approach to perinatal mental health: Improving health and wellbeing of mothers and babies. *Journal of Gynecology, Obstetrics and Human Reproduction*, 49(6):101747.

Tsobanoglou, G.O. (2014). The Eurozone crisis: social factors and impacts, emergent sociality and community employment capacity building. In N.P. Petropoulos, & G.O. Tsobanoglou (Eds.), *The Debt Crisis in the Eurozone: Social Impacts* (pp. 396–424). Cambridge Scholars Publishing. Van Haeken, S., Braeken, M.AK.A., Nuyts, T., *et al.*

(2020). Perinatal Resilience for the First 1,000 Days of Life. Concept Analysis and Delphi Survey. *Frontiers in Psychology*, 11:563432.
Van der Hulst, M., Polinder, S., Kok, R., *et al.* (2022). Socio-economic determinants of healthcare costs in early life: a register-based study in the Netherlands. *International Journal of Equity in Health*, 21(1):5.
Van der Merwe, S.E., Biggs, R., Preiser, R., *et al.* (2019). Making Sense of Complexity: Using SenseMaker as a Research Tool. *Systems*, *7*, 25.
Vatavali, F.; Gareiou, Z.; Kehagia, F.; *et al.* (2020). Impact of COVID-19 on Urban Everyday Life in Greece. Perceptions, Experiences and Practices of the Active Population. *Sustainability*, *12*, 9410.
Vavoura, C., Vavouras, I. S. (2022). Income inequality and poverty in Greece during the recent economic, fiscal and Covid-19 crises. *Social Cohesion and Development*, 17(1), 5-21.
Vivilaki, V.G., Dafermos, V., Kogevinas, M., *et al.* (2009). The Edinburgh Postnatal Depression Scale: translation and validation for a Greek sample. *BMC Public Health*, 9:329.
Vlachadis, N., Kornarou, E., Ktenas, E. (2013). The preterm births epidemic in Greece. *Acta Obstetricia et Gynecologica Scandinavica,* 92(10):1231.
Weyers, S., Dragano, N., Möbus, S. *et al.* (2008). Low socio-economic position is associated with poor social networks and social support: results from the Heinz Nixdorf Recall Study. *International Journal of Equity in Health,* 7, 13.
Widding, U.; Farooqi, A. (2016). I thought he was ugly: Mothers of extremely premature children narrate their experiences as troubled subjects. *Feminism and Psychology*, 26, 153–169.
Wille, D. E (1991). Relation of preterm birth with quality of infant-mother attachment at one year.
Infant Behavior and Development, 14, 227-240.
Willig, C. (2001). *Introducing Qualitative Research in Psychology. Adventures in theory and method.* United Kingdom: Open University Press.
Winstone LK, Luecken LJ, Crnic KA, *et al.* (2020). Patterns of family negativity in the perinatal period: Implications for mental health among Mexican-origin women. *Journal of Family Psychology*, 34(5):642-651.
Wong, H.S., Edwards, P. (2013). Nature or nurture: a systematic review of the effect of socio-economic status on the developmental and cognitive outcomes of children born preterm. *Maternal and Child Health Journal*, 17(9):1689.
Youngblut, J.M., Loveland-Cherry, C.J., Horan, M. (1994). Maternal employment effects on families and preterm infants at 18 months. *Nursing Research*, 43(6):331-7. Zimet, G.D., Dahlem, N.W., Zimet, S.G., *et al.* (1988). The Multidimensional Scale of Perceived Social Support. *Journal of Personality Assessment, 52*(1), 30-41.
