# Supplementary material for: The development of preterm infants from low socio-economic status families: The combined effects of melatonin, autonomic nervous system maturation and psychosocial factors (ProMote): A study protocol
Source: PLoS One. 2025 Jan 10;20(1):e0316520. doi: 10.1371/journal.pone.0316520 (PMC11723634; doi:10.1371/journal.pone.0316520)
Supplement: S3 File — (PDF) [file pone.0316520.s003.pdf]

ΕΝΗΜΕΡΩΣΗ

ΕΡΕΥΝΗΤΙΚΟ ΕΡΓΟ ProMote (ΚΑ 11455)

ΕΠΙΣΤΗΜΟΝΙΚΑ ΥΠΕΥΘΥΝΗ: ΘΕΑΝΩ ΚΟΚΚΙΝΑΚΗ

Στον Κανονισμό Λειτουργίας της Επιτροπής Ηθικής και Δεοντολογίας της Έρευνας του Πανεπιστημίου Κρήτης, και ειδικότερα στο ΚΕΦΑΛΑΙΟ Β: ΑΡΜΟΔΙΟΤΗΤΕΣ – ΔΙΑΔΙΚΑΣΙΕΣ ΑΞΙΟΛΟΓΗΣΗΣ & ΕΓΚΡΙΣΗΣ, στο Άρθρο 5 *Αρμοδιότητες*, αναφέρονται τα εξής:

Αρμοδιότητα της Ε.Η.Δ.Ε. είναι να διαπιστώνει κατά πόσον συγκεκριμένο ερευνητικό έργο που πρόκειται να εκπονηθεί στο Π.Κ. δεν αντιβαίνει στην κείμενη νομοθεσία και κατά πόσον συνάδει με γενικά παραδεδεγμένους κανόνες ηθικής και δεοντολογίας της έρευνας και ερευνητικής ακεραιότητας ως προς το περιεχόμενο και προς τον τρόπο διεξαγωγής της. Ειδικότερα στις αρμοδιότητες της Ε.Η.Δ.Ε. περιλαμβάνονται:

....

γ) Η παρακολούθηση προτεινόμενων αλλαγών σε ήδη εγκεκριμένες και υλοποιούμενες έρευνες.

Μέσα σε αυτό το πλαίσιο και σε συνέχεια της εγκεκριμένης αίτησης στην Επιτροπή Ηθικής και Δεοντολογίας του Πανεπιστημίου Κρήτης (αριθμός και ημερομηνία απόφασης ΕΗΔΕ: 103/22.09.2023) για την διεξαγωγή της μελέτης ProMote (ΕΛ.ΙΔ.Ε.Κ.), ως Επιστημονικά Υπεύθυνη του έργου παρακάτω σας ενημερώνω για τα εξής:

Α. Ενημέρωση για τα αρχικά μη ονοματισμένα μέλη της ερευνητικής ομάδας

Η κ. *Μαρία Μαρκοδημητράκη*, Αναπληρώτρια Καθηγήτρια Αναπτυξιακής Ψυχολογίας Βρεφών και Νηπίων (Παιδαγωγικό Τμήμα Προσχολικής Εκπαίδευσης, Πανεπιστήμιο Κρήτης) είναι η ψυχολόγος που έχει συμπεριληφθεί στην ερευνητική ομάδα προκειμένου να συμβάλλει στην προσέγγιση των μητέρων της μελέτης ProMote και να συμμετάσχει στη συλλογή των δεδομένων που αφορούν τους ψυχοκοινωνικούς παράγοντες και στη μεταβλητότητα καρδιακού ρυθμού καθώς και στην χορήγηση των *Bayley Scales of Infant and Toddler Development, 3rd Edition* (Bayley, 2006) στους 9 μήνες (διορθωμένη ηλικία) για όλους τους συμμετέχοντες στην έρευνα. Επισυνάπτεται η σχετική δήλωση εχεμύθειας για την προστασία δεδομένων προσωπικού χαρακτήρα και την εμπιστευτικότητα, η υπογεγραμμένη από την ίδια ενότητα Α6 της αίτησης προς την Ε.Η.Δ.Ε. καθώς και η σύμβαση πρόσθετης απασχόλησης της κ. Μ. Μαρκοδημητράκη.

Ο κ. *Αλκιβιάδης Σαββάκης* είναι ο τεχνικός βοηθός που έχει συμπεριληφθεί στην ερευνητική ομάδα προκειμένου να συμμετάσχει στην κατασκευή και διατήρηση της ιστοσελίδας της μελέτης ProMote καθώς και σε όλες τις δράσεις που σχετίζονται με

τη δημοσιοποίηση των αποτελεσμάτων της έρευνας. Επισυνάπτεται η σχετική δήλωση εχεμύθειας για την προστασία δεδομένων προσωπικού χαρακτήρα και την εμπιστευτικότητα, η υπογεγραμμένη από τον ίδιο ενότητα Α6 της αίτησης προς την Ε.Η.Δ.Ε. καθώς και η σύμβαση ανάθεσης έργου του κ. Α. Σαββάκη.

Β. Συμβάσεις πρόσθετης απασχόλησης και ανάθεσης έργου

Σύμφωνα με τα αναφερθέντα στην ενότητα Ε2 της αίτησης προς την Ε.Η.Δ.Ε. για την έγκριση της μελέτης, επισυνάπτονται οι συμβάσεις πρόσθετης απασχόλησης των κ.κ. Χατζηδάκη, Ε. και Κοκκινάκη, Θ. καθώς και οι συμβάσεις ανάθεσης έργου των κ.κ. Ρουμελιωτάκη, Θ. και Αναγνωστάτου, Ν. Κατά την παρούσα φάση της μελέτης, δεν έχουν υπογραφεί συμβάσεις για τα υπόλοιπα μέλη της ερευνητικής ομάδας.

Γ. Ενημέρωση για τα ερωτηματολόγια αξιολόγησης των ψυχοκοινωνικών παραγόντων που θα χρησιμοποιηθούν κατά τη διεξαγωγή της μελέτης ProMote

Επισυνάπτονται τα ερωτηματολόγια που θα χρησιμοποιηθούν για την αξιολόγηση των ψυχοκοινωνικών παραγώντων κατά τη διεξαγωγή της Α φάσης της μελέτης ProMote, (τα συγκεκριμένα ερωτηματολόγια συνόδευαν την αρχική αίτηση στην Ε.Η.Δ.Ε.), δηλαδή η ελληνική εκδοχή των ερωτηματολογίων *Edinburgh Postnatal Depression Scale* (EPDS, Cox, 1987), the *Spielberger State-Trait Anxiety Inventory for Adults* (STAI, Spielberger, 1983; Giannakou & Liakos, 1984), the *Family Adaptability and Cohesion Evaluation Scales IV Package* (FACES IV; Olson, 1979; Olson, 2019; Koutra, 2013), the *Multidimensional Scale of Perceived Social Support* (MSPSS, Zimet, 1988; Theofilou, 2015), και the *Dyadic Coping Inventory* (DCI, Bodenmann, 2008; Ledermann, 2010; Roussi & Karademas, 2016).

Τα ερωτηματολόγια που θα αναπτυχθούν κατά τη διάρκεια της Β' φάσης της μελέτης θα κατατεθούν κατά το επόμενο χρονικό διάστημα στην Ε.Η.Δ.Ε. για έγκριση.

Δ. Ενημέρωση και τεκμηρίωση της προσθήκης μετρήσεων της μεταβλητότητας του καρδιακού ρυθμού των νεογνών και προσδιορισμός του χρόνου μετρήσεων της μεταβλητότητας του καρδιακού ρυθμού των μητέρων

Η αρχική ερευνητική πρόταση προέβλεπε μια μέτρηση της νεογνικής μεταβλητότητας του καρδιακού ρυθμού (HRV) κατά την γέννηση. Ωστόσο, η προσεκτική ανασκόπηση της σύγχρονης βιβλιογραφίας για τον ακριβή προσδιορισμό του χρόνου μέτρησης των νεογνικών HRV υποδεικνύει ότι οι μετρήσεις HRV διαφοροποιούνται κατά τις πρώτες ώρες/ημέρες της ζωής τους. Για το λόγο αυτό οι νεογνικές μετρήσεις HRV προγραμματίζονται ως εξής:

1. Η πρώτη καταγραφή του νεογνικού HRV θα γίνει εντός 24 ωρών από τον τοκετό.
2. Μεταξύ 3ης και 4ης ημέρας μετά τον τοκετό θα γίνεται η δεύτερη καταγραφή του νεογνικού HRV.
3. Μόνο για τα νεογνά που γεννιούνται πριν τις 35 εβδομάδες θα γίνεται μια 3η καταγραφή του νεογνικού HRV περίπου στις 35-36 εβδομάδες μετά την πρώτη ημέρα της τελευταίας έμμηνης ρύσης.

Ακολουθεί η τεκμηρίωση για την αναγκαιότητα των παραπάνω μετρήσεων καθώς και ο προσδιορισμός των συνθηκών των μετρήσεων αυτών με βάση την σύγχρονη διεθνή βιβλιογραφία:

<u>Timeline and conditions of neonates' HRV measurements:</u>

Timeline of neonates HRV measurements:
Postnatal age at the time of HRV testing may contribute to differences in early autonomic tone since there is evidence for a postnatal *transitional period of maturation of the ANS, cardiovascular, and respiratory systems*. This transitional period extends for a few days beyond delivery resulting in a maturational increase in HRV metrics when ANS tone is evaluated both within a few hours of birth and at three to four days of age. Thus, it is possible that there may be immediate, but limited effects on infant HRV according to both the circumstances of birth and the timing of testing (Mulkey et al., 2019).

Although there is evidence (coming from long and short HRV measures) of an increase in cardiac autonomic modulation in term newborns after the first 24 h of life, a limited number of studies investigated cardiac autonomic modulation immediately after birth and its changes within the first hours in the extrauterine life (Shayani et al., 2019). *For preterm neonates* with a low level of medical morbidity, the duration of extrauterine development does not significantly impact ANS developmental trajectory from birth to NICU discharge (Mulkey et al., 2020 cited by Schlatterer et al., 2021). No increase in the parasympathetic measures were evidenced for premature neonates born from 28 to 32 weeks when measured throughout a follow up period (32-35 weeks PMA) (Hadas et al., 2021).

Meanwhile, a significant effect of postnatal age [comparing 3rd to 4th postnatal day vs early after birth (1st-2nd hour after birth)] revealed that the mean RR interval was significantly longer (increases in HRV) on the third to fourth postnatal day *of term neonates,* regardless the mode of delivery. This indicates cardiac autonomic maturation within the third to fourth postnatal day in spontaneously delivered and surgically delivered neonates. (Kozar et al., 2018). In connection to this, within 14 h after birth of term (male) neonates (comparison between 2h and 14h after birth, that is 12h after the first measurement) born of elective cesarean delivery, the mean of the iRRs increased as well as parasympathetic indices (Shayani et al., 2019). Previous studies using short recording methods had documented that in a few days after birth (from the 2nd to the 4th day after birth), there is an increase in the HRV indices in healthy term infants. During the first three days of life, different authors verified a gradual increase of the parasympathetic portion and a simultaneous reduction of sympathetic activity. Thus, studies suggest that HRV increases gradually during the first three days of life (see Shayani et al., 2019 for more information).

Mode of delivery has to be taken into consideration in neonates' HRV analysis since, on the one hand, overall ANS tone is not altered by mode of delivery in low-risk term newborns (Mulkey et al., 2019) but, on the other hand, newborns born by vaginal delivery without analgesia (VD group) are characterized by significantly higher HF% compared to surgically delivered neonates (CS group) and these differences are resolved by the third to fourth postnatal day (Kozar et al., 2018). In connection to this, another study, comparing the HRV between spontaneously and surgically born

newborns, showed decreased HRV in neonates delivered by caesarean section within three days postpartum (Sheen et al., 2014, cited by Kozar et al., 2018). Further, *prematurity-associated morbidities* will be taken into account in neonates' HRV analysis since they correlate with autonomic development of premature infants and may have a greater impact on this system extrauterine maturation than birth gestational age (Schlatterer et al., 2022).

Based on the above mentioned recent evident, three measurements for neonates' HRV measurements will be carried out:

- The first measurement will be performed within 24 hours after birth (in the supine position in the incubator),
- The second measurement will be performed on the third to fourth postnatal day after birth (in the neonatal cot covered with a blanket).
- Only for premature neonates born before 35 gestation weeks, a third measurement will be carried out at around 35-36 weeks PMA.

Conditions of neonates' HRV measurement: All of the recordings will be obtained during quiet sleep, identified through physiological and behavior monitoring, **30 min or 1h** after a morning-time feeding period (between 8.00 and 12.00 a.m.) to minimize its effect on HRV (Kozar et al., 2018), without painful or stressful procedures for at least 6 hours. All of the neonates will be in supine position during the recordings. Recordings will be delayed by 48 hours in cases of unstable/unpredicted acute pathology, or administration of drugs with cardiac effects in the 7 days preceding the recording (Nguyen Phuc Thu et al., 2019).

Duration: 10-minute time length of HRV measurements provides a good compromise with minimal error for all features estimations (Nguyen Phuc Thu et al., 2019).

Επίσης, η αρχική ερευνητική πρόταση προέβλεπε μια μέτρηση της μητρικής μεταβλητότητας του καρδιακού ρυθμού (HRV) κατά την γέννηση. Η προσεκτική ανασκόπηση της σύγχρονης βιβλιογραφίας για τον ακριβή προσδιορισμό του χρόνου μέτρησης των μητέρων υποδεικνύει ότι οι μετρήσεις της μητρικής HRV δεν επανέρχονται σε επίπεδα όμοια με εκείνα πριν της εγκυμοσύνης κατά το διάστημα 2 εβδομάδων μεταγεννητικά. Επιπλέον, οι μετρήσεις της μητρικής HRV επηρεάζονται από τον πόνο. Οι μετρήσεις της μητρικής HRV προγραμματίζονται για την 3η έως την 6^{η} ημέρα μετά τον τοκετό επειδή οι μητέρες νοσηλεύονται ακόμα στη Μαιευτική Κλινική του Πανεπιστημιακού Γενικού Νοσοκομείου Ηρακλείου ενώ τα επίπεδα πόνου μετά την καισαρική τομή δεν διαφοροποιούνται σημαντικά τις πρώτες ημέρες μεταγεννητικά. Λόγω της συσχέτισης των μετρήσεων HRV με τον πόνο, μια αυτο-αξιολόγηση του πόνου των μητέρων μέσω μιας απλής αριθμητικής κλίμακας θα προηγείται της μέτρησης της μητρικής HRV.

Ακολουθεί η τεκμηρίωση για την αναγκαιότητα των παραπάνω μετρήσεων καθώς και ο προσδιορισμός των συνθηκών των μετρήσεων αυτών με βάση την σύγχρονη διεθνή βιβλιογραφία:

Maternal HRV measurements

<u>Maternal HRV recovery</u>: Sympathetic nervous system activity increases while parasympathetic activity normatively decreases across pregnancy (Brown et al.,

2021). However, the extent and the timing of cardiac recovery have been a subject of debate.

Rowan et al (2022) showed that at 49 days prior to birth there is a reversal of HRV indices with a steady increase in daily HRV that continued in the postpartum period. Meanwhile, heart rate (HR) was reported to return slowly to baseline levels by 2–6 weeks postpartum in some studies [for a review see in Chen et al., 2016]. Chen et al. (2016) showed that the pulse rate declined shortly after delivery and reached a relatively low level but it did not return to normal within 6 weeks. Conversely, it increased from the 7th week to the 11th week postpartum. Meanwhile, there is some evidence to suggest that vagally-mediated HRV returns to pre-pregnant levels within three months (Chen et al., 1999 cited in Brown et al., 2021). This is consistent with evidence showing that HRV parameters return to normal within three months after delivery (Sarhaddi et al., 2022). In one report, the recovery period was around 20 weeks. In other studies, a continued decrease in cardiac output was observed to last over the next 24 weeks (see in Chen et al., 2016 for a review). Further, Brown et al (2021) showed that vagally-mediated HRV increased between 3rd trimester and 4-6 weeks postpartum. Other studies indicated that cardiac activities might not return to original levels even after 1 year. However, there has also been concern that the changes in cardiac function associated with pregnancy might not ultimately return to pre-pregnancy levels [for a review see in Chen et al., 2016].

The effect of partner relationship (through dyadic coping) and mental health on the recovery period will be taken into consideration in mothers' HRV analysis (Brown et al., 2021).

Maternal HRV and labor pain: Labor is associated with significant physiological changes. In the course of labor, there are continuous adjustments of cardiac autonomic reflexes by alternate activations of the sympathetic and parasympathetic nervous systems (Musa et al., 2017). In connection to this, a relationship between autonomic nervous system indexed by HRV and the pain response has been confirmed (Forte et al., 2021). Further, labor pain intensity is known to predict persistent postpartum pain. After the delivery, uterine contraction pain is common within 48 hours of delivery and postpartum pain may last between four weeks to three months (Tan et al., 2023). Taken together, maternal postpartum pain experience may persist for days or weeks after birth and may affect maternal HRV measurements in the course of the first days after delivery.

Conditions:

The above literature review shows that possibly HRV values do not recover before the 2nd week postpartum and maternal postpartum pain experience may persist for days or weeks after birth and may affect maternal HRV measurements in the course of the first days after delivery. On this ground:

A) Assessment of maternal postpartum pain according to a numeric rating scale will precede maternal HRV measurements,

B) maternal HRV measurements will be carried out between the 3rd and 6th day postpartum.

In particular, HRV will be continuously measured for 5 minutes noninvasively. All participants will be in a sitting position (Brown et al., 2021). HRV was measured in this study in the morning between 11.00 am and 2.00 p.m and after 1- 2 h after a morning time-feeding hour.

E. Ενημέρωση και τεκμηρίωση του χρόνου άντλησης του μητρικού γάλατος

Στην αρχική ερευνητική πρόταση το μητρικό γάλα θα προέρχονταν από άντληση που θα γινόταν μεταξύ 10:00 π.μ.-14:00 μ.μ. Ωστόσο, η ανασκόπηση της σύγχρονης βιβλιογραφίας δείχνει οτι τα υψηλότερα επίπεδα μελατονίνης εντοπίζονται στο μητρικό γάλα γύρω στις 3.00 π.μ. Για το λόγο αυτό θα ζητηθεί από τις μητέρες το γάλα που έχουν αντλήσει μεταξύ 01:00 και 05:00 π.μ.

Ακολουθεί η τεκμηρίωση για την παραπάνω τροποποίηση:
Melatonin has been detected in breastmilk of human beings. Similar as the fluctuations in plasma, that is relatively low during daytime and relatively high at night, melatonin levels in milk also shows a circadian rhythm. Melatonin concentrations of night milking are approximately ten times compared to that in the daytime. In both preterm and term breastmilk, the melatonin concentration presented a circadian rhythm with the acrophase at around 03:00 (Qin et al., 2019). Melatonin has been found in colostrum with the comparable concentration as it in plasma (Meng et al., 2017). On this ground, mothers of preterm neonates will be asked to collect breastmilk between 01:00-05:00 a.m. at the three above mentioned time periods.

Βιβλιογραφία

Brown RL, Fagundes CP, Thayer JF, Christian LM. Longitudinal changes in HRV across pregnancy and postpartum: Effect of negative partner relationship qualities. Psychoneuroendocrinology. 2021 Jul;129:105216. doi: 10.1016/j.psyneuen.2021.105216

Chen, Y. et al. (2016) Long-term measurement of maternal pulse rate dynamics using a home-based sleep monitoring system. *Journal of Sensors*, Article ID 5730142, https://doi.org/10.1155/2016/5730142

Hadas IM, Joseph M, Luba Z, Michal KL. Assessing parasympathetic measures of heart rate variability shortly after birth to predict motor repertoire at four months in low risk preterm infants born between 28 and 32 weeks of gestation. Early Hum Dev. 2021 Oct;161:105438. doi: 10.1016/j.earlhumdev.2021.105438

Kozar M, Tonhajzerova I, Mestanik M, Matasova K, Zibolen M, Calkovska A, Javorka K. Heart rate variability in healthy term newborns is related to delivery mode: a prospective observational study. BMC Pregnancy Childbirth. 2018 Jun 27;18(1):264. doi: 10.1186/s12884-018-1900-4.

Musa Shaza M., Adam Ishag, Hassan Nada G., Rayis Duria A., Lutfi Mohamed F. (2017). Maternal Heart Rate Variability during the First Stage of Labor. Frontiers in Physiology, 8 , https://www.frontiersin.org/articles/10.3389/fphys.2017.00774, doi:10.3389/fphys.2017.00774

Meng X, Li Y, Li S, Zhou Y, Gan RY, Xu DP, Li HB. Dietary Sources and Bioactivities of Melatonin. Nutrients. 2017 Apr 7;9(4):367. doi: 10.3390/nu9040367

Mulkey, S. B., du Plessis, A. J. (2019). Autonomic system development and its impact on neuropsychiatric outcome. *Pediatric Research, 85*(2), 120-126

Nguyen Phuc Thu T, Hernández AI, Costet N, Patural H, Pichot V, et al. (2019) Improving methodology in heart rate variability analysis for the premature infants: Impact of the time length. PLOS ONE 14(8): e0220692. https://doi.org/10.1371/journal.pone.0220692

Peuhkuri, K., Sihvola, N., & Korpela, R. (2012). Dietary factors and fluctuating levels of melatonin. *Food and Nutrition Research*, 56: 17252.

Qin, Y., Shi, W., Zhuang, J. *et al.* (2019) Variations in melatonin levels in preterm and term human breast milk during the first month after delivery. *Scientific Reports*, 9, 17984.

Rowan SP, Lilly CL, Claydon EA, Wallace J, Merryman K. Monitoring one heart to help two: heart rate variability and resting heart rate using wearable technology in active women across the perinatal period. BMC Pregnancy Childbirth. 2022 Nov 30;22(1):887. doi: 10.1186/s12884-022-05183-z.

Sarhaddi F, Azimi I, Axelin A, Niela-Vilen H, Liljeberg P, Rahmani AM. Trends in Heart Rate and Heart Rate Variability During Pregnancy and the 3-Month Postpartum Period: Continuous Monitoring in a Free-living Context. JMIR Mhealth Uhealth. 2022 Jun 3;10(6):e33458. doi: 10.2196/33458.

Schlatterer SD, Govindan RB, Barnett SD, Al-Shargabi T, Reich DA, Iyer S, Hitchings L, Larry Maxwell G, Baker R, du Plessis AJ, Mulkey SB. Autonomic development in preterm infants is associated with morbidity of prematurity. Pediatr Res. 2022 Jan;91(1):171-177. doi: 10.1038/s41390-021-01420-x

Tan CW, Tan NY, Sultana R, Tan HS, Sng BL. Investigating the association factors of acute postpartum pain: a cohort study. BMC Anesthesiol. 2023 Jul 25;23(1):252. doi: 10.1186/s12871-023-02214-w.
