# Supplementary material for: The development of preterm infants from low socio-economic status families: The combined effects of melatonin, autonomic nervous system maturation and psychosocial factors (ProMote): A study protocol
Source: PLoS One. 2025 Jan 10;20(1):e0316520. doi: 10.1371/journal.pone.0316520 (PMC11723634; doi:10.1371/journal.pone.0316520)
Supplement: S4 File — (PDF) [file pone.0316520.s004.pdf]

UPDATE

RESEARCH PROJECT ProMote (KA11455)

SCIENTIFIC RESPONSIBLE: THEANO KOKKINAKI

In the Operating Regulations of the Ethics Committee of the University of Crete (E.H.Δ.E), and in particular in CHAPTER B: COMPETENCES – EVALUATION & APPROVAL, Article 5, Competences, the following are mentioned:

Competence of E.H.Δ.E. is to ascertain whether a specific research project which will be implemented in the University of Crete does not contravene current legislation and whether it is consistent with generally accepted rules of ethics and ethics research and research integrity in content and manner of its implementation. In particular, the responsibilities of E.H.Δ.E. include:
……………………………………………………………………………………………..
c) Monitoring proposed changes to already approved and implemented research.

Within this context and following the approved application to the Ethics Committee of the University of Crete (number and date E.H.Δ.E. decision: 103/22.09.2023) for the implementation of ProMote study (H.F.R.I.), as Scientific Responsible of the project below, I inform you about the following:

A. Update on the unnamed members of the research team

Mrs Maria Markodimitraki, Associate Professor of Developmental Psychology of Infants and Toddlers (Department of Preschool Education, University Crete) is the psychologist who has been included in the research team in order to help reach the mothers of the ProMote study and to participate in the collection of data related to psychosocial factors, in both heart rate variability measurements and administration of the *Bayley Scales of Infant and Toddler Development, 3rd Edition* (Bayley, 2006) at 9 months (corrected age) for all research participants. Attached you may find the relevant data protection and confidentiality statement, the signed by her section A6 of the application to the E.H.Δ.E. as well as the additional employment contract of M. Markodimitraki.

Mr Alkiviadis Savvakis is the technical assistant who has been included in research team in order to participate in its construction and maintenance website of the ProMote study as well as in all actions related to the dissemination of research results. Attached you may find the relevant confidentiality and data protection statement, the signed by him section A6 of the application to E.H.Δ.E. as well as the project assignment contract of Mr. A. Savvaki.

B. Additional employment and project assignment contracts

According to what was mentioned in section E2 of the application to E.H.Δ.E. for the approval of the study, attached you may find the additional employment and project assignment contracts of E. Hatzidaki and Th. Kokkinaki as well and the project assignment contracts of Th. Roumeliotaki and N. Anagnostatou. At the present stage of

the study, no contracts have been signed for the remaining members of the research team.

C. Update on the questionnaires for the assessment of psychosocial factors that will be used in the course of ProMote study

Attached you may find the questionnaires that will be used for the assessment of psychosocial factors during the stage A of ProMote study, (the specific questionnaires accompanied the original application to E.H.Δ.E.), i.e. the Greek version of the questionnaires *Edinburgh Postnatal Depression Scale* (EPDS, Cox, 1987), the *Spielberger State-Trait Anxiety Inventory for Adults* (STAI, Spielberger, 1983; Giannakou & Liakos, 1984), the *Family Adaptability and Cohesion Evaluation Scales IV Package* (FACES IV; Olson, 1979; Olson, 2019; Koutra, 2013), the *Multidimensional Scale of Perceived Social Support* (MSPSS, Zimet, 1988; Theofilou, 2015) and the *Dyadic Coping Inventory* (DCI, Bodenmann, 2008; Ledermann, 2010; Roussi & Karademas, 2016).

The questionnaires that will be used during stage B of the study will be submitted in the next period to E.H.Δ.E. for approval.

D. Update and documentation of additional neonatal heart rate variability measurements and determination of the time of measurements of maternal heart rate variability

The original research proposal predicted one measurement of neonatal heart rate variability (HRV) at birth. However, the careful review of recent literature for the precise determination of neonatal HRV measurement time indicates that HRV measurements differ during the first hours/days of life. For this reason, neonatal HRV measurements are scheduled as follows:

1.The first recording of neonatal HRV will be done within 24 hours of childbirth.
2. Between the 3rd and 4th day after delivery, the second neonatal HRV measurement will take place.
3. Only for newborns born before 35 weeks, a 3rd HRV measurement will be done at approximately 35-36 weeks after the first day of last menstrual period.

Documentation for the necessity of the above measurements as well as the determination of the conditions of these measurements based on the recent literature follows:

Timeline and conditions of neonates' HRV measurements:

Timeline of neonates HRV measurements:
Postnatal age at the time of HRV testing may contribute to differences in early autonomic tone since there is evidence for a postnatal *transitional period of maturation of the ANS, cardiovascular, and respiratory systems*. This transitional period extends for a few days beyond delivery resulting in a maturational increase in HRV metrics when ANS tone is evaluated both within a few hours of birth and at three to four days of age. Thus, it is possible that there may be immediate, but limited effects on infant HRV according to both the circumstances of birth and the timing of testing (Mulkey et al., 2019).

Although there is evidence (coming from long and short HRV measures) of an increase in cardiac autonomic modulation in term newborns after the first 24 h of life, a limited number of studies investigated cardiac autonomic modulation immediately after birth and its changes within the first hours in the extrauterine life (Shayani et al., 2019). *For preterm neonates* with a low level of medical morbidity, the duration of extrauterine development does not significantly impact ANS developmental trajectory from birth to NICU discharge (Mulkey et al., 2020 cited by Schlatterer et al., 2021). No increase in the parasympathetic measures were evidenced for premature neonates born from 28 to 32 weeks when measured throughout a follow up period (32-35 weeks PMA) (Hadas et al., 2021).

Meanwhile, a significant effect of postnatal age [comparing 3rd to 4th postnatal day vs early after birth (1st-2nd hour after birth)] revealed that the mean RR interval was significantly longer (increases in HRV) on the third to fourth postnatal day *of term neonates,* regardless the mode of delivery. This indicates cardiac autonomic maturation within the third to fourth postnatal day in spontaneously delivered and surgically delivered neonates. (Kozar et al., 2018). In connection to this, within 14 h after birth of term (male) neonates (comparison between 2h and 14h after birth, that is 12h after the first measurement) born of elective cesarean delivery, the mean of the iRRs increased as well as parasympathetic indices (Shayani et al., 2019). Previous studies using short recording methods had documented that in a few days after birth (from the 2nd to the 4th day after birth), there is an increase in the HRV indices in healthy term infants. During the first three days of life, different authors verified a gradual increase of the parasympathetic portion and a simultaneous reduction of sympathetic activity. Thus, studies suggest that HRV increases gradually during the first three days of life (see Shayani et al., 2019 for more information).

Mode of delivery has to be taken into consideration in neonates' HRV analysis since, on the one hand, overall ANS tone is not altered by mode of delivery in low-risk term newborns (Mulkey et al., 2019) but, on the other hand, newborns born by vaginal delivery without analgesia (VD group) are characterized by significantly higher HF% compared to surgically delivered neonates (CS group) and these differences are resolved by the third to fourth postnatal day (Kozar et al., 2018). In connection to this, another study, comparing the HRV between spontaneously and surgically born newborns, showed decreased HRV in neonates delivered by caesarean section within three days postpartum (Sheen et al., 2014, cited by Kozar et al., 2018). Further, *prematurity-associated morbidities* will be taken into account in neonates' HRV analysis since they correlate with autonomic development of premature infants and may have a greater impact on this system extrauterine maturation than birth gestational age (Schlatterer et al., 2022).

Based on the above mentioned recent evident, three measurements for neonates' HRV measurements will be carried out:

- The first measurement will be performed within 24 hours after birth (in the supine position in the incubator),
- The second measurement will be performed on the third to fourth postnatal day after birth (in the neonatal cot covered with a blanket).
- Only for premature neonates born before 35 gestation weeks, a third measurement will be carried out at around 35-36 weeks PMA.

Conditions of neonates' HRV measurement: All of the recordings will be obtained during quiet sleep, identified through physiological and behavior monitoring, **30 min or 1h** after a morning-time feeding period (between 8.00 and 12.00 a.m.) to minimize its effect on HRV (Kozar et al., 2018), without painful or stressful procedures for at least 6 hours. All of the neonates will be in supine position during the recordings. Recordings will be delayed by 48 hours in cases of unstable/unpredicted acute pathology, or administration of drugs with cardiac effects in the 7 days preceding the recording (Nguyen Phuc Thu et al., 2019).

Duration: 10-minute time length of HRV measurements provides a good compromise with minimal error for all features estimations (Nguyen Phuc Thu et al., 2019).

In addition, the initial research proposal predicted one heart rate variability measurement (HRV) after birth. The meticulous recent literature review for the precise determination of time measurement of mothers indicates that maternal HRV measurements do not return to pre-pregnancy levels during the interval of 2 weeks postpartum. In addition, measures of maternal HRV are affected by pain. Maternal HRV measurements are scheduled for the 3rd to 6th day after delivery because mothers are still hospitalized in Obstetrics Clinic of the University General Hospital of Heraklion while the levels of pain after caesarean section do not differ significantly in the first days postnatally. Because of the correlation of HRV measurements with pain, a self-assessment of mothers' pain through a simple numerical scale will precede maternal HRV measurement.

Documentation for the necessity of the above measurements as well as the determination of the conditions of these measurements based on recent literature follows:

Maternal HRV measurements

Maternal HRV recovery: Sympathetic nervous system activity increases while parasympathetic activity normatively decreases across pregnancy (Brown et al., 2021). However, the extent and the timing of cardiac recovery have been a subject of debate.

Rowan et al (2022) showed that at 49 days prior to birth there is a reversal of HRV indices with a steady increase in daily HRV that continued in the postpartum period. Meanwhile, heart rate (HR) was reported to return slowly to baseline levels by 2−6 weeks postpartum in some studies [for a review see in Chen et al., 2016]. Chen et al. (2016) showed that the pulse rate declined shortly after delivery and reached a relatively low level but it did not return to normal within 6 weeks. Conversely, it increased from the 7th week to the 11th week postpartum. Meanwhile, there is some evidence to suggest that vagally-mediated HRV returns to pre-pregnant levels within three months (Chen et al., 1999 cited in Brown et al., 2021). This is consistent with evidence showing that HRV parameters return to normal within three months after delivery (Sarhaddi et al., 2022). In one report, the recovery period was around 20 weeks. In other studies, a continued decrease in cardiac output was observed to last over the next 24 weeks (see in Chen et al., 2016 for a review). Further, Brown et al (2021) showed that vagally-mediated HRV increased between 3rd trimester and 4-6 weeks postpartum. Other studies indicated that cardiac activities might not return to original levels even after 1 year. However, there has also been concern that the changes in cardiac function associated with pregnancy might not ultimately return to pre-pregnancy levels [for a review see in Chen et al., 2016].

The effect of partner relationship (through dyadic coping) and mental health on the recovery period will be taken into consideration in mothers' HRV analysis (Brown et al., 2021).

Maternal HRV and labor pain: Labor is associated with significant physiological changes. In the course of labor, there are continuous adjustments of cardiac autonomic reflexes by alternate activations of the sympathetic and parasympathetic nervous

systems (Musa et al., 2017). In connection to this, a relationship between autonomic nervous system indexed by HRV and the pain response has been confirmed (Forte et al., 2021). Further, labor pain intensity is known to predict persistent postpartum pain. After the delivery, uterine contraction pain is common within 48 hours of delivery and postpartum pain may last between four weeks to three months (Tan et al., 2023). Taken together, maternal postpartum pain experience may persist for days or weeks after birth and may affect maternal HRV measurements in the course of the first days after delivery.

Conditions:
The above literature review shows that possibly HRV values do not recover before the 2nd week postpartum and maternal postpartum pain experience may persist for days or
weeks after birth and may affect maternal HRV measurements in the course of the first days after delivery. On this ground:

A) Assessment of maternal postpartum pain according to a numeric rating scale will precede maternal HRV measurements,

B) maternal HRV measurements will be carried out between the 3rd and 6th day postpartum.

In particular, HRV will be continuously measured for 5 minutes noninvasively. All participants will be in a sitting position (Brown et al., 2021). HRV was measured in this study in the morning between 11.00 am and 2.00 p.m and after 1- 2 h after a morning time-feeding hour.

E. Update and documentation of breast milk pumping time

In the original research proposal the mother's milk would come from pumping between 10:00 am-2:00 pm. However, the review of recent literature shows that the highest levels of melatonin are found in breast milk around 3.00 am. For this reason mothers will be asked to pump milk expressed between 01:00 and 05:00 a.m.

Documentation for the above modification follows:
Melatonin has been detected in breastmilk of human beings. Similar as the fluctuations in plasma, that is relatively low during daytime and relatively high at night, melatonin levels in milk also shows a circadian rhythm. Melatonin concentrations of night milking are approximately ten times compared to that in the daytime. In both preterm and term breastmilk, the melatonin concentration presented a circadian rhythm with the acrophase at around 03:00 (Qin et al., 2019). Melatonin has been found in colostrum with the comparable concentration as it in plasma (Meng et al., 2017). On this ground, mothers of preterm neonates will be asked to collect breastmilk between 01:00-05:00 a.m. at the three above mentioned time periods.

References

Brown RL, Fagundes CP, Thayer JF, Christian LM. Longitudinal changes in HRV across pregnancy and postpartum: Effect of negative partner relationship qualities. Psychoneuroendocrinology. 2021 Jul;129:105216. doi: 10.1016/j.psyneuen.2021.105216

Chen, Y. et al. (2016) Long-term measurement of maternal pulse rate dynamics using a home-based sleep monitoring system. *Journal of Sensors,* Article ID 5730142, https://doi.org/10.1155/2016/5730142

Hadas IM, Joseph M, Luba Z, Michal KL. Assessing parasympathetic measures of heart rate variability shortly after birth to predict motor repertoire at four months in low risk preterm infants born between

28 and 32 weeks of gestation. Early Hum Dev. 2021 Oct;161:105438. doi: 10.1016/j.earlhumdev.2021.105438
Kozar M, Tonhajzerova I, Mestanik M, Matasova K, Zibolen M, Calkovska A, Javorka K. Heart rate variability in healthy term newborns is related to delivery mode: a prospective observational study.
BMC Pregnancy Childbirth. 2018 Jun 27;18(1):264. doi: 10.1186/s12884-018-1900-4.
Musa Shaza M., Adam Ishag, Hassan Nada G., Rayis Duria A., Lutfi Mohamed F. (2017). Maternal Heart Rate Variability during the First Stage of Labor. Frontiers in Physiology, 8, https://www.frontiersin.org/articles/10.3389/fphys.2017.00774, doi:10.3389/fphys.2017.00774
Meng X, Li Y, Li S, Zhou Y, Gan RY, Xu DP, Li HB. Dietary Sources and Bioactivities of Melatonin. Nutrients. 2017 Apr 7;9(4):367. doi: 10.3390/nu9040367
Mulkey, S. B., du Plessis, A. J. (2019). Autonomic system development and its impact on neuropsychiatric outcome. *Pediatric Research, 85*(2), 120-126
Nguyen Phuc Thu T, Hernandez AI, Costet N, Patural H, Pichot V, et al. (2019) Improving methodology in heart rate variability analysis for the premature infants: Impact of the time length. PLOS ONE 14(8): e0220692. https://doi.org/10.1371/journal.pone.0220692
Peuhkuri, K., Sihvola, N., & Korpela, R. (2012). Dietary factors and fluctuating levels of melatonin. *Food and Nutrition Research*, 56: 17252.
Qin, Y., Shi, W., Zhuang, J. *et al.* (2019) Variations in melatonin levels in preterm and term human breast milk during the first month after delivery. *Scientific Reports,* 9, 17984.
Rowan SP, Lilly CL, Claydon EA, Wallace J, Merryman K. Monitoring one heart to help two: heart rate variability and resting heart rate using wearable technology in active women across the perinatal period. BMC Pregnancy Childbirth. 2022 Nov 30;22(1):887. doi: 10.1186/s12884-022-05183-z.
Sarhaddi F, Azimi I, Axelin A, Niela-Vilen H, Liljeberg P, Rahmani AM. Trends in Heart Rate and Heart Rate Variability During Pregnancy and the 3-Month Postpartum Period: Continuous Monitoring in a Free-living Context. JMIR Mhealth Uhealth. 2022 Jun 3;10(6):e33458. doi: 10.2196/33458.
Schlatterer SD, Govindan RB, Barnett SD, Al-Shargabi T, Reich DA, Iyer S, Hitchings L, Larry Maxwell G, Baker R, du Plessis AJ, Mulkey SB. Autonomic development in preterm infants is associated with morbidity of prematurity. Pediatr Res. 2022 Jan;91(1):171-177. doi: 10.1038/s41390- 021-01420-x
Tan CW, Tan NY, Sultana R, Tan HS, Sng BL. Investigating the association factors of acute postpartum pain: a cohort study. BMC Anesthesiol. 2023 Jul 25;23(1):252. doi: 10.1186/s12871-023-02214-w.
